# Optimizing Photoperiod Switch to Maximize Floral Biomass and Cannabinoid Yield in *Cannabis sativa* L.: A Meta-Analytic Quantile Regression Approach

**DOI:** 10.3389/fpls.2021.797425

**Published:** 2022-01-10

**Authors:** Michelle Dang, Nishara Muthu Arachchige, Lesley G. Campbell

**Affiliations:** ^1^Department of Chemistry and Biology, Ryerson University, Toronto, ON, Canada; ^2^College of Medicine, University of Saskatchewan, Saskatoon, SK, Canada

**Keywords:** photoperiod, crop yield, life history, resource allocation, quantile regression, cannabinoids, floral biomass, *Cannabis sativa*

## Abstract

*Cannabis sativa* L. is an annual, short-day plant, such that long-day lighting promotes vegetative growth while short-day lighting induces flowering. To date, there has been no substantial investigation on how the switch between these photoperiods influences yield of *C. sativa* despite the tight correlation that plant size and floral biomass have with the timing of photoperiod switches in indoor growing facilities worldwide. Moreover, there are only casual predictions around how the timing of the photoperiodic switch may affect the production of secondary metabolites, like cannabinoids. Here we use a meta-analytic approach to determine when growers should switch photoperiods to optimize *C. sativa* floral biomass and cannabinoid content. To this end, we searched through ISI Web of Science for peer-reviewed publications of *C. sativa* that reported experimental photoperiod durations and results containing cannabinoid concentrations and/or floral biomass, then from 26 studies, we estimated the relationship between photoperiod and yield using quantile regression. Floral biomass was maximized when the long daylength photoperiod was minimized (i.e., 14 days), while THC and CBD potency was maximized under long day length photoperiod for ~42 and 49–50 days, respectively. Our work reveals a yield trade-off in *C. sativa* between cannabinoid concentration and floral biomass where more time spent under long-day lighting maximizes cannabinoid content and less time spent under long-day lighting maximizes floral biomass. Growers should carefully consider the length of long-day lighting exposure as it can be used as a tool to maximize desired yield outcomes.

## Introduction

To maximize agricultural productivity, growers can manipulate, and optimize environmental conditions, and thus the timing of development, to shift allocation patterns toward desired yield outputs (Loomis et al., 1971; Stearns, 1992; Weiner, 2003, 2004). Crops grown indoors are unique in that their environmental conditions, like light quality and quantity and temperature, can be strictly controlled, compared to traditional outdoor farms where productivity is often limited by climatic conditions (Mills, 2012; Arnold, 2013; Banerjee and Adenaeuer, 2014; Barbosa et al., 2015). Photoperiodic crops, plants that align their development with the amount and timing of light they receive (Thomas and Vince-Prue, 1996; Jackson, 2009), require a specific lighting schedule to flower and thus produce harvestable materials. While long-day plants require increased amounts of day light to trigger reproductive growth, short-day plants demand decreased day light to shift resource allocation from vegetative to reproductive growth (Thomas and Vince-Prue, 1996; Jackson, 2009). Here we explore how an economically important, short-day crop, *Cannabis sativa* L., varies in yield when variation in the timing of photoperiod shifts occur.

Originally from Asia, *C. sativa* grows vigorously outdoors in a variety of latitudes and climates around the world (Long et al., 2017; McPartland et al., 2018, 2019). However, in countries where the plant is a controlled substance, *C. sativa* growers are often restricted to indoor cultivation for a variety of reasons including: climatic conditions may not suitable for all-year, outdoor growing (e.g., Canada), regulations that restrict outdoor cultivation (e.g., Greece, New Zealand, United Kingdom), or a combination of these reasons (Misuse of Drugs (Amendments) (Cannabis Licence Fees) (England, Wales and Scotland) Regulations, 2018; Brown and Blackburn, 1987; Misuse of Drugs (Medicinal Cannabis) Amendment Bill, 2018; Folina et al., 2019). Various countries mandate indoor cultivation of *C. sativa* which requires time, space, and energy to do so. In Canada alone, ~2 million square meters of space is licensed for indoor *C. sativa* cultivation (Government of Canada, 2021) that helps fuel this $2.2 billion industry (Statistics Canada, 2019). In the United States, the amount of electricity used to cultivate C. *sativa* is an estimated $6 billion dollars with lighting being the primary source of that cost (Mills, 2012; Arnold, 2013). Thus, optimizing the timing of development is key to minimizing costs in this burgeoning industry.

While *C. sativa* is often cultivated for its phytocannabinoids (i.e., secondary metabolites primarily concentrated in floral tissue that provide various medicinal and intoxicating effects), the plant can also be a source of oilseeds and fibers in its primary stems (Small and Marcus, 2002). The majority of the plant, particularly the upper surface of leaves and flowers, are coated with trichomes (Happyana et al., 2013; Spitzer-Rimon et al., 2019) that produce cannabinoid precursors and ultimately cannabinoids (De Backer et al., 2012; Burgel et al., 2020). Unpollinated pistillate flowers (i.e., *sensimilla*) are the portion of the plant harvested for recreational and biopharmaceutical use as these inflorescences contain up to 10 times more cannabinoids in them than vegetative tissue (Bernstein et al., 2019). Phytocannabinoid yield is driven by both the floral biomass produced by a plant, as well as the concentration of two important cannabinoids: delta-9-tetrahydracannabinol acid (THCA) and cannabidiolic acid (CBDA) (ElSohly and Slade, 2005). While cannabigerolic acid (CBGA) accumulates during the vegetative phase growth in *C. sativa* and is the biosynthetic precursor to THCA, CBDA, and other cannabinoids (Taura et al., 1995, 1996; De Backer et al., 2012; Burgel et al., 2020), THCA and CBDA are the most commercially valued and therefore most focused on. While THCA and CBDA are the cannabinoids that accumulate in plant tissue, only when these compounds are heated do they become psychoactive Δ^9−^-tetrahydrocannabinol (THC) and cannabidiol (CBD), respectively (Doorenbos et al., 1971; De Backer et al., 2012; Chandra et al., 2017a). While the concentration of cannabinoids in *C. sativa* is largely influenced by environmental and horticultural factors (Knight et al., 2010; Potter and Duncombe, 2012; Caplan et al., 2017; Magagnini et al., 2018; Backer et al., 2019), genetics also plays a large role in the diverse chemotypic outcomes of the plant (de Meijer et al., 2003; Hillig and Mahlberg, 2004; Lynch et al., 2016; Welling et al., 2016; Campbell et al., 2020).

The life-cycle of industrial *C. sativa* can be divided into four stages: (1) germination/cloning, (2) vegetative growth, (3) flowering and seed formation, then, (4) senescence (Mediavilla et al., 2001). During vegetative growth, plants are exposed to at least 18 h of long-day lighting (Clarke, 1981) to promote stem and leaf growth (Mediavilla et al., 2001). To induce floral development and the conversion of CBGA to either THCA or CBDA, the daylength is shortened to ~12 h or less. Thus, the scheduling of photoperiod switch (i.e., the timing of when the photoperiod switches from long to short days) will drive the size and number of flowers as well as the abundance of cannabinoids to influence yield (Borthwick and Scully, 1954; Bocsa and Karus, 1998; Potter, 2014). Various studies show that cannabinoids accumulate in leaf and floral matter of drug-type *C. sativa* between growth periods differently over time. Specifically, while vegetative biomass (i.e., leaves, stems) slowly accumulates small amounts of cannabinoids over a plant's lifespan (Pacifico et al., 2008; Aizpurua-Olaizola et al., 2016; Richins et al., 2018), cannabinoid concentration in inflorescences increase more intensely with more days spent in flowering growth (King et al., 2004; Pijlman et al., 2005; De Backer et al., 2012; Aizpurua-Olaizola et al., 2016; ElSohly et al., 2016; Richins et al., 2018; Yang et al., 2020). In contrast, plants maintained in vegetative lighting conditions continue to increase in vegetative biomass and rarely flower (Borthwick and Scully, 1954; Moher et al., 2021). As a result, indoor cannabis cultivators must carefully plan out the timing of photoperiodic switches to meet the day length demands of these photoperiod-sensitive plants. While scholarly articles and the gray literature outline ideal lengths of time *C. sativa* plants should spend in vegetative and flowering growth periods, photoperiod durations vary between sources with no unclear explanations as to how different photoperiod lengths influence yield outcomes (Cervantes, 2006; Chandra et al., 2017b; Goggins and Hennings, 2020). Given that *C. sativa* has two valuable yield outputs, if cannabinoid production is not tightly correlated with floral biomass allocation, lighting manipulations that maximize floral biomass may not necessarily maximize chemical yield and vice versa. To our knowledge, the industry lacks information from controlled experiments to determine the number of days a plant should spend in long-day lighting to maximize yield. Using evidence-informed best practices in cannabis cultivation allows growers to minimize costs and resources while maximizing indoor crop yield. Here we addressed this gap in the literature to define photoperiod duration practices that maximizing cannabinoid and floral yield in *C. sativa*.

Our analysis aimed to define how *Cannabis sativa* L. yield varies under different photoperiodic switch practices when cultivated indoors. To that end, we asked: How long do growers place *C. sativa* plants under long-day lighting? Is the optimal time spent under long-day lighting different for maximal floral biomass production vs. maximal cannabinoid concentration in inflorescences? What is the optimal length of time a *C. sativa* plant should spend in long-day lighting, such that producers maximize both components of yield and yet minimize time spent in this growth stage? By using a meta-analysis approach to address these questions, we can review the photoperiod practices used by a broad range of *C. sativa* cultivators and find trends in the yield outcomes they achieved. We argue that time spent in vegetative growth (controlled by long-day photoperiod) is positively correlated with cannabinoid content, but negatively correlated with floral biomass, as long-day lighting periods favor development of vegetative cannabinoids but delay reproductive floral development.

## Materials and Methods

### Review Protocol

We used several data sources to gather quantitative evidence from the literature on the impact of the timing of when farmers switched from long to short day photoperiods on the (1) harvested biomass and (2) concentration of cannabinoid content of *C. sativa*. To find relevant articles, we searched the following search on ISI Web of Science database on February 2, 2021: “Cannabis yield photoperiod,” “Cannabis lighting,” “Cannabis flowering,” “Cannabis day length,” “Cannabis photoperiod,” “Photoperiodic hemp” (Supplementary Table S1). We also screened articles from our lab group and reference lists from published articles for relevant publications.

To be included in our dataset, we reviewed each article against inclusion criteria. Our inclusion criteria required that studies of *C. sativa* report: (1) harvested yield as floral biomass and/or cannabinoid concentration; (2) the number of days spent under long day length lighting during the vegetative growth stage; (3) the timing of a definitive switch between long day (≥18 h of light) to short-day lighting (≤12 h of light) conditions for *C. sativa* (rather than a gradual change in photoperiod as might occur outdoors). To screen for this information, we skimmed through abstracts for keywords, and if papers appeared to contain information from our inclusion criteria, we set them aside for further review. From the smaller subset of papers, we then thoroughly read through the methods and results sections to identify whether papers included the data we needed. Studies that involved *C. sativa* cultivation under outdoor, natural daylight environments were excluded from this analysis as they lacked distinct photoperiod stages which violated inclusion criteria 1 and 3. Additionally, studies that reported lighting durations as a range of days (rather than a single date) were excluded. A summary of our screening process is provided in Supplementary Figure S1. We collected these pieces of information from each paper, as well as sample size and estimates of errors around the mean if available. Where an author measured yield under experimentally manipulated environmental conditions (e.g., addition of UV light), only yield under the least manipulated condition was considered (i.e., generally designated as controls in a study).

### Data Extraction

Although we considered a sizable collection of articles (*n* = 1,009 studies, Supplementary Table S1), 26 studies met our criteria for inclusion. We extracted the mean, statistical error (usually standard error or deviation), and sample size values for each yield variable under lighting conditions from tables, text, or from graphs using ImageJ software (NIH, imagej.nih.gov/ij/) (Supplementary Datasheet S1). If multiple cultivars were used within the control condition, we treated each cultivar as independent for analysis. We then collated each yield measure across control conditions with the number of days the plants were kept under long-day lighting conditions. All reported floral biomass measurements were converted to and expressed as grams of dry inflorescences per plant, while all cannabinoid measurements were expressed as percent cannabinoids in dried inflorescence. If major cannabinoids were separated into THC and THCA, or CBD and CBDA, we recorded the sum of these cannabinoids as the total THC and CBD yield, respectively. Although we collected information on cannabinoids other than THC and CBD, there was not enough published data for a robust analysis of minor cannabinoids. To identify outliers in our long-day lighting duration and yield measures, we did Rosner's test (Rosner, 1983) using the “EnvStats” package (Millard, 2013) in R (version 1.3.1093). Three data points were removed from long-day lighting duration, two data points were removed from floral biomass, and 15 were removed from CBD concentration. Notably, the 15 CBD datapoints were all above 1% CBD; therefore, the dataset analyzed for CBD concentrations reflect values that range from 0 to 1%.

### Regression Analysis

**Figure 1:**
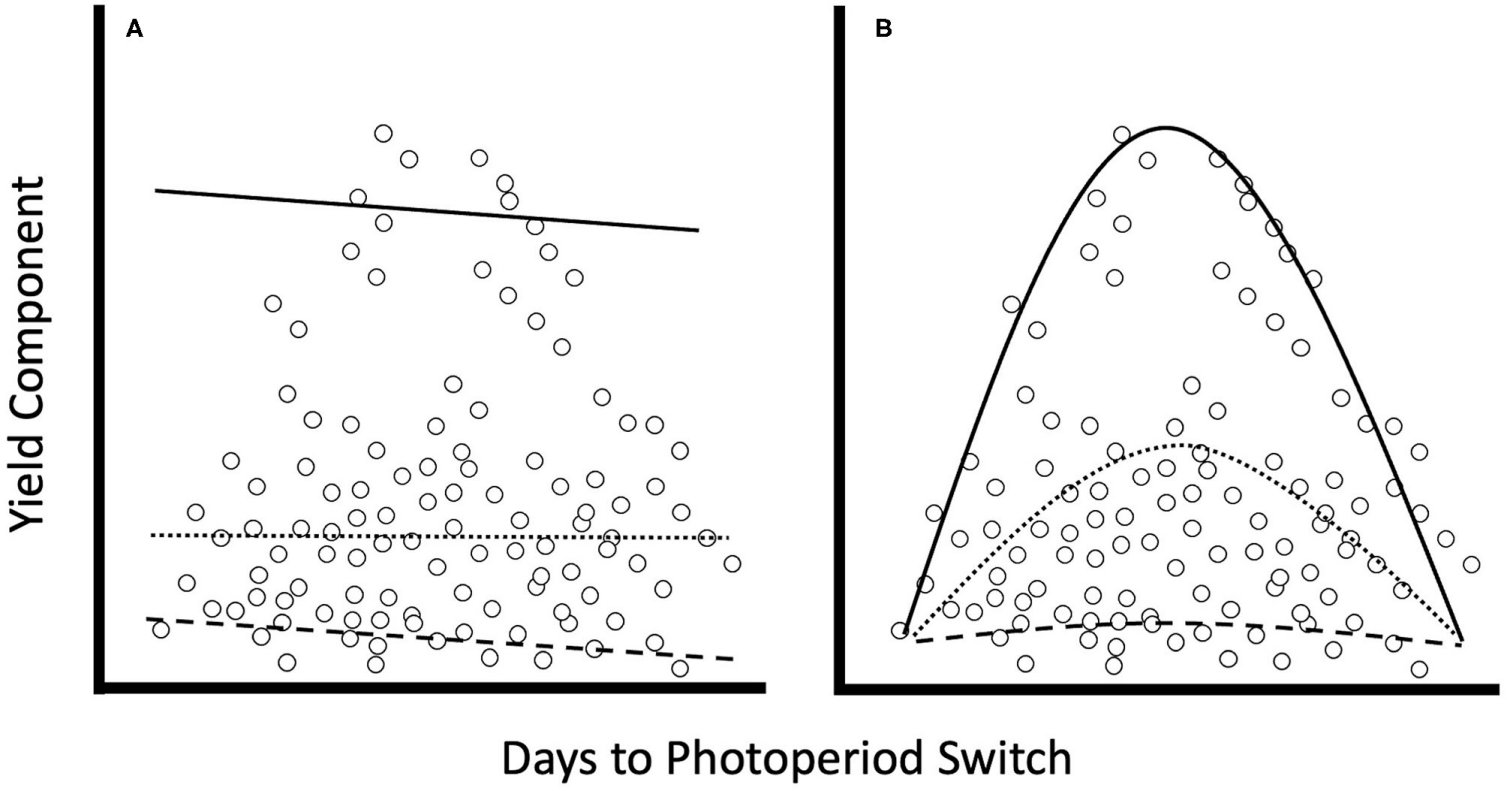
Graphs comparing trendlines for linear vs. quadratic quantile regression (After this figure in Mills and Waite, 2009). When comparing yield from plants grown across a wide range of environments, we cannot expect that plants will exhibit the same average outcomes under different environmental conditions. Thus, quantile regression allows us to model the worst performance (5th quantile = dashed line), average performance (50th quantile = dotted line), and the best performance (95th quantile = solid line), given a particular environmental state for both **(A)** linear and **(B)** quadratic models.

To determine the relationship between the number of days of long-day lighting and the various yield components, we performed three regression analyses: simple linear regression, linear quantile regression, and non-linear quantile regression. Quantile regression is a statistical method for estimating the correlative relationship between variables based upon conditional medians (Koenker and Bassett, 1978). While simple, least-square, linear regression techniques use the mean to create a model that minimizes sums of squared residuals, quantile regression creates models using conditional medians to minimize sums of squared residuals instead (Koenker and Geling, 2001). In other words, linear regression uses the conditional mean of a dataset to create a predictive model, a quantile regression at the 50th quantile uses values surrounding the median of the dataset for its modeling. Quantile regression is particularly robust in its use on non-parametric datasets that exhibit heterogeneous variance (Koenker and Bassett, 1978; Cade and Noon, 2003). Life-history relationships often differ across probability distributions and so quantile regression allows us to explore how relationships change at different bounds of a dataset (Figure 1; Scharf et al., 1998; Cade and Noon, 2003). Moreover, linear quantile regressions do not always best describe the relationships between the response and predictor variables, so non-linear quantile regressions can be used instead (Figure 1; Cade and Noon, 2003; Mills and Waite, 2009). Here, we compare the ability of simple linear, linear quantile, and non-linear quantile regressions to describe the relationship of when photoperiod switches from long- to short-day periods with yield.

We did statistical analysis in R using the packages “stats” (R Core Team, 2020) and “quantreg” (Koenker, 2021). To describe the relationship of duration of long day length and yield outcome (e.g., floral biomass, THC concentration, CBD concentration), we first performed simple linear regression using the “lm()” function. Then, we used the “rq()” and “nlrq()” functions to perform linear and non-linear quantile regressions, respectively, for each parameter at three quantiles (τ): 0.50, 0.75, and 0.95; because we wanted to identify the timing of photoperiod switch that maximized yield outcomes, we chose to create predictive models of three quantiles ranging from “average,” “medium-high,” to “high” performing levels of yield across studies. For the linear regression analyses, the following model was used:


(1) $$\begin{array}{l} \text{ϕ}\left({\text{D}}_{\text{i}}\right)=\text{α}+{\text{β}}_{1}\left({\text{D}}_{\text{i}}\right)+\text{ε} \end{array}$$

For the non-linear quantile regression analyses, the following quadratic model was used:


(2) $$\begin{array}{l} \text{ϕ}\left({\text{D}}_{\text{i}}\right)=\text{α}+{\text{β}}_{1}\left({\text{D}}_{\text{i}}\right)+{\text{β}}_{2}{\left({\text{D}}_{\text{i}}\right)}^{2}+\text{ε} \end{array}$$

Here, ϕ (D_i_) represents the predicted yield outcome (e.g., floral biomass, % THC, % CBD) as a function of D_i_–the number of days spent under long day length. The regression coefficients are represented as β_1,2…*n*_, the y-intercept is represented by α, while ε represents the residual error. While the linear models may find an overall, constant trend in how yield measures respond to varying days under long day length lighting, the quadratic model can provide a more nuanced model to predict yield and identify a definitive optimum number of days to maximize each yield outcome. Specifically, we solved for the global optima from our quadratic equations to identify optimal days until photoperiod switch that maximize specific yield outcomes.

## Results

### Patterns in Photoperiod Switches and Yield Outcomes

Growers cultivated *C. sativa* under a variety of durations under long daylength light conditions which resulted in diverse yield outcomes (Table 1). Growers cultivated plants under long day length lighting at a range of 13–180 days, with the mean, median, and mode number of vegetative days being 37.1, 30, and 21 days, respectively. Jin et al. (2020) kept plants in vegetative growth for 180 days, an identified outlier, which was at least double the vegetative duration of other studies.

**Table 1:** Characteristics of photoperiod regimens and yield outcomes (floral biomass, % THC, % CBD) for *C. sativa* across studies (26 unique studies, including outliers).

**Variables**	**Sample size** **(***n***)**	**Average**	**Median**	**Mode**	**Range**
Duration of long day lighting (days)	29	37.1 ± 4.1	30.0	21.0	13.0–180
Floral biomass (g/plant)	31	43.1 ± 14.3	24.1	27.8	8.6–445.2
THC (%)	53	12.3 ± 1.0	14.4	15.9	0.1–26.1
CBD (%)	48	2.3 ± 0.7	0.4	0.0	0–18.8

*While photoperiod switch statistics were calculated using each unique long day lighting duration within and between studies, statistics for physical and chemical yield were calculated based on all individually reported cultivars. Standard errors (SE) are presented for each average value*.

The amount of harvestable floral biomass between growers varied at a range of 8.6–445.2 g, with the mean, median, and mode being similar at around 12–16 g (Table 1). Rosner's test identified the study conducted by Knight et al. (2010) as an outlier, obtaining a yield of 445.2 g per plant, at least three times greater than yields of any other study in this dataset. THC concentrations ranged from 0.1 to 26.1%, while CBD concentrations ranged from 0 to 18.8% (Table 1). The mean, median, mode for % THC were similar at values between 12.3 and 15.9%, these values for % CBD were also similar at values between 0 and 2.3% (Table 1).

### Regression Models for Predicting and Optimizing Yield

**Figure 2:**
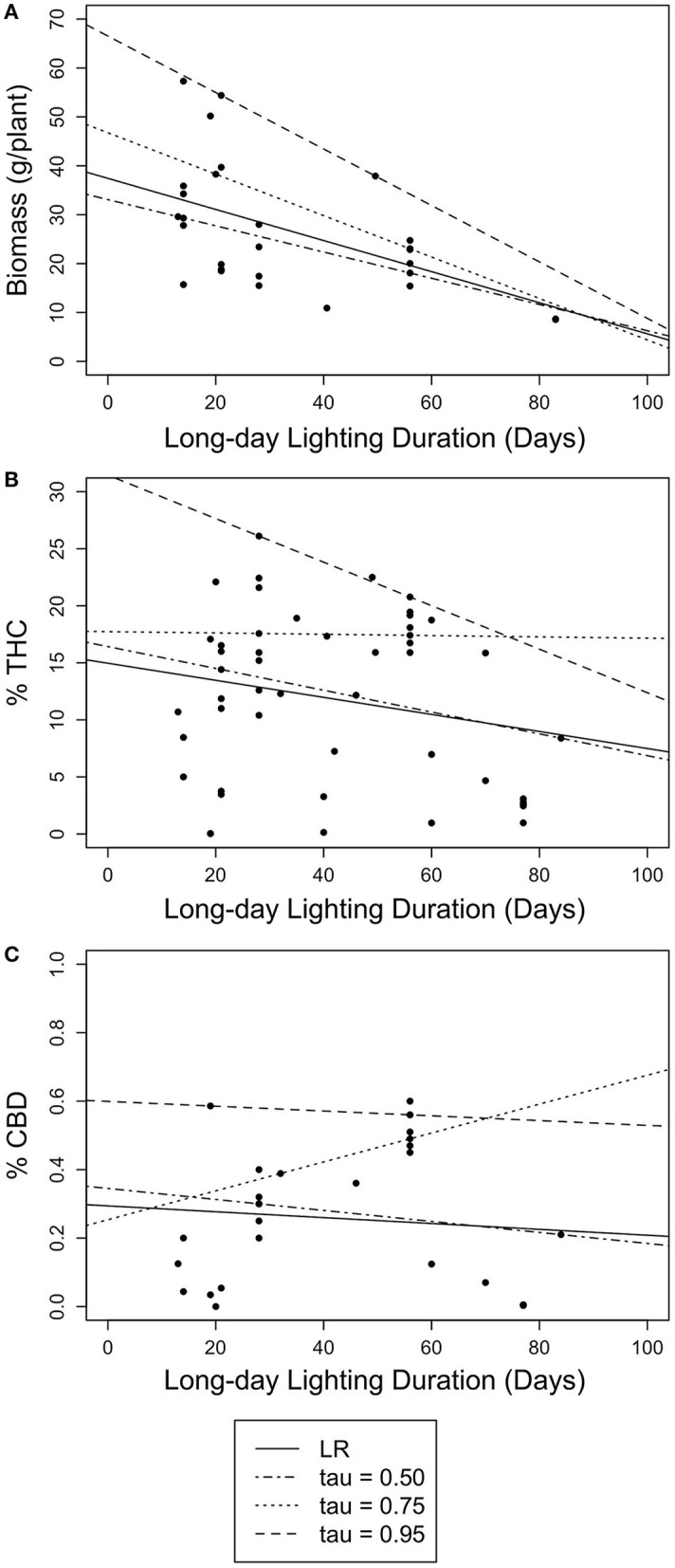
Scatter plots of linear quantile regression results for long and short daylength durations (days) with floral biomass (g/plant) and cannabinoid concentration (% THC and % CBD). Lines represent simple linear regression (LR) (solid) and 50th (dotted-dashed), 75th (dotted), and 95th (dashed) linear quantile regressions. **(A)** The relationship between long daylength duration and floral biomass (g/plant). **(B)** The relationship between long daylength duration and THC concentration (% THC). **(C)** The relationship between long daylength duration and CBD concentration (% CBD).

Only floral biomass was significantly, negatively correlated with timing of photoperiod switch when using both simple linear regression [Adj.R^2^ = 0.2555, β = −0.3180, *F*_(1,27)_ = 10.61, *p* < 0.005] and linear quantile regression at the 50th at the 50th, 75th, and 95th quantile (β = −0.2681, *p* < 0.017; β = −0.4246, *p* < 0.001; β = −0.5772, *p* < 0.001), even after adjusting for multiple hypothesis testing (Bonferroni corrected using α = 0.017; Bonferroni, 1936). Despite these significant relationships, the explanatory power of these models is low (Tables 1, 2; Figure 2); highlighting the limitation of linear model approaches as a tool to predict complex yield outcomes. These results also show how simple linear regression models can lead to different results than quantile linear regression. While the linear models were able to predict floral biomass, they were not able to find significant relations for cannabinoid content, nor identify any optimum number of vegetative days to maximize yield (Table 1, Figure 2). Next, we turned to quadratic quantile regression for additional analysis.

**Table 2:** Linear quantile regression of the timing of photoperiod switches with floral biomass (*n* = 29), THC concentration (*n* = 50), and CBD concentration (*n* = 31).

**Yield measure**	**Simple linear regression**	**Correlation coefficients (SE) at the 50th, 75th, and 95th quantiles**		
		**0.50**	**0.75**	**0.95**
Floral biomass	−0.3180 (0.0976) [0.2555]^**^	−0.2681 (0.0996)^*^	−0.4236 (0.1503)^***^	−0.5772 (0.1422)^***^
THC	−0.0748 (0.0487) [0.0271]	−0.0955 (0.1208)	−0.0057 (0.0819)	−0.1907 (0.0955)
CBD	−0.0009 (0.0016) [−0.0248]	−0.0016 (0.0046)	0.0042 (0.0042)	−0.0007 (0.0041)

Correlation coefficients (β) are reported for each yield measure at quantiles (τ): 0.50, 0.75, and 0.95 with their standard error in parentheses, as well as the adjusted R-squared in square brackets for the simple linear regression model. To avoid alpha inflation, p-values were reported for each relationship using α = 0.017 using Bonferroni's correction (Bonferroni, 1936); bolded values represent significant relationships with asterisks denoting levels of significance (^*^ <0.017, ^**^ <0.005, ^***^ <0.001).

**Figure 3:**
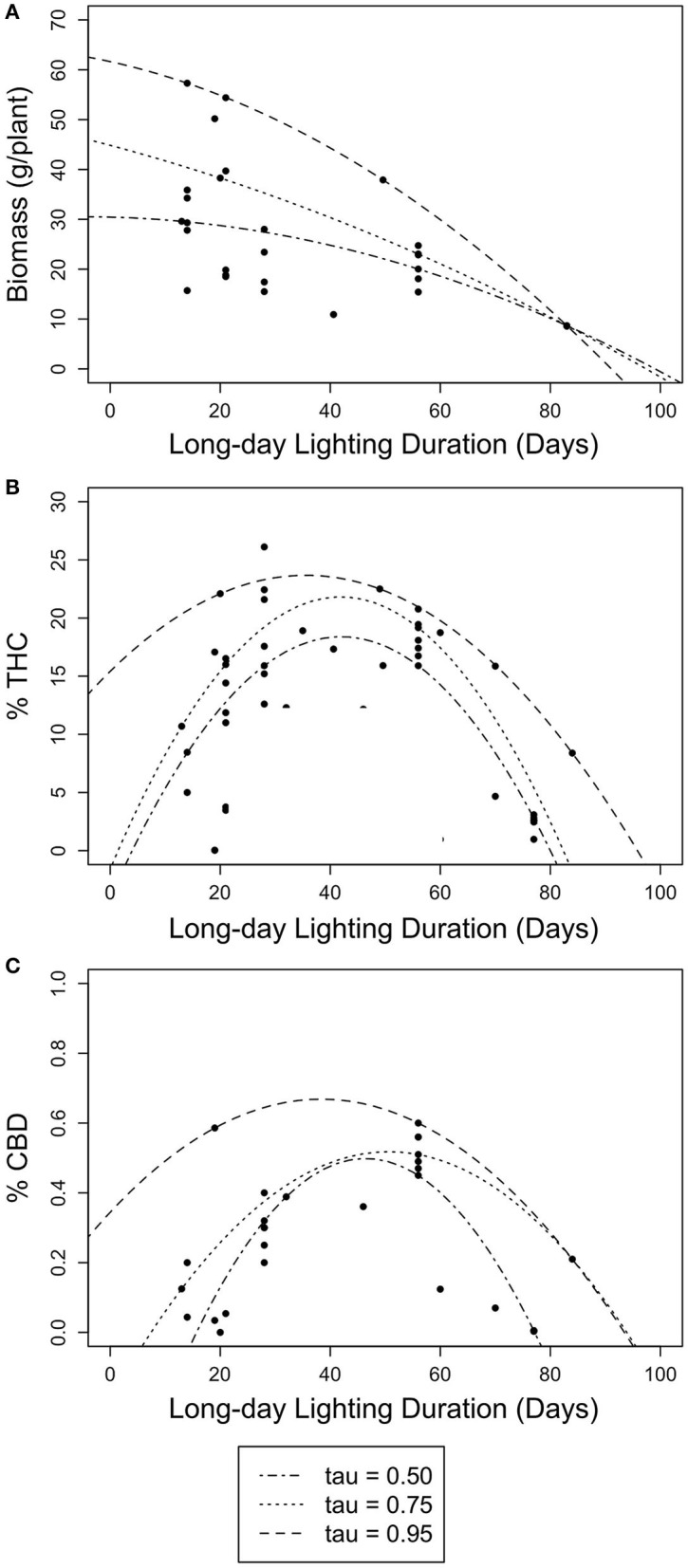
Scatter plots of non-linear quantile regression results for long day length durations (days) with floral biomass (g/plant) and cannabinoid concentration (% THC and % CBD). Lines represent 50th (dotted-dashed), 75th (dotted), and 95th (dashed) quantile regressions. **(A)** The relationship between long day length duration and floral biomass (g/plant). **(B)** The relationship between long day length duration and THC concentration (% THC). **(C)** The relationship between long day length duration and CBD concentration (% CBD).

The quadratic model results presented here provide the first robust estimate of the optimal timing of photoperiod switch for *C. sativa* developed using the scientific method (rather than trial and error). The relationships between photoperiod switch and each yield estimate were similar across the three quantiles of the quadratic models of the timing of photoperiod switch and each estimate of yield (Table 3, Figure 3). While the quadratic model had little predictive power for the relationship between the timing of photoperiod switch and floral biomass, the quadratic quantile models identified various significant relationships between long day lighting and either THC or CBD. Specifically, significant, concave down parabolic relationships were found for THC and CBD at the 50th and 75th quantiles. This model outperformed the linear and linear quantile regression models. When we search for the local maxima, the optimal time to switch photoperiods to maximize THC is ~42 days based on the 50th and 75th quantile model, while CBD is maximized at about 49 days based on the 50th quantile model and 50 days based on the 75th quantile model.

**Table 3:** Non-linear (quadratic) quantile regression of the timing of photoperiod switches with floral biomass (*n* = 29), THC concentration (*n* = 50), and CBD concentration (*n* = 31).

**Yield measure**	**Non-linear regression equations at the 50th, 75th, and 95th quantiles**		
	**0.50**	**0.75**	**0.95**
Floral biomass	−0.0028a – 0.0284b + 30.45	−0.0017a – 0.2935b + 44.83	−0.0048a – 0.2424b + 61.64
THC	−0.0127a^***^+ 1.069b^***^ – 4.062	−0.0133a^***^+ 1.116b^***^ – 1.561	−0.0065a + 0.4638b + 15.42
CBD	−0.0005a^***^+ 0.0490b^***^ – 0.6386	−0.00028a + 0.0281b^*^ – 0.1931	−0.0002a + 0.0169b – 0.3445

Correlation coefficients (β) are reported for each term in the quadratic model for each yield measure at quantiles (τ): 0.50, 0.75, and 0.95. We have retained 4–5 significant digits to offer accurate quadratic models. To avoid alpha inflation, p-values were adjusted for each a and b term in the quadratic model at α = 0.017 using Bonferroni's correction (Bonferroni, 1936); bolded values represent significant relationships with asterisks denoting levels of significance (^*^ <0.017, ^**^ <0.005, ^***^ <0.001).

## Discussion

### When Do Growers Switch Photoperiod to Induce Flowering?

Our results uncovered a lack of consistency in photoperiod switch practices and the underuse of photoperiod as a yield optimization tool. While various academic cultivators recommend a vegetative growth period of 14–21 days (Cantin, 2017; Chandra et al., 2017b), the gray literature recommends that vegetative growth continue for 3–16 weeks (Cervantes, 2006; Goggins and Hennings, 2020; Haze, 2021). Notably, the reasoning behind academic and grower recommendations was not justified in the sources. We predict growers may choose long day lighting periods based on their personal preference and qualitative judgements on plant maturity that maximize plant size but minimizes cultivation time and resources. Also, as increased vegetative size is correlated with floral biomass and therefore total amount of cannabinoids harvested (de Meijer et al., 2003; Potter, 2014; Bernstein et al., 2019; Danziger and Bernstein, 2021), growers may choose a vegetative growth duration that maximizes plant size and branching which may consequently maximize total cannabinoid content. Alternatively, there is also the possibility that growers place plants under long-day lighting durations in response to recommendations offered by seed producers (i.e., vegetative durations on seed labels, on producer websites, communicating directly with producer). The diversity in long day lighting durations and the lack of clear reasoning as to why growers choose to switch photoperiods illustrates how conflicting cannabis cultivation standards are due to a lack of empirical studies on best practices.

### Using Regression Models for Describing the Relationship Between Lighting Duration and Yield Outcomes

When using long day lighting to predict yield outcomes, floral biomass is best described using linear regression models (either simple or quantile) and cannabinoid content is best described using a quadratic quantile regression model (Figures 2, 3; Tables 1, 2). For each linear quantile regression, floral biomass was significantly predicted by the duration of long day lighting at all three quantiles which lends to the validity of these models. Floral biomass was better described using a linear model and not a quadratic model which was consistent with other studies that have found similar results although using different predictive factors (Toonen et al., 2006; Potter and Duncombe, 2012; Eaves et al., 2020; Yep et al., 2020; Rodriguez-Morrison et al., 2021). In contrast, our quadratic models to describe the relationships between cannabinoid potency and long day lighting duration are novel insights relative to previous studies describing the relationship between environmental conditions and chemical yield in *C. sativa* with linear models (de Meijer et al., 2003; Toonen et al., 2006; Westerhuis et al., 2009; Potter and Duncombe, 2012; Burgel et al., 2020; Petit et al., 2020). Plant yield does not always linearly respond to environmental conditions (Holmgren et al., 2011; Paine et al., 2012; Archontoulis and Miguez, 2015) and so fitting yield data to a linear model can lead to weak predictive power. Recent studies have suggested that the relationship between *C. sativa* development over time is non-linear, specifically quadratic or sigmoidal (Vanhove et al., 2017; Stack et al., 2021). Given our models came back significant and the support for using non-linear models to fit ecological data (Park et al., 2005; Mills and Waite, 2009; Konduri et al., 2020), using quadratic model to describe cannabinoid potency and long day lighting is useful to this investigation. This highlights how important using non-linear models to model ecological trends are to uncovering relationships between environmental conditions and plant outcomes, especially so in *C. sativa* where linear models are still prevalent. As a result, we do not endorse using linear simple or quantile regression models to predict yield from the date when photoperiod was switched.

### Optimizing Floral Biomass and Cannabinoid Potency Using Best Photoperiodic Switch Practices

The optimal duration of long day lighting exposure to maximize floral biomass compared to THC and CBD concentration in *C. sativa* are different. Specifically, floral biomass and long day light duration shows a negative linear relationship suggesting that shorter vegetative growth periods are preferable for maximizing yield. In contrast, THC and CBD show a negative quadratic relationship with long day length durations in which cannabinoid potency only increases so much before yield beings to decrease with increasing days to photoperiod switch. This trend in cannabinoid development is consistent with what we currently know about cannabinoid biosynthesis—*C. sativa* plants need time to make CBGA that later converts to THCA and CBDA but after a certain point, cannabinoid concentration begins to decrease (Aizpurua-Olaizola et al., 2016; Burgel et al., 2020). We hypothesize that this trend is due to THCA and CBDA possibly being diluted in a larger amount of plant biomass when longer vegetative periods are used to grow *C. sativa*. Alternatively, major cannabinoids have been observed to degrade into cannabinol as *C. sativa* ages (Ross and ElSohly, 1997; Jaidee et al., 2021).

Given that floral biomass and cannabinoid yield are maximized at different long day lighting practices, this reveals a conflict or trade-off where best growing practices to maximize one component of yield will not necessarily optimize additional components of yield. This tradeoff may be explained by the lifespan of annual plants; given that annuals have a limited time to grow and reproduce before they die at the end of the growing season, they must adjust how they partition resources to vegetative vs. reproduction processes under different environmental conditions (Cohen, 1976; Bazzaz et al., 1987; Lundgren and Des Marais, 2020). Specifically, when growing in stressful environments with a potentially reduced growing season or poor growing conditions (e.g., delayed sowing date or seedling emergence, or light conditions for photoperiodic plants), plants can allocate resources earlier or more intensely to reproduction to maximize their success before the end of the season (Zhou et al., 2005; Franks et al., 2007; Hansen et al., 2013; Mason et al., 2017). In contrast, plants growing in conditions that allow for a long growing season will relatively allocate more resources to vegetative processes and less to reproductive processes. Alternatively, the trade-off we observed may be explained using several plant defense hypotheses which all imply that allocating resources to defense mechanisms, although necessary for survival, are costly and divert resources away from other traits like reproduction (Loomis, 1932; Rhoades, 1979; Herms and Mattson, 1992; Mole, 1994; Stamp, 2003). Given that floral biomass is a reproductive trait and cannabinoid production is a defense trait, the resources that *C. sativa* plants allocate toward one of these traits may be different under various environmental conditions. Shorter vegetative growth periods may signal low herbivory risk to plants and so they allocate less resources to cannabinoid production and more to reproduction growth, with the opposite being true for plants grown in longer vegetative growth periods. These theories could explain the yield trade-off observed here between cannabinoid concentration vs. floral biomass in which *C. sativa* demonstrates a life-history strategy to maximize cannabinoid content in longer vegetative periods and maximize floral biomass in shorter vegetative periods. Growers must then make the careful decision of choosing a vegetative photoperiod duration that maximizes one yield measure or optimizes each yield measure despite trade-offs in both.

Based on our findings, growers should keep plants in vegetative growth for as few days as possible to strongly establish the plant (the minimum number of days recorded in our study was 13) to optimize biomass production. The decreasing relationship between floral biomass and vegetative growth duration illustrates how *C. sativa* can plastically respond to particular environmental conditions; specifically, how photoperiod can be used to maximize floral biomass. If the goal is to maximize cannabinoid potency, this data shows that growers should keep plants in the vegetative stage for longer durations up until 50 days. The existing literature shows that growers, on average and most commonly, cultivate plants under long day lighting that fits within this window; however, various growers maintain vegetative growth for beyond 50 days (Table 1, Supplementary Datasheet S1). Despite growers potentially missing out on higher potential yield based on our models, cannabinoid potencies overall for studies included in our sample are consistent with *C. sativa* cultivated in the last decade (Richins et al., 2018; Chandra et al., 2019; ElSohly et al., 2021).

When choosing the yield outcome to optimize in *C. sativa*, growers must consider that industrial yield priorities change in response to the legal limits to carrying and consumption. Canada, for instance, permits residents to carry up to 30 g of dried *C. sativa* per day but does not regulate the concentration of cannabinoids in the prescription (and this weight-based approach seems to be the norm for the majority of North America) (Cannabis Act, 2018; Lancione et al., 2020). In such a system, cultivators would benefit from growing plants using photoperiodic schedules that maximize biomass yield, not cannabinoid yield. In contrast, professionals who prescribe *C. sativa* prescribed for medicinal use often suggest consumption based on cannabinoid concentration (i.e., mg of THC or CBD per serving) compared to grams consumed (Devinsky et al., 2018; Health Canada, 2018; MacCallum and Russo, 2018; Stockings et al., 2018; Millar et al., 2019; Laczkovics et al., 2021). Here, photoperiodic schedules that optimize cannabinoid concentration is more valuable than maximizing yield.

While this study reveals an interesting path forward for maximizing yield in *C. sativa*, there are a couple features of our analysis that limit our generalizations. First, our dataset came from indoor lab facilities where *C. sativa* cultivation was primarily completed by academic scientists. Research horticultural practices may not parallel the practices of *C. sativa* commercially or recreationally grown by horticulturalists. Further, although we only included control condition plants in this analysis, differences in horticultural and environmental conditions could create extraneous variation in yield outcomes between studies. Moreover, our study specifically looked at the photoperiod schedules of indoor growers, thus our results cannot be applied to predict how outdoor, natural seasonal day length influences yield. We also removed outliers from our dataset which decreased the range of long day lighting conditions and yield outcomes we could include in our model; therefore, our model is only applicable to yield outcomes and long day length lighting durations within this particular range. Most notably, our model for predicting CBD concentration is only applicable to concentrations below 1% and we encourage future researchers to explore how plants that produce high concentrations of CBD respond to the timing of photoperiodic switches. Lastly and most importantly, although this analysis describes yield outcomes as solely a result of long day lighting duration, we could not control for genetic differences between cultivars that extraneously influenced our variables of interest. Different cultivars can have different recommended number of vegetative days and have varying limits on how much biomass and cannabinoids they can produce due to genetics and environmental conditions. As a result, cultivar differences are a source of variability in our study and future work on this topic should control for genotype. We encourage future growers to test the hypotheses we have proposed, namely that shorter periods of vegetative growth will maximize floral biomass and that intermediate periods of vegetative growth maximize cannabinoid concentrations in plants. This serves as a solid call-to-action, to explore how to consistently produce plant-based medicines using photoperiod as one horticultural management tool.

## Conclusion

In conclusion, our analysis predicts that floral biomass and cannabinoid concentration of *C. sativa* can be maximized by growing plants under different long day length lighting durations: floral biomass is optimized when long day lighting duration is minimized, THC concentration is optimized at 42 days of long day lighting, and CBD in low CBD plants is optimized a 49–50 days of long day lighting. As yield outcomes are maximized at differing durations, growers must make careful decisions on when to switch photoperiod to optimize the multiple yield measures of *C. sativa*. Quantile regression is an easy and useful way of modeling the relationship between environmental conditions and crop yield that should be used more frequently to predict *C. sativa* yield outcomes, especially so at differing performance quantiles. There needs to be a continued effort to define best practices in *C. sativa* cultivation not only to minimize cultivation costs, but to consistently produce quality plant product.

## Data Availability Statement

The original contributions presented in the study are included in the article/Supplementary Materials, further inquiries can be directed to the corresponding author.

## Author Contributions

LC and MD contributed to the experimental design. MD and NA collected the data. MD analyzed the data as well as prepared figures and tables. MD and LC wrote and MD, LC, and NA revised the manuscript. All authors contributed to the article and approved the submitted version.

## Funding

The authors gratefully acknowledge funding from the Natural Sciences and Engineering Research Council of Canada (NSERC) Discovery Grants program (Nos. 402305-2011 and #05780-2019 to LC), NSERC Canada Graduate Scholarship—Master's program (to MD), and Ryerson University (for a research internship to NA).

## Conflict of Interest

The authors declare that the research was conducted in the absence of any commercial or financial relationships that could be construed as a potential conflict of interest.

## Publisher's Note

All claims expressed in this article are solely those of the authors and do not necessarily represent those of their affiliated organizations, or those of the publisher, the editors and the reviewers. Any product that may be evaluated in this article, or claim that may be made by its manufacturer, is not guaranteed or endorsed by the publisher.

## Abbreviations

THCA: tetrahydrocannabinolic acid
CBDA: cannabidiolic acid
CBGA: cannabigerolic acid
THC: Tetrahydrocannabinol
CBD: cannabidiol.
