# Supplementary material for: Optimizing Photoperiod Switch to Maximize Floral Biomass and Cannabinoid Yield in Cannabis sativa L.: A Meta-Analytic Quantile Regression Approach
Source: Front Plant Sci. 2022 Jan 10;12:797425. doi: 10.3389/fpls.2021.797425 (PMC8786113; doi:10.3389/fpls.2021.797425)
Supplement: Supplementary file 1 [file Data_Sheet_1.DOCX]

Supplementary Materials

**Title:** Optimizing Photoperiod Switch to Maximize Floral Biomass and Cannabinoid Yield in *Cannabis sativa* L.: A Meta-Analytic Quantile Regression Approach

Authors: Michelle Dang, Nishara Muthu Arachchige, Lesley G. Campbell^*^

* Corresponding Author: [lesley.g.campbell@ryerson.ca](mailto:lesley.g.campbell@ryerson.ca)

This file includes:

Figure S1; Table S1


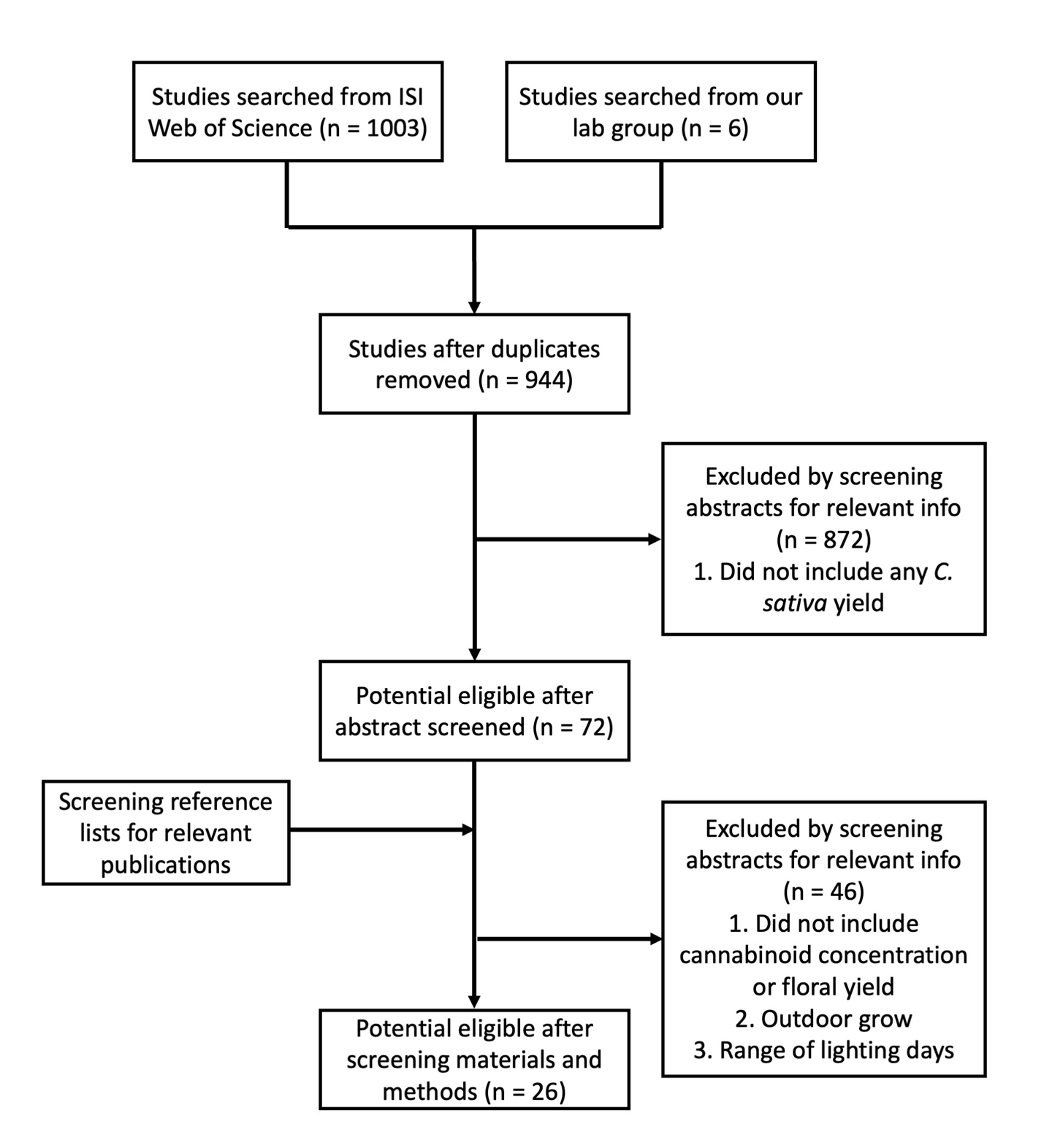


**Figure S1**. A workflow diagram showing the procedure for identifying, screening, and selecting publications for inclusion.

**Table S1**. Summary of studies (n=1003) found via searching key terms on ISI Web of Science database search on February 2, 2021.

| Search term | Result number | Study | |
| --- | --- | --- | --- |
| Cannabis photoperiod | 1 | Amaducci, S., Colauzzi, M., Bellocchi, G., Cosentino, S. L., Pahkala, K., Stomph, T. J., et al. (2012). Evaluation of a phenological model for strategic decisions for hemp (Cannabis Sativa L.) biomass production across European sites. *Ind. Crops Prod.* 37, 100–110. doi:10.1016/j.indcrop.2011.11.012. |  |
| Cannabis photoperiod | 2 | Amaducci, S., Colauzzi, M., Bellocchi, G., and Venturi, G. (2008). Modelling post-emergent hemp phenology (Cannabis sativa L.): Theory and evaluation. *Eur. J. Agron.* 28, 90–102. doi:10.1016/j.eja.2007.05.006. |  |
| Cannabis photoperiod | 3 | ARNOUX, M. (1966). INFLUENCE OF ENVIRONMENT ON SEXUAL EXPRESSION IN MONOECIOUS HEMP (CANNABIS SATIVA L) .3. NOTE ON EFFECTS OF PHOTOPERIOD AND NITROGEN SUPPLY. *Ann. L Amelior. DES PLANTES* 16, 259-\&. |  |
| Cannabis photoperiod | 4 | Backer, R., Schwinghamer, T., Rosenbaum, P., McCarty, V., Eichhorn Bilodeau, S., Lyu, D., et al. (2019). Closing the yield gap for cannabis: A meta-analysis of factors determining cannabis yield. *Front. Plant Sci.* 10, 495. doi:10.3389/fpls.2019.00495. |  |
| Cannabis photoperiod | 5 | Bauerle, W. L., McCullough, C., Iversen, M., and Hazlett, M. (2020). Leaf Age and Position Effects on Quantum Yield and Photosynthetic Capacity in Hemp Crowns. *PLANTS-BASEL* 9. doi:10.3390/plants9020271. |  |
| Cannabis photoperiod | 6 | Bilodeau, S. E., Wu, B.-S., Rufyikiri, A.-S., MacPherson, S., and Lefsrud, M. (2019). An Update on Plant Photobiology and Implications for Cannabis Production. *Front. Plant Sci.* 10. doi:10.3389/fpls.2019.00296. |  |
| Cannabis photoperiod | 7 | Cosentino, S. L., Testa, G., Scordia, D., and Copani, V. (2012). Sowing time and prediction of flowering of different hemp (Cannabis sativa L.) genotypes in southern Europe. *Ind. Crops Prod.* 37, 20–33. |  |
| Cannabis photoperiod | 8 | Faux, A.-M., Berhin, A., Dauguet, N., and Bertin, P. (2014). Sex chromosomes and quantitative sex expression in monoecious hemp (Cannabis sativa L.). *EUPHYTICA* 196, 183–197. doi:10.1007/s10681-013-1023-y. |  |
| Cannabis photoperiod | 9 | Faux, A.-M., Draye, X., Lambert, R., d’Andrimont, R., Raulier, P., and Bertin, P. (2013). The relationship of stem and seed yields to flowering phenology and sex expression in monoecious hemp (Cannabis sativa L.). *Eur. J. Agron.* 47, 11–22. doi:10.1016/j.eja.2013.01.006. |  |
| Cannabis photoperiod | 10 | Folina, A., Roussis, I., Kouneli, V., Kakabouki, J., Karidogianni, S., and Bilalis, D. (2019). OPPORTUNITIES FOR CULTIVATION OF MEDICAL CANNABIS (Cannabis saliva L.) IN GREECE. *Sci. Pap. A-AGRONOMY* 62, 293–300. |  |
| Cannabis photoperiod | 11 | Guerriero, G., Mangeot-Peter, L., Legay, S., Behr, M., Lutts, S., Siddiqui, K. S., et al. (2017). Identification of fasciclin-like arabinogalactan proteins in textile hemp (Cannabis sativa L.): in silico analyses and gene expression patterns in different tissues. *BMC Genomics* 18. doi:10.1186/s12864-017-3970-5. |  |
| Cannabis photoperiod | 12 | Hall, J., Bhattarai, S. P., and Midmore, D. J. (2012). Review of Flowering Control in Industrial Hemp. *J. Nat. FIBERS* 9, 23–36. doi:10.1080/15440478.2012.651848. |  |
| Cannabis photoperiod | 13 | Hall, J., Bhattarai, S. P., and Midmore, D. J. (2013). The Effects of Different Sowing Times on Maturity Rates, Biomass, and Plant Growth of Industrial Fiber Hemp. *J. Nat. FIBERS* 10, 40–50. doi:10.1080/15440478.2012.756639. |  |
| Cannabis photoperiod | 14 | Hall, J., Bhattarai, S. P., and Midmore, D. J. (2014). The Effects of Photoperiod on Phenological Development and Yields of Industrial Hemp. *J. Nat. FIBERS* 11, 87–106. doi:10.1080/15440478.2013.846840. |  |
| Cannabis photoperiod | 15 | Lata, H., Chandra, S., Mehmedic, Z., Khan, I. A., and ElSohly, M. A. (2012). In vitro germplasm conservation of high Delta(9)-tetrahydrocannabinol yielding elite clones of Cannabis sativa L. under slow growth conditions. *ACTA Physiol. Plant.* 34, 743–750. doi:10.1007/s11738-011-0874-x. |  |
| Cannabis photoperiod | 16 | Lisson, S. N., and Mendham, N. J. (2000). Cultivar, sowing date and plant density studies of fibre hemp (Cannabis sativa L.) in Tasmania. *Aust. J. Exp. Agric.* 40, 975–986. doi:10.1071/EA99130. |  |
| Cannabis photoperiod | 17 | Lisson, S. N., Mendham, N. J., and Carberry, P. S. (2000). Development of a hemp (Cannabis sativa L.) simulation model 2. The flowering response of two hemp cultivars to photoperiod. *Aust. J. Exp. Agric.* 40, 413–417. doi:10.1071/EA99059. |  |
| Cannabis photoperiod | 18 | Moher, M., Jones, M., and Zheng, Y. (2021). Photoperiodic Response of In Vitro Cannabis sativa Plants. *HORTSCIENCE* 56, 108–113. doi:10.21273/HORTSCI15452-20. |  |
| Cannabis photoperiod | 19 | Rich, L. N., Ferguson, E., Baker, A. D., and Chappell, E. (2020). A review of the potential impacts of artificial lights on fish and wildlife and how this may apply to cannabis cultivation. *Calif. FISH GAME* 106, 75–91. |  |
| Cannabis photoperiod | 20 | Salentijn, E. M. J., Petit, J., and Trindade, L. M. (2019). The Complex Interactions Between Flowering Behavior and Fiber Quality in Hemp. *Front. Plant Sci.* 10. doi:10.3389/fpls.2019.00614. |  |
| Cannabis photoperiod | 21 | Saloner, A., and Bernstein, N. (2020). Response of Medical Cannabis (Cannabis sativa L.) to Nitrogen Supply Under Long Photoperiod. *Front. Plant Sci.* 11. doi:10.3389/fpls.2020.572293. |  |
| Cannabis photoperiod | 22 | Saloner, A., Sacks, M. M., and Bernstein, N. (2019). Response of Medical Cannabis (Cannabis sativa L.) Genotypes to K Supply Under Long Photoperiod. *Front. Plant Sci.* 10. doi:10.3389/fpls.2019.01369. |  |
| Cannabis photoperiod | 23 | Spitzer-Rimon, B., Duchin, S., Bernstein, N., and Kamenetsky, R. (2019). Architecture and Florogenesis in Female Cannabis sativa Plants. *Front. Plant Sci.* 10. doi:10.3389/fpls.2019.00350. |  |
| Cannabis photoperiod | 24 | Tang, K., Struik, P. C., Yin, X., Thouminot, C., Bjelkova, M., Stramkale, V., et al. (2016). Comparing hemp (Cannabis sativa L.) cultivars for dual-purpose production under contrasting environments. *Ind. Crops Prod.* 87, 33–44. doi:10.1016/j.indcrop.2016.04.026. |  |
| Cannabis photoperiod | 25 | Techen, N., Chandra, S., Lata, H., ElSohly, M. A., and Khan, I. A. (2010). Genetic Identification of Female Cannabis sativa Plants at Early Developmental Stage. *Planta Med.* 76, 1938–1939. doi:10.1055/s-0030-1249978. |  |
| Cannabis photoperiod | 26 | Tipparat, P., Natakankitkul, S., Chamnivikaipong, P., and Chutiwat, S. (2012). Characteristics of cannabinoids composition of Cannabis plants grown in Northern Thailand and its forensic application. *FORENSIC Sci. Int.* 215, 164–170. doi:10.1016/j.forsciint.2011.05.006. |  |
| Cannabis photoperiod | 27 | Tipple, B. J., Hambach, B., Barnette, J. E., Chesson, L. A., and Ehleringer, J. R. (2016). The influences of cultivation setting on inflorescence lipid distributions, concentrations, and carbon isotope ratios of Cannabis sp. *FORENSIC Sci. Int.* 262, 233–241. doi:10.1016/j.forsciint.2016.03.029. |  |
| Cannabis photoperiod | 28 | Zanetti, F., Monti, A., and Berti, M. T. (2013). Challenges and opportunities for new industrial oilseed crops in EU-27: A review. *Ind. Crops Prod.* 50, 580–595. doi:10.1016/j.indcrop.2013.08.030. |  |
| Cannabis flowering | 29 | Abdollahi, M., Sefidkon, F., Calagari, M., Mousavi, A., and Mahomoodally, M. F. (2020). Impact of four hemp (Cannabis sativa L.) varieties and stage of plant growth on yield and composition of essential oils. *Ind. Crops Prod.* 155. doi:10.1016/j.indcrop.2020.112793. |  |
| Cannabis flowering | 30 | Aboulaich, N., Mar Trigo, M., Bouziane, H., Cabezudo, B., Recio, M., El Kadiri, M., et al. (2013). Variations and origin of the atmospheric pollen of Cannabis detected in the province of Tetouan (NW Morocco): 2008-2010. *Sci. Total Environ.* 443, 413–419. doi:10.1016/j.scitotenv.2012.10.075. |  |
| Cannabis flowering | 31 | Adal, A. M., Doshi, K., Holbrook, L., and Mahmoud, S. S. (2021). Comparative RNA-Seq analysis reveals genes associated with masculinization in female Cannabis sativa. *Planta* 253. doi:10.1007/s00425-020-03522-y. |  |
| Cannabis flowering | 32 | Aggarwal, S. K., Carter, G. T., Zumbrunnen, C., Morrill, R., Sullivan, M., and Mayer, J. D. (2013). From 32 Ounces to Zero: A Medical Geographic Study of Dispensing a Cultivated Batch of ``Plum{’’} Cannabis Flowers to Medical Marijuana Patients in Washington State. *J. Psychoactive Drugs* 45, 141–155. doi:10.1080/02791072.2013.785835. |  |
| Cannabis flowering | 33 | Al-Zouabi, I., Stogner, J. M., Miller, B. L., and Lane, E. S. (2018). Butane hash oil and dabbing: insights into use, amateur production techniques, and potential harm mitigation. *Subst. Abuse Rehabil.* 9, 91–101. doi:10.2147/SAR.S135252. |  |
| Cannabis flowering | 34 | Al Madini, A. M., Sassine, Y. N., El-Ganainy, S. M., Hourani, W., and El Sebaaly, Z. (2019). COMPARATIVE STUDY ON PHENOLOGY, YIELD AND QUALITY OF IRANIAN SAFFRON CULTIVATED IN LEBANON AND IRAN. *FRESENIUS Environ. Bull.* 28, 9655–9660. |  |
| Cannabis flowering | 35 | Allegrone, G., Razzano, F., Pollastro, F., and Grassi, G. (2019). Determination of melatonin content of different varieties of hemp (Cannabis sativa L.) by liquid chromatography tandem mass spectrometry. *SN Appl. Sci.* 1. doi:10.1007/s42452-019-0759-y. |  |
| Cannabis flowering | 36 | Amaducci, S., Colauzzi, M., Bellocchi, G., Cosentino, S. L., Pahkala, K., Stomph, T. J., et al. (2012). Evaluation of a phenological model for strategic decisions for hemp (Cannabis Sativa L.) biomass production across European sites. *Ind. Crops Prod.* 37, 100–110. doi:10.1016/j.indcrop.2011.11.012. |  |
| Cannabis flowering | 37 | Amaducci, S., Colauzzi, M., Bellocchi, G., and Venturi, G. (2008a). Modelling post-emergent hemp phenology (Cannabis sativa L.): Theory and evaluation. *Eur. J. Agron.* 28, 90–102. doi:10.1016/j.eja.2007.05.006. |  |
| Cannabis flowering | 38 | Amaducci, S., Muessig, J., Zatta, A., and Pelatti, F. (2007). HEMP SYS: Design, development and up-scaling of a sustainable production system for HEMP textiles: An integrated quality systems approach. How to affect hemp fiber quality? in *TEXTILES FOR SUSTAINABLE DEVELOPMENT*, ed. Anandjiwala, R and Hunter, L and Kozlowski, R and Zaikov, G, 73+. |  |
| Cannabis flowering | 39 | Amaducci, S., Zatta, A., Pelatti, F., and Venturi, G. (2008b). Influence of agronomic factors on yield and quality of hemp (Cannabis sativa L.) fibre and implication for an innovative production system. *F. Crop. Res.* 107, 161–169. doi:10.1016/j.fcr.2008.02.002. |  |
| Cannabis flowering | 40 | Anderson, S. A., Weiblen, G., and Speckhard, D. (2013). FT flowering gene examination in Cannabis sativa. *Abstr. Pap. Am. Chem. Soc.* 245. |  |
| Cannabis flowering | 41 | Andre, A., Leupin, M., Kneubuhl, M., Pedan, V., and Chetschik, I. (2020). Evolution of the Polyphenol and Terpene Content, Antioxidant Activity and Plant Morphology of Eight Different Fiber-Type Cultivars of Cannabis sativa L. Cultivated at Three Sowing Densities. *PLANTS-BASEL* 9. doi:10.3390/plants9121740. |  |
| Cannabis flowering | 42 | Arnold, J. C., Nation, T., and McGregor, I. S. (2020). Prescribing medicinal cannabis. *Aust. Prescr.* 43, 152–159. doi:10.18773/austprescr.2020.052. |  |
| Cannabis flowering | 43 | Ascrizzi, R., Iannone, M., Cinque, G., Marianelli, A., Pistelli, L., and Flamini, G. (2020). ``Hemping{’’} the drinks: Aromatizing alcoholic beverages with a blend of Cannabis sativa L. flowers. *FOOD Chem.* 325. doi:10.1016/j.foodchem.2020.126909. |  |
| Cannabis flowering | 44 | Atapattu, S. N., and Johnson, K. R. D. (2020). Pesticide analysis in cannabis products. *J. Chromatogr. A* 1612. doi:10.1016/j.chroma.2019.460656. |  |
| Cannabis flowering | 45 | Babaei, M., and Ajdanian, L. (2020). Screening of different Iranian ecotypes of cannabis under water deficit stress. *Sci. Hortic. (Amsterdam).* 260. doi:10.1016/j.scienta.2019.108904. |  |
| Cannabis flowering | 46 | Backer, R., Mandolino, G., Wilkins, O., ElSohly, M. A., and Smith, D. L. (2020). Editorial: Cannabis Genomics, Breeding and Production. *Front. Plant Sci.* 11. doi:10.3389/fpls.2020.591445. |  |
| Cannabis flowering | 47 | Baldini, M., Ferfuia, C., Piani, B., Sepulcri, A., Dorigo, G., Zuliani, F., et al. (2018). The Performance and Potentiality of Monoecious Hemp (Cannabis sativa L.) Cultivars as a Multipurpose Crop. *AGRONOMY-BASEL* 8. doi:10.3390/agronomy8090162. |  |
| Cannabis flowering | 48 | Barcaccia, G., Palumbo, F., Scariolo, F., Vannozzi, A., Borin, M., and Bona, S. (2020). Potentials and Challenges of Genomics for Breeding Cannabis Cultivars. *Front. Plant Sci.* 11. doi:10.3389/fpls.2020.573299. |  |
| Cannabis flowering | 49 | Barnett, S. E., Cala, A. R., Hansen, J. L., Crawford, J., Viands, D. R., Smart, L. B., et al. (2020). Evaluating the Microbiome of Hemp. *PHYTOBIOMES J.* 4, 351–363. doi:10.1094/PBIOMES-06-20-0046-R. |  |
| Cannabis flowering | 50 | Bennett, S. J., Snell, R., and Wright, D. (2006). Effect of variety, seed rate and time of cutting on fibre yield of dew-retted hemp. *Ind. Crops Prod.* 24, 79–86. doi:10.1016/j.indcrop.2006.03.007. |  |
| Cannabis flowering | 51 | Bernstein, N., Gorelick, J., and Koch, S. (2019a). Interplay between chemistry and morphology in medical cannabis (Cannabis sativa L.). *Ind. Crops Prod.* 129, 185–194. doi:10.1016/j.indcrop.2018.11.039. |  |
| Cannabis flowering | 52 | Bernstein, N., Gorelick, J., Zerahia, R., and Koch, S. (2019b). Impact of N, P, K, and Humic Acid Supplementation on the Chemical Profile of Medical Cannabis (Cannabis sativa L). *Front. Plant Sci.* 10. doi:10.3389/fpls.2019.00736. |  |
| Cannabis flowering | 53 | Bidwell, L. C., Ellingson, J. M., Karoly, H. C., YorkWilliams, S. L., Hitchcock, L. N., Tracy, B. L., et al. (2020). Association of Naturalistic Administration of Cannabis Flower and Concentrates With Intoxication and Impairment. *JAMA PSYCHIATRY* 77, 787–796. doi:10.1001/jamapsychiatry.2020.0927. |  |
| Cannabis flowering | 54 | Bidwell, L. C., YorkWilliams, S., Bryan, A., Klawitter, J., Galinkin, J., Sempio, C., et al. (2017). An Observational Study of the Acute Effects of Legal Market Smoked Flower and Concentrated Cannabis. *NEUROPSYCHOPHARMACOLOGY* 42, S624–S625. |  |
| Cannabis flowering | 55 | Bienkowski, A. O., and Orlova-Bienkowskaja, M. J. (2015). Trophic specialization of leaf beetles (Coleoptera, Chrysomelidae) in the Volga Upland. *Biol. Bull.* 42, 863–869. doi:10.1134/S1062359015100015. |  |
| Cannabis flowering | 56 | Bilodeau, S. E., Wu, B.-S., Rufyikiri, A.-S., MacPherson, S., and Lefsrud, M. (2019). An Update on Plant Photobiology and Implications for Cannabis Production. *Front. Plant Sci.* 10. doi:10.3389/fpls.2019.00296. |  |
| Cannabis flowering | 57 | Blake, A., and Nahtigal, I. (2019). The evolving landscape of cannabis edibles. *Curr. Opin. FOOD Sci.* 28, 25–31. doi:10.1016/j.cofs.2019.03.009. |  |
| Cannabis flowering | 58 | Blebea, N. M., Costache, T., and Negres, S. (2019). THE QUALITATIVE AND QUANTITATIVE ANALYSIS OF CBD IN HEMP OILS BY UHPLC WITH PDA AND APPLICATIONS. *Sci. Pap. D-ANIMAL Sci.* 62, 138–142. |  |
| Cannabis flowering | 59 | Bleuze, L., Chabbert, B., Lashermes, G., and Recous, S. (2020). Hemp harvest time impacts on the dynamics of microbial colonization and hemp stems degradation during dew retting. *Ind. Crops Prod.* 145. doi:10.1016/j.indcrop.2020.112122. |  |
| Cannabis flowering | 60 | Booth, J. K., Yuen, M. M. S., Jancsik, S., Madilao, L. L., Page, J. E., and Bohlmann, J. (2020). Terpene Synthases and Terpene Variation in Cannabis sativa. *PLANT Physiol.* 184, 130–147. doi:10.1104/pp.20.00593. |  |
| Cannabis flowering | 61 | Borgelt, L. M., Franson, K. L., Nussbaum, A. M., and Wang, G. S. (2013). The Pharmacologic and Clinical Effects of Medical Cannabis. *Pharmacotherapy* 33, 195–209. doi:10.1002/phar.1187. |  |
| Cannabis flowering | 62 | Braich, S., Baillie, R. C., Jewell, L. S., Spangenberg, G. C., and Cogan, N. O. I. (2019). Generation of a Comprehensive Transcriptome Atlas and Transcriptome Dynamics in Medicinal Cannabis. *Sci. Rep.* 9. doi:10.1038/s41598-019-53023-6. |  |
| Cannabis flowering | 63 | Broseus, J., Anglada, F., and Esseiva, P. (2010). The differentiation of fibre- and drug type Cannabis seedlings by gas chromatography/mass spectrometry and chemometric tools. *FORENSIC Sci. Int.* 200, 87–92. doi:10.1016/j.forsciint.2010.03.034. |  |
| Cannabis flowering | 64 | Bruce, D. M., Hobson, R. N., White, R. P., and Hobson, J. (2001). Stripping of leaves and flower heads to improve the harvesting of fibre hemp. *J. Agric. Eng. Res.* 78, 43–50. doi:10.1006/jaer.2000.0632. |  |
| Cannabis flowering | 65 | Bruci, Z., Papoutsis, I., Athanaselis, S., Nikolaou, P., Pazari, E., Spiliopoulou, C., et al. (2012). First systematic evaluation of the potency of Cannabis sativa plants grown in Albania. *FORENSIC Sci. Int.* 222, 40–46. doi:10.1016/j.forsciint.2012.04.032. |  |
| Cannabis flowering | 66 | Burgel, L., Hartung, J., and Graeff-Hoenninger, S. (2020a). Impact of Different Growing Substrates on Growth, Yield and Cannabinoid Content of Two Cannabis sativa L. Genotypes in a Pot Culture. *HORTICULTURAE* 6. doi:10.3390/horticulturae6040062. |  |
| Cannabis flowering | 67 | Burgel, L., Hartung, J., Pflugfelder, A., and Graeff-Hoenninger, S. (2020b). Impact of Growth Stage and Biomass Fractions on Cannabinoid Content and Yield of Different Hemp (Cannabis sativa L.) Genotypes. *AGRONOMY-BASEL* 10. doi:10.3390/agronomy10030372. |  |
| Cannabis flowering | 68 | Callaghan, R. C., Sanches, M., Benny, C., Stockwell, T., Sherk, A., and Kish, S. J. (2019). Who consumes most of the cannabis in Canada? Profiles of cannabis consumption by quantity. *Drug Alcohol Depend.* 205. doi:10.1016/j.drugalcdep.2019.107587. |  |
| Cannabis flowering | 69 | Calzolari, D., Magagnini, G., Lucini, L., Grassi, G., Appendino, G. B., and Amaducci, S. (2017). High added-value compounds from Cannabis threshing residues. *Ind. Crops Prod.* 108, 558–563. doi:10.1016/j.indcrop.2017.06.063. |  |
| Cannabis flowering | 70 | Campiglia, E., Radicetti, E., and Mancinelli, R. (2017). Plant density and nitrogen fertilization affect agronomic performance of industrial hemp (Cannabis sativa L.) in Mediterranean environment. *Ind. Crops Prod.* 100, 246–254. doi:10.1016/j.indcrop.2017.02.022. |  |
| Cannabis flowering | 71 | Caplan, D., Dixon, M., and Zheng, Y. (2017). Optimal Rate of Organic Fertilizer during the Flowering Stage for Cannabis Grown in Two Coir-based Substrates. *HORTSCIENCE* 52, 1796–1803. doi:10.21273/HORTSCI12401-17. |  |
| Cannabis flowering | 72 | Caplan, D., Dixon, M., and Zheng, Y. (2019). Increasing Inflorescence Dry Weight and Cannabinoid Content in Medical Cannabis Using Controlled Drought Stress. *HORTSCIENCE* 54, 964–969. doi:10.21273/HORTSCI13510-18. |  |
| Cannabis flowering | 73 | Caulkins, J. P., Bao, Y., Davenport, S., Fahli, I., Guo, Y., Kinnard, K., et al. (2018). Big data on a big new market: Insights from Washington State’s legal cannabis market. *Int. J. DRUG POLICY* 57, 86–94. doi:10.1016/j.drugpo.2018.03.031. |  |
| Cannabis flowering | 74 | Caulkins, J. P., Davenport, S., Doanvo, A., Furlong, K., Siddique, A., Turner, M., et al. (2019). Triangulating web & general population surveys: Do results match legal cannabis market sales? *Int. J. DRUG POLICY* 73, 293–300. doi:10.1016/j.drugpo.2019.06.010. |  |
| Cannabis flowering | 75 | Caulkins, J. P., Pardo, B., and Kilmer, B. (2020). Intensity of cannabis use: Findings from three online surveys. *Int. J. DRUG POLICY* 79. doi:10.1016/j.drugpo.2020.102740. |  |
| Cannabis flowering | 76 | Chandra, S., Lata, H., Mehmedic, Z., Khan, I. A., and ElSohly, M. A. (2015). Light dependence of photosynthesis and water vapor exchange characteristics in different high Delta(9)-THC yielding varieties of Cannabis sativa L. *J. Appl. Res. Med. Aromat. PLANTS* 2, 39–47. doi:10.1016/j.jarmap.2015.03.002. |  |
| Cannabis flowering | 77 | Chen, D.-J., Gao, M., Gao, F.-F., Su, Q.-X., and Wu, J. (2017). Brain cannabinoid receptor 2: expression, function and modulation. *ACTA Pharmacol. Sin.* 38, 312–316. doi:10.1038/aps.2016.149. |  |
| Cannabis flowering | 78 | Choi, S., Huang, B. C., and Gamaldo, C. E. (2020). Therapeutic Uses of Cannabis on Sleep Disorders and Related Conditions. *J. Clin. Neurophysiol.* 37, 39–49. doi:10.1097/WNP.0000000000000617. |  |
| Cannabis flowering | 79 | Choi, Y. H., Hazekamp, A., Peltenburg-Looman, A. M. G., Frederich, M., Erkelens, C., Lefeber, A. W. M., et al. (2004). NMR assignments of the major Cannabinoids and cannabiflavonoids isolated from flowers of Cannabis sativa. *Phytochem. Anal.* 15, 345–354. doi:10.1002/pca.787. |  |
| Cannabis flowering | 80 | Chung, T., Bae, S. W., Mun, E.-Y., Suffoletto, B., Nishiyama, Y., Jang, S., et al. (2020). Mobile Assessment of Acute Effects of Marijuana on Cognitive Functioning in Young Adults: Observational Study. *JMIR MHEALTH UHEALTH* 8. doi:10.2196/16240. |  |
| Cannabis flowering | 81 | Cifuentes-Torres, L., Mendoza-Espinosa, L. G., Correa-Reyes, G., and Daessle, L. W. Hydroponics with wastewater: a review of trends and opportunities. *WATER Environ. J.* doi:10.1111/wej.12617. |  |
| Cannabis flowering | 82 | Cockson, P., Schroeder-Moreno, M., Veazie, P., Barajas, G., Logan, D., Davis, M., et al. (2020). Impact of Phosphorus on Cannabis sativa Reproduction, Cannabinoids, and Terpenes. *Appl. Sci.* 10. doi:10.3390/app10217875. |  |
| Cannabis flowering | 83 | Contreras, A. E., Hall, K. E., Vigil, D. I., Rosenthal, A., Azofeifa, A., and Van Dyke, M. (2020). Results from the Colorado Cannabis Users Survey on Health (CUSH), 2016. *Int. J. Ment. Health Addict.* 18, 1–13. doi:10.1007/s11469-018-0047-x. |  |
| Cannabis flowering | 84 | Cosentino, S. L., Testa, G., Scordia, D., and Copani, V. (2012). Sowing time and prediction of flowering of different hemp (Cannabis sativa L.) genotypes in southern Europe. *Ind. Crops Prod.* 37, 20–33. |  |
| Cannabis flowering | 85 | Coyle, H. M., Shutler, G., Abrams, S., Hanniman, J., Neylon, S., Ladd, C., et al. (2003). A simple DNA extraction method for marijuana samples used in amplified fragment length polymorphism (AFLP) analysis. *J. Forensic Sci.* 48, 343–347. |  |
| Cannabis flowering | 86 | Cranshaw, W., Schreiner, M., Britt, K., Kuhar, T. P., McPartland, J., and Grant, J. (2019). Developing Insect Pest Management Systems for Hemp in the United States: A Work in Progress. *J. Integr. PEST Manag.* 10. doi:10.1093/jipm/pmz023. |  |
| Cannabis flowering | 87 | Craven, C. B., Wawryk, N., Jiang, P., Liu, Z., and Li, X.-F. (2019). Pesticides and trace elements in cannabis: Analytical and environmental challenges and opportunities. *J. Environ. Sci.* 85, 82–93. doi:10.1016/j.jes.2019.04.028. |  |
| Cannabis flowering | 88 | Cremer-Schaeffer, P., Knoess, W., and Broich, K. (2019a). Cannabis for medical purposes. Impact of the law of 6 March 2017 on physicians. *SCHMERZ* 33, 378–383. doi:10.1007/s00482-019-0387-x. |  |
| Cannabis flowering | 89 | Cremer-Schaeffer, P., Schmidt-Wolf, G., and Broich, K. (2019b). Cannabismedicines in pain management. Interim analysis of the survey accompanying the prescription of cannabis-basedmedicines in Germany with regard to pain as primarily treated symptom. *SCHMERZ* 33, 415–423. doi:10.1007/s00482-019-00399-z. |  |
| Cannabis flowering | 90 | Cuttler, C., Spradlin, A., Cleveland, M. J., and Craft, R. M. (2020). Short- and Long-Term Effects of Cannabis on Headache and Migraine. *J. PAIN* 21, 722–730. doi:10.1016/j.jpain.2019.11.001. |  |
| Cannabis flowering | 91 | Da Porto, C., Decorti, D., and Natolino, A. (2014). Separation of aroma compounds from industrial hemp inflorescences (Cannabis sativa L.) by supercritical CO2 extraction and on-line fractionation. *Ind. Crops Prod.* 58, 99–103. doi:10.1016/j.indcrop.2014.03.042. |  |
| Cannabis flowering | 92 | Daniulaityte, R., Lamy, F. R., Barratt, M., Nahhas, R. W., Martins, S. S., Boyer, E. W., et al. (2017). Characterizing marijuana concentrate users: A web-based survey. *Drug Alcohol Depend.* 178, 399–407. doi:10.1016/j.drugalcdep.2017.05.034. |  |
| Cannabis flowering | 93 | De Backer, B., Maebe, K., Verstraete, A. G., and Charlier, C. (2012). Evolution of the Content of THC and Other Major Cannabinoids in Drug-Type Cannabis Cuttings and Seedlings During Growth of Plants. *J. Forensic Sci.* 57, 918–922. doi:10.1111/j.1556-4029.2012.02068.x. |  |
| Cannabis flowering | 94 | Deguchi, M., Bogush, D., Weeden, H., Spuhler, Z., Potlakayala, S., Kondo, T., et al. (2020). Establishment and optimization of a hemp (Cannabis sativa L.) agroinfiltration system for gene expression and silencing studies. *Sci. Rep.* 10. doi:10.1038/s41598-020-60323-9. |  |
| Cannabis flowering | 95 | della Rocca, G., and Di Salvo, A. (2020). Hemp in Veterinary Medicine: From Feed to Drug. *Front. Vet. Sci.* 7. doi:10.3389/fvets.2020.00387. |  |
| Cannabis flowering | 96 | DEMEIJER, E. P. M., and KEIZER, L. C. P. (1994). VARIATION OF CANNABIS FOR PHENOLOGICAL DEVELOPMENT AND STEM ELONGATION IN RELATION TO STEM PRODUCTION. *F. Crop. Res.* 38, 37–46. doi:10.1016/0378-4290(94)90030-2. |  |
| Cannabis flowering | 97 | DiMatteo, J., Kurtz, L., and Lubell-Brand, J. D. (2020). Pollen Appearance and In Vitro Germination Varies for Five Strains of Female Hemp Masculinized Using Silver Thiosulfate. *HORTSCIENCE* 55, 547–549. doi:10.21273/HORTSCI14842-20. |  |
| Cannabis flowering | 98 | dos Santos, N. A., Ortiz, R. S., Limberger, R. P., Lacerda Junior, V., and Romao, W. (2018). Identification of Delta(9)-Tetrahydrocannabinol (Delta(9)-THC) in Cannabis Seeds by Electrospray Ionization Fourier Transform Ion Cyclotron Resonance Mass Spectrometry (ESI(-)FT-ICR MS). *ORBITAL-THE Electron. J. Chem.* 10, 381–385. doi:10.17807/orbital.v10i5.1149. |  |
| Cannabis flowering | 99 | dos Santos, N. A., Tose V, L., da Silva, S. R. C., Murgu, M., Kuster, R. M., Ortiz, R. S., et al. (2019). Analysis of Isomeric Cannabinoid Standards and Cannabis Products by UPLC-ESI-TWIM-MS: a Comparison with GC-MS and GC x GC-QMS. *J. Braz. Chem. Soc.* 30, 60–70. doi:10.21577/0103-5053.20180152. |  |
| Cannabis flowering | 100 | Esposito, L. G. A., Overbaugh, E., Xiong, J., Rathinasabapathy, T., Komarnytsky, S., and Esposito, D. A. (2019). Comparative Anti-inflammatory Activity of Whole Root, Stem, Leaf, and Flower Extracts from Female and Male CBD Hemp (Cannabis sativa L.) Plants. *Vitr. Cell. Dev. Biol.* 55, S72–S73. |  |
| Cannabis flowering | 101 | Faux, A.-M., Berhin, A., Dauguet, N., and Bertin, P. (2014). Sex chromosomes and quantitative sex expression in monoecious hemp (Cannabis sativa L.). *EUPHYTICA* 196, 183–197. doi:10.1007/s10681-013-1023-y. |  |
| Cannabis flowering | 102 | Faux, A.-M., and Bertin, P. (2014). Modelling approach for the quantitative variation of sex expression in monoecious hemp (Cannabis sativa L.). *PLANT Breed.* 133, 782–787. doi:10.1111/pbr.12208. |  |
| Cannabis flowering | 103 | Faux, A.-M., Draye, X., Flamand, M.-C., Occre, A., and Bertin, P. (2016). Identification of QTLs for sex expression in dioecious and monoecious hemp (Cannabis sativa L.). *EUPHYTICA* 209, 357–376. doi:10.1007/s10681-016-1641-2. |  |
| Cannabis flowering | 104 | Faux, A.-M., Draye, X., Lambert, R., d’Andrimont, R., Raulier, P., and Bertin, P. (2013). The relationship of stem and seed yields to flowering phenology and sex expression in monoecious hemp (Cannabis sativa L.). *Eur. J. Agron.* 47, 11–22. doi:10.1016/j.eja.2013.01.006. |  |
| Cannabis flowering | 105 | Feingold, D., Brill, S., Goor-Aryeh, I., Delayahu, Y., and Lev-Ran, S. (2020). Depression level, not pain severity, is associated with smoked medical marijuana dosage among chronic pain patients. *J. Psychosom. Res.* 135. doi:10.1016/j.jpsychores.2020.110130. |  |
| Cannabis flowering | 106 | Ferrante, C., Recinella, L., Ronci, M., Menghini, L., Brunetti, L., Chiavaroli, A., et al. (2019). Multiple pharmacognostic characterization on hemp commercial cultivars: Focus on inflorescence water extract activity. *FOOD Chem. Toxicol.* 125, 452–461. doi:10.1016/j.fct.2019.01.035. |  |
| Cannabis flowering | 107 | Fitzgerald, K. T., Bronstein, A. C., and Newquist, K. L. (2013). Marijuana Poisoning. *Top. Companion Anim. Med.* 28, 8–12. doi:10.1053/j.tcam.2013.03.004. |  |
| Cannabis flowering | 108 | Flicker, N. R., Poveda, K., and Grab, H. (2020). The Bee Community of Cannabis sativa and Corresponding Effects of Landscape Composition. *Environ. Entomol.* 49, 197–202. doi:10.1093/ee/nvz141. |  |
| Cannabis flowering | 109 | Gagne, S. J., Stout, J. M., Liu, E., Boubakir, Z., Clark, S. M., and Page, J. E. (2012). Identification of olivetolic acid cyclase from Cannabis sativa reveals a unique catalytic route to plant polyketides. *Proc. Natl. Acad. Sci. U. S. A.* 109, 12811–12816. doi:10.1073/pnas.1200330109. |  |
| Cannabis flowering | 110 | Garbacik, E. T., Korai, R. P., Frater, E. H., Korterik, J. P., Otto, C., and Offerhaus, H. L. (2013). In planta imaging of Delta(9)-tetrahydrocannabinolic acid in Cannabis sativa L. with hyperspectral coherent anti-Stokes Raman scattering microscopy. *J. Biomed. Opt.* 18. doi:10.1117/1.JBO.18.4.046009. |  |
| Cannabis flowering | 111 | Garcia-Tejero, I. F., Duran-Zuazo, V. H., Perez-Alvarez, R., Hernandez, A., Casano, S., Moron, M., et al. (2014). Impact of Plant Density and Irrigation on Yield of Hemp (Cannabis sativa L.) in a Mediterranean Semi-arid Environment. *J. Agric. Sci. Technol.* 16, 887–895. |  |
| Cannabis flowering | 112 | Garcia-Tejero, I. F., Duran Zuazo, V. H., Sanchez-Carnenero, C., Hernandez, A., Ferreiro-Vera, C., and Casand, S. (2019). Seeking suitable agronomical practices for industrial hemp (Cannabis sativa L.) cultivation for biomedical applications. *Ind. Crops Prod.* 139. doi:10.1016/j.indcrop.2019.111524. |  |
| Cannabis flowering | 113 | Garfinkel, A. R. (2020). Three Botrylis Species Found Causing Gray Mold on Industrial Hemp (Cannabis saliva) in Oregon. *PLANT Dis.* 104, 2026. doi:10.1094/PDIS-01-20-0055-PDN. |  |
| Cannabis flowering | 114 | Gilbert, A. N., and DiVerdi, J. A. (2018). Consumer perceptions of strain differences in Cannabis aroma. *PLoS One* 13. doi:10.1371/journal.pone.0192247. |  |
| Cannabis flowering | 115 | Gilbert, A. N., and DiVerdi, J. A. (2019). Use of rating scales versus check-all-that-apply ballots in quantifying strain-specific Cannabis aroma. *J. Sens. Stud.* 34. doi:10.1111/joss.12499. |  |
| Cannabis flowering | 116 | Gilbert, A. N., and DiVerdi, J. A. (2020). Human olfactory detection of packaged cannabis. *Sci. JUSTICE* 60, 169–172. doi:10.1016/j.scijus.2019.10.007. |  |
| Cannabis flowering | 117 | Giroud, C., de Cesare, M., Berthet, A., Varlet, V., Concha-Lozano, N., and Favrat, B. (2015). E-Cigarettes: A Review of New Trends in Cannabis Use. *Int. J. Environ. Res. Public Health* 12, 9988–10008. doi:10.3390/ijerph120809988. |  |
| Cannabis flowering | 118 | Gloss, D. (2015). An Overview of Products and Bias in Research. *NEUROTHERAPEUTICS* 12, 731–734. doi:10.1007/s13311-015-0370-x. |  |
| Cannabis flowering | 119 | Godlaski, T. M. (2012). Shiva, Lord of Bhang. *Subst. Use Misuse* 47, 1067–1072. doi:10.3109/10826084.2012.684308. |  |
| Cannabis flowering | 120 | Goldstein, R. S., Sumner, D. A., and Fafard, A. (2019). Retail cannabis prices in California through legalization, regulation and taxation. *Calif. Agric.* 73, 136–145. doi:10.3733/ca.2019a0025. |  |
| Cannabis flowering | 121 | Goodman, S., Wadsworth, E., Schauer, G., and Hammond, D. Use and Perceptions of Cannabidiol Products in Canada and in the United States. *CANNABIS CANNABINOID Res.* doi:10.1089/can.2020.0093. |  |
| Cannabis flowering | 122 | Grijo, D. R., Bidoia, D. L., Nakamura, C. V., Osorio, I. V., and Cardozo-Filho, L. (2019). Analysis of the antitumor activity of bioactive compounds of Cannabis flowers extracted by green solvents. *J. Supercrit. Fluids* 149, 20–25. doi:10.1016/j.supflu.2019.03.012. |  |
| Cannabis flowering | 123 | Grijo, D. R., Bidoia, D. L., Nakamura, C. V, Vieitez, I., and Cardozo-Filho, L. (2020). Analysis of the Antitumor Activity of Bioactive Compounds of Cannabis Flowers Extracted by Green Solvents. *J. Am. OIL Chem. Soc.* 97, 57. |  |
| Cannabis flowering | 124 | Grijo, D. R., Vieitez Osorio, I. A., and Cardozo-Filho, L. (2018). Supercritical extraction strategies using CO2 and ethanol to obtain cannabinoid compounds from Cannabis hybrid flowers. *J. CO2 Util.* 28, 174–180. doi:10.1016/j.jcou.2018.09.022. |  |
| Cannabis flowering | 125 | Grof, C. P. L. (2018). Cannabis, from plant to pill. *Br. J. Clin. Pharmacol.* 84, 2463–2467. doi:10.1111/bcp.13618. |  |
| Cannabis flowering | 126 | Grotenhermen, F. (2018). The German medical cannabis law of 2017. *DRUGS AND ALCOHOL TODAY* 18, 117–122. doi:10.1108/DAT-03-2018-0012. |  |
| Cannabis flowering | 127 | Grotenhermen, F., and Mueller-Vahl, K. (2012). The Therapeutic Potential of Cannabis and Cannabinoids. *Dtsch. Arztebl. Int.* 109, 495-U39. doi:10.3238/arztebl.2012.0495. |  |
| Cannabis flowering | 128 | Grotenhermen, F., and Mueller-Vahl, K. (2016a). Cannabis and Cannabinoids in Medicine: Facts and Perspective. *SUCHTTHERAPIE* 17, 71–76. doi:10.1055/s-0042-100702. |  |
| Cannabis flowering | 129 | Grotenhermen, F., and Mueller-Vahl, K. (2016b). Medicinal Uses of Marijuana and Cannabinoids. *CRC. Crit. Rev. Plant Sci.* 35, 378–405. doi:10.1080/07352689.2016.1265360. |  |
| Cannabis flowering | 130 | Guerriero, G., Mangeot-Peter, L., Legay, S., Behr, M., Lutts, S., Siddiqui, K. S., et al. (2017). Identification of fasciclin-like arabinogalactan proteins in textile hemp (Cannabis sativa L.): in silico analyses and gene expression patterns in different tissues. *BMC Genomics* 18. doi:10.1186/s12864-017-3970-5. |  |
| Cannabis flowering | 131 | Gulck, T., and Moller, B. L. (2020). Phytocannabinoids: Origins and Biosynthesis. *Trends Plant Sci.* 25, 985–1004. doi:10.1016/j.tplants.2020.05.005. |  |
| Cannabis flowering | 132 | Gupta, A. K., Jain, A., Roy, P., and Singh, R. (2020). Pharmacological Evaluation of Cannabis indica For Their Aphrodisiac Potential. *Int. J. AYURVEDIC Med.* 11, 399–404. |  |
| Cannabis flowering | 133 | Gur, M., Guder, A., Verep, D., Guney, K., Ozkan, O. E., Seki, N., et al. (2018). Some Important Plants for Epilepsy Treatment: Antioxidant Activity and Flavonoid Compositions. *Iran. J. Sci. Technol. Trans. A-SCIENCE* 42, 1847–1857. doi:10.1007/s40995-017-0361-3. |  |
| Cannabis flowering | 134 | Haedener, M., Vieten, S., Weinmann, W., and Mahler, H. (2019). A preliminary investigation of lung availability of cannabinoids by smoking marijuana or dabbing BHO and decarboxylation rate of THC- and CBD-acids. *FORENSIC Sci. Int.* 295, 207–212. doi:10.1016/j.forsciint.2018.12.021. |  |
| Cannabis flowering | 135 | Haeuser, W., Hoch, E., Petzke, F., Thomasius, R., Radbruch, L., Batra, A., et al. (2019). Medicinal cannabis and cannabis-basedmedication: an appeal to physicians, journalists, health insurances, and politicians for their responsible handling. *SCHMERZ* 33, 466–470. doi:10.1007/s00482-019-00409-0. |  |
| Cannabis flowering | 136 | Hall, J., Bhattarai, S. P., and Midmore, D. J. (2012). Review of Flowering Control in Industrial Hemp. *J. Nat. FIBERS* 9, 23–36. doi:10.1080/15440478.2012.651848. |  |
| Cannabis flowering | 137 | Hall, J., Bhattarai, S. P., and Midmore, D. J. (2013). The Effects of Different Sowing Times on Maturity Rates, Biomass, and Plant Growth of Industrial Fiber Hemp. *J. Nat. FIBERS* 10, 40–50. doi:10.1080/15440478.2012.756639. |  |
| Cannabis flowering | 138 | Hall, J., Bhattarai, S. P., and Midmore, D. J. (2014). The Effects of Photoperiod on Phenological Development and Yields of Industrial Hemp. *J. Nat. FIBERS* 11, 87–106. doi:10.1080/15440478.2013.846840. |  |
| Cannabis flowering | 139 | HANUS, L. (1978). CONTRIBUTION TO THE CONTENT OF THE PSYCHOTOMIMETICALLY ACTIVE DELTA-9-TETRAHYDROCANNABINOL IN FEMALE (MARIHUANA) AND MALE FLOWERING TOPS OF CANNABIS-SATIVA L CULTIVATED IN CLIMATIC CONDITIONS OF CZECHOSLOVAKIA. *Act. Nerv. Super. (Praha).* 20, 282–284. |  |
| Cannabis flowering | 140 | Happyana, N., Agnolet, S., Muntendam, R., Van Dam, A., Schneider, B., and Kayser, O. (2013). Analysis of cannabinoids in laser-microdissected trichomes of medicinal Cannabis sativa using LCMS and cryogenic NMR. *Phytochemistry* 87, 51–59. doi:10.1016/j.phytochem.2012.11.001. |  |
| Cannabis flowering | 141 | Happyana, N., and Kayser, O. (2013). Monitoring metabolites production and cannabinoids analysis in medicinal Cannabis trichomes during flowering period by H-1 NMR-based metabolomics. *Planta Med.* 79, 1119. |  |
| Cannabis flowering | 142 | Happyana, N., and Kayser, O. (2016). Monitoring Metabolite Profiles of Cannabis sativa L. Trichomes during Flowering Period Using H-1 NMR-Based Metabolomics and Real-Time PCR. *Planta Med.* 82, 1217–1223. doi:10.1055/s-0042-108058. |  |
| Cannabis flowering | 143 | Happyana, N., and Kayser, O. (2020). Metabolic Changes in the Trichomes of Cannabis sativa var. bedrobinol Analyzed by H-1-NMR-Based Metabolomics. *Indones. J. Chem.* 20, 1246–1254. doi:10.22146/ijc.48765. |  |
| Cannabis flowering | 144 | Hazekamp, A. (2016). Evaluating the Effects of Gamma-Irradiation for Decontamination of Medicinal Cannabis. *Front. Pharmacol.* 7. doi:10.3389/fphar.2016.00108. |  |
| Cannabis flowering | 145 | Hazekamp, A., and Fischedick, J. T. (2012). Cannabis - from cultivar to chemovar. *Drug Test. Anal.* 4, 660–667. doi:10.1002/dta.407. |  |
| Cannabis flowering | 146 | Heidbreder, M., and van Treeck, B. (2019). Cannabis-derivedmedicines for the treatment of chronic pain. Problems resulting frommedical appraisals in the experience of theMedical Advisory Board of the Statutory Health Insurance Funds North. *SCHMERZ* 33, 437–442. doi:10.1007/s00482-019-00397-1. |  |
| Cannabis flowering | 147 | HENNINK, S. (1994). OPTIMIZATION OF BREEDING FOR AGRONOMIC TRAITS IN FIBER HEMP (CANNABIS-SATIVA L) BY STUDY OF PARENT-OFFSPRING RELATIONSHIPS. *EUPHYTICA* 78, 69–76. |  |
| Cannabis flowering | 148 | Herrmann, E. S., Jarvis, B. P., Sparks, A. C., Cohn, A. M., Koszowski, B., Rosenberry, Z. R., et al. (2018). Sweet flowers are slow, and weeds make haste: leveraging methodology from research on tobacco, alcohol, and opioid analgesics to make rapid and policy-relevant advances in cannabis science. *Int. Rev. PSYCHIATRY* 30, 238–250. doi:10.1080/09540261.2018.1465400. |  |
| Cannabis flowering | 149 | HESLOPHARRISON, J. (1960). SUPPRESSIVE EFFECTS OF 2-THIOURACIL ON DIFFERENTIATION AND FLOWERING IN CANNABIS SATIVA. *Science (80-. ).* 132, 1943–1944. doi:10.1126/science.132.3444.1943. |  |
| Cannabis flowering | 150 | Hoppner, F., and Menge-Hartmann, U. (2007). Yield and quality of fibre and oil of fourteen hemp cultivars in Northern Germany at two harvest dates. *Landbauforsch. VOLKENRODE* 57, 219–232. |  |
| Cannabis flowering | 151 | Ingallina, C., Sobolev, A. P., Circi, S., Spano, M., Fraschetti, C., Filippi, A., et al. (2020). Cannabis sativa L. Inflorescences from Monoecious Cultivars Grown in Central Italy: An Untargeted Chemical Characterization from Early Flowering to Ripening. *MOLECULES* 25. doi:10.3390/molecules25081908. |  |
| Cannabis flowering | 152 | Ivanyi, I., and Izsaki, Z. (2008). EFFECT OF SOME AGRONOMIC FACTORS ON THE HEMP (CANNABIS SATIVA L.) YIELD. *Cereal Res. Commun.* 36, 1855–1858. |  |
| Cannabis flowering | 153 | JAISWAL, V. S., and RAM, H. Y. M. (1974). INHIBITION OF GA3 INDUCED EXTENSION GROWTH AND MALE FLOWER FORMATION IN FEMALE PLANTS OF CANNABIS-SATIVA BY CYCLOHEXIMIDE. *Curr. Sci.* 43, 800–801. |  |
| Cannabis flowering | 154 | Jalali, S., Salami, S. A., Sharifi, M., and Sohrabi, S. (2019). Signaling compounds elicit expression of key genes in cannabinoid pathway and related metabolites in cannabis. *Ind. Crops Prod.* 133, 105–110. doi:10.1016/j.indcrop.2019.03.004. |  |
| Cannabis flowering | 155 | Jankauskiene, Z., Gruzdeviene, E., Ivanovs, S., and Maumevicius, E. (2016). INVESTIGATION OF HEMP (CANNABIS SATIVA L.) MORPHOLOGICAL PARAMETERS AS INFLUENCED BY SEED RATE AND GENOTYPE. in *15TH INTERNATIONAL SCIENTIFIC CONFERENCE: ENGINEERING FOR RURAL DEVELOPMENT* Engineering for Rural Development., ed. Malinovska, L and Osadcuks, V, 893–897. |  |
| Cannabis flowering | 156 | Jenkins, B., Buckhalter, S., Perreault, M., and Khokhar, J. (2020). Impact of Vapourized Cannabis Flower on Schizophrenia-Relevant Neural Circuitry and Behaviour in a Neurodevelopmental Rat Model of Schizophrenia. *NEUROPSYCHOPHARMACOLOGY* 45, 345–346. |  |
| Cannabis flowering | 157 | Jenkins, C., and Orsburn, B. (2020). The Cannabis Proteome Draft Map Project. *Int. J. Mol. Sci.* 21. doi:10.3390/ijms21030965. |  |
| Cannabis flowering | 158 | Jikomes, N., and Zoorob, M. (2018). The Cannabinoid Content of Legal Cannabis in Washington State Varies Systematically Across Testing Facilities and Popular Consumer Products. *Sci. Rep.* 8. doi:10.1038/s41598-018-22755-2. |  |
| Cannabis flowering | 159 | Jin, D., Dai, K., Xie, Z., and Chen, J. (2020). Secondary Metabolites Profiled in Cannabis Inflorescences, Leaves, Stem Barks, and Roots for Medicinal Purposes. *Sci. Rep.* 10. doi:10.1038/s41598-020-60172-6. |  |
| Cannabis flowering | 160 | Kaczorova, D., Beres, T., Zeljkovic, S. C., Bjelkova, M., Kuchar, M., and Tarkowski, P. A. (2020). About Cannabis without Prejudice. *Chem. List.* 114, 277–284. |  |
| Cannabis flowering | 161 | Karoly, H. C., Mueller, R. L., Andrade, C. C., and Hutchison, K. E. (2020a). Investigating Relationships Between Alcohol and Cannabis Use in an Online Survey of Cannabis Users: A Focus on Cannabinoid Content and Cannabis for Medical Purposes. *Front. PSYCHIATRY* 11. doi:10.3389/fpsyt.2020.613243. |  |
| Cannabis flowering | 162 | Karoly, H., Torres, M., Gust, C., Jones, M., Bryan, A., Hutchison, K., et al. (2020b). Acute Objective and Subjective Effects of Cannabis Among Flower Versus Edible Users. *NEUROPSYCHOPHARMACOLOGY* 45, 155. |  |
| Cannabis flowering | 163 | Keller, A., Leupin, M., Mediavilla, V., and Wintermantel, E. (2001). Influence of the growth stage of industrial hemp on chemical and physical properties of the fibres. *Ind. Crops Prod.* 13, 35–48. doi:10.1016/S0926-6690(00)00051-0. |  |
| Cannabis flowering | 164 | Keshet, U., Alon, T., Fialkov, A. B., and Amirav, A. (2017). Open Probe fast GC-MS - combining ambient sampling ultra-fast separation and in-vacuum ionization for real-time analysis. *J. MASS Spectrom.* 52, 417–426. doi:10.1002/jms.3941. |  |
| Cannabis flowering | 165 | Khan, A. M., Qureshi, R., Saqib, Z., Habib, T., Ilyas, M., Maqsood, M., et al. (2018). A NOVEL STUDY OF THE INTERRELATIONSHIP OF SEASONALITY, SATELLITE DATA AND WEED COMPOSITIONAL CHANGES OF THE AGRO-ECOLOGICAL SYSTEM OF GUJRAT, PAKISTAN. *Appl. Ecol. Environ. Res.* 16, 2995–3018. doi:10.15666/aeer/1603_29953018. |  |
| Cannabis flowering | 166 | Khan, H., Marwat, K. B., Hassan, G., Khan, M. A., and Hashim, S. (2014). DISTRIBUTION OF PARTHENIUM WEED IN PESHAWAR VALLEY, KHYBER PAKHTUNKHWA- PAKISTAN. *PAKISTAN J. Bot.* 46, 81–90. |  |
| Cannabis flowering | 167 | Kiefer, A., Eckert-Lill, C., Bussick, D., Hoernig, M., and Reimann, H. (2019). Cannabis for medical purposes and its prescription. *BUNDESGESUNDHEITSBLATT-GESUNDHEITSFORSCHUNG-GESUNDHEITSSCHUTZ* 62, 811–817. doi:10.1007/s00103-019-02970-6. |  |
| Cannabis flowering | 168 | Kleinhenz, M. D., Magnin, G., Ensley, S. M., Griffin, J. J., Goeser, J., Lynch, E., et al. (2020). Nutrient concentrations, digestibility, and cannabinoid concentrations of industrial hemp plant components. *Appl. Anim. Sci.* 36, 489–494. doi:10.15232/aas.2020-02018. |  |
| Cannabis flowering | 169 | Knight, G., Hansen, S., Connor, M., Poulsen, H., McGovern, C., and Stacey, J. (2010). The results of an experimental indoor hydroponic Cannabis growing study, using the ‘Screen of Green’(ScrOG) method—Yield, tetrahydrocannabinol (THC) and DNA analysis. *Forensic Sci. Int.* 202, 36–44. |  |
| Cannabis flowering | 170 | Knoess, W., van de Velde, M., Sandvos, C., and Cremer-Schaeffer, P. (2019). Key elements of legal environments for medical use of cannabis in different countries. *BUNDESGESUNDHEITSBLATT-GESUNDHEITSFORSCHUNG-GESUNDHEITSSCHUTZ* 62, 855–860. doi:10.1007/s00103-019-02969-z. |  |
| Cannabis flowering | 171 | Kovalchuk, O., Li, D., Rodriguez-Juarez, R., Golubov, A., Hudson, D., and Kovalchuk, I. (2020). The effect of cannabis dry flower irradiation on the level of cannabinoids, terpenes and anti-cancer properties of the extracts. *Biocatal. Agric. Biotechnol.* 29. doi:10.1016/j.bcab.2020.101736. |  |
| Cannabis flowering | 172 | Kowalski, J., Laine, D., Minier, S., and Johnson, C. (2018). Cleaning up cannabinoids and chlorophyll: Optimization of sample preparation for pesticide analysis in cannabis flower. *Abstr. Pap. Am. Chem. Soc.* 255. |  |
| Cannabis flowering | 173 | Kwasnica, A., Pachura, N., Masztalerz, K., Figiel, A., Zimmer, A., Kupczynski, R., et al. (2020). Volatile Composition and Sensory Properties as Quality Attributes of Fresh and Dried Hemp Flowers (Cannabis sativa L.). *FOODS* 9. doi:10.3390/foods9081118. |  |
| Cannabis flowering | 174 | Lader, M. (2009). Addiction and the pharmacology of cannabis: implications for medicine and the law. *Med. Sci. LAW* 49, 1–17. |  |
| Cannabis flowering | 175 | Lamy, F. R., Daniulaityte, R., Zatreh, M., Nahhas, R. W., Sheth, A., Martins, S. S., et al. (2018). ``You got to love rosin: Solventless dabs, pure, clean, natural medicine.{’’} Exploring Twitter data on emerging trends in Rosin Tech marijuana concentrates. *Drug Alcohol Depend.* 183, 248–252. doi:10.1016/j.drugalcdep.2017.10.039. |  |
| Cannabis flowering | 176 | Landi, S., Berni, R., Capasso, G., Hausman, J.-F., Guerriero, G., and Esposito, S. (2019). Impact of Nitrogen Nutrition on Cannabis sativa: An Update on the Current Knowledge and Future Prospects. *Int. J. Mol. Sci.* 20. doi:10.3390/ijms20225803. |  |
| Cannabis flowering | 177 | Laza, A., Orozco, E., Baldo, M. F., Raba, J., and Aranda, P. R. (2020). Determination of arsenic (V) in cannabis oil by adsorption on multiwall carbon nanotubes thin film using XRF technique. *Microchem. J.* 158. doi:10.1016/j.microc.2020.105265. |  |
| Cannabis flowering | 178 | LEMBERKOVICS, E., VESZKI, P., VERZARPETRI, G., and TRKA, A. (1979). CONTRIBUTIONS TO THE ESSENTIAL OIL COMPOSITION OF THE FLOWERS AND LEAVES OF CANNABIS-SATIVA L. *Planta Med.* 36, 271–272. |  |
| Cannabis flowering | 179 | Leme, F. M., Borella, P. H., Marinho, C. R., and Teixeira, S. P. (2020a). Expanding the laticifer knowledge in Cannabaceae: distribution, morphology, origin, and latex composition. *Protoplasma* 257, 1183–1199. doi:10.1007/s00709-020-01500-5. |  |
| Cannabis flowering | 180 | Leme, F. M., Schonenberger, J., Staedler, Y. M., and Teixeira, S. P. (2020b). Comparative floral development reveals novel aspects of structure and diversity of flowers in Cannabaceae. *Bot. J. Linn. Soc.* 193, 64–83. doi:10.1093/botlinnean/boaa004. |  |
| Cannabis flowering | 181 | Li, K., Zhang, W.-B., Zhao, Z.-Y., Zhao, Y., Chen, X.-W., and Kong, L.-B. (2018). A porous carbon material from pyrolysis of fructus cannabis’s shells for supercapacitor electrode application. *Mater. Res. EXPRESS* 5. doi:10.1088/2053-1591/aaad70. |  |
| Cannabis flowering | 182 | Li, X., Diviant, J. P., Stith, S. S., Brockelman, F., Keeling, K., Hall, B., et al. (2020). The Effectiveness of Cannabis Flower for Immediate Relief from Symptoms of Depression. *YALE J. Biol. Med.* 93, 251–264. |  |
| Cannabis flowering | 183 | Li, X., Vigil, J. M., Stith, S. S., Brockelman, F., Keeling, K., and Hall, B. (2019). The effectiveness of self-directed medical cannabis treatment for pain. *Complement. Ther. Med.* 46, 123–130. doi:10.1016/j.ctim.2019.07.022. |  |
| Cannabis flowering | 184 | Lisson, S. N., and Mendham, N. J. (2000). Cultivar, sowing date and plant density studies of fibre hemp (Cannabis sativa L.) in Tasmania. *Aust. J. Exp. Agric.* 40, 975–986. doi:10.1071/EA99130. |  |
| Cannabis flowering | 185 | Lisson, S. N., Mendham, N. J., and Carberry, P. S. (2000). Development of a hemp (Cannabis sativa L.) simulation model 2. The flowering response of two hemp cultivars to photoperiod. *Aust. J. Exp. Agric.* 40, 413–417. doi:10.1071/EA99059. |  |
| Cannabis flowering | 186 | Liu, M., Fernando, D., Daniel, G., Madsen, B., Meyer, A. S., Ale, M. T., et al. (2015). Effect of harvest time and field retting duration on the chemical composition, morphology and mechanical properties of hemp fibers. *Ind. Crops Prod.* 69, 29–39. doi:10.1016/j.indcrop.2015.02.010. |  |
| Cannabis flowering | 187 | Liu, Y., and Yang, G. (2014). Tc1-like transposable elements in plant genomes. *Mob. DNA* 5. doi:10.1186/1759-8753-5-17. |  |
| Cannabis flowering | 188 | Livingston, S. J., Quilichini, T. D., Booth, J. K., Wong, D. C. J., Rensing, K. H., Laflamme-Yonkman, J., et al. (2020). Cannabis glandular trichomes alter morphology and metabolite content during flower maturation. *PLANT J.* 101, 37–56. doi:10.1111/tpj.14516. |  |
| Cannabis flowering | 189 | Loflin, M., and Earleywine, M. (2014). A new method of cannabis ingestion: The dangers of dabs? *Addict. Behav.* 39, 1430–1433. doi:10.1016/j.addbeh.2014.05.013. |  |
| Cannabis flowering | 190 | Lubell, J. D., and Brand, M. H. (2018). Foliar Sprays of Silver Thiosulfate Produce Male Flowers on Female Hemp Plants. *Horttechnology* 28, 743–747. doi:10.21273/HORTTECH04188-18. |  |
| Cannabis flowering | 191 | Luc, M. H., Tsang, S. W., Thrul, J., Kennedy, R. D., and Moran, M. B. (2020). Content analysis of online product descriptions from cannabis retailers in six US states. *Int. J. DRUG POLICY* 75. doi:10.1016/j.drugpo.2019.10.017. |  |
| Cannabis flowering | 192 | Macherone, A., Jordan, R., Miller, D., Asanuma, L., Roy, J.-F., and Stone, P. J. (2018). A Multiplatform Approach to Residual Pesticide Quantitation in Cannabis Flower for the California and Canadian Target Lists. *Am. Lab.* 50, 14–16. |  |
| Cannabis flowering | 193 | Maguire, W. J., Call, C. W., Cerbu, C., Jambor, K. L., and Benavides-Montes, V. E. (2019). Comprehensive Determination of Unregulated Pesticide Residues in Oregon Cannabis Flower by Liquid Chromatography Paired with Triple Quadrupole Mass Spectrometry and Gas Chromatography Paired with Triple Quadrupole Mass Spectrometry. *J. Agric. Food Chem.* 67, 12670–12674. doi:10.1021/acs.jafc.9b01559. |  |
| Cannabis flowering | 194 | Mankowska, G., and Silska, G. (2015). Genetic Resources of Cannabis sativa L. in the Collection of the Gene Bank at INF&MP in Poznan. *J. Nat. FIBERS* 12, 332–340. doi:10.1080/15440478.2014.928246. |  |
| Cannabis flowering | 195 | Manns, D., Norwig, J., and Reh, K. (2019). Cannabis for medicinal use: development of pharmacopoeia monographs as a quality standard. *BUNDESGESUNDHEITSBLATT-GESUNDHEITSFORSCHUNG-GESUNDHEITSSCHUTZ* 62, 806–810. doi:10.1007/s00103-019-02963-5. |  |
| Cannabis flowering | 196 | Mansouri, H., Asrar, Z., and Mehrabani, M. (2009). Effects of Gibberellic Acid on Primary Terpenoids and Delta(9)-Tetrahydrocannabinol in Cannabis sativa at Flowering Stage. *J. Integr. Plant Biol.* 51, 553–561. doi:10.1111/j.1744-7909.2009.00833.x. |  |
| Cannabis flowering | 197 | Mansouri, H., Salari, F., and Asrar, Z. (2013). Ethephon application stimulats cannabinoids and plastidic terpenoids production in Cannabis sativa at flowering stage. *Ind. Crops Prod.* 46, 269–273. doi:10.1016/j.indcrop.2013.01.025. |  |
| Cannabis flowering | 198 | Manthey, J., Carr, S., and Rehm, J. (2020). Definition of a `standard joint equivalent’: Comment on ``Who consumes most of the cannabis in Canada? Profiles of cannabis consumption by quantity{’’}. *Drug Alcohol Depend.* 206. doi:10.1016/j.drugalcdep.2019.107731. |  |
| Cannabis flowering | 199 | Martinez, V., Iriondo De-Hond, A., Borrelli, F., Capasso, R., del Castillo, M., and Abalo, R. (2020). Cannabidiol and Other Non-Psychoactive Cannabinoids for Prevention and Treatment of Gastrointestinal Disorders: Useful Nutraceuticals? *Int. J. Mol. Sci.* 21. doi:10.3390/ijms21093067. |  |
| Cannabis flowering | 200 | Mazian, B., Bergeret, A., Benezet, J.-C., and Malhautier, L. (2018). Influence of field retting duration on the biochemical, microstructural, thermal and mechanical properties of hemp fibres harvested at the beginning of flowering. *Ind. Crops Prod.* 116, 170–181. doi:10.1016/j.indcrop.2018.02.062. |  |
| Cannabis flowering | 201 | McArdle, P. A. (2006). Cannabis use by children and young people. *Arch. Dis. Child.* 91, 692–695. doi:10.1136/adc.2005.071860. |  |
| Cannabis flowering | 202 | McGarvey, P., Huang, J., McCoy, M., Orvis, J., Katsir, Y., Lotringer, N., et al. (2020). De novo assembly and annotation of transcriptomes from two cultivars of Cannabis sativa with diff erent cannabinoid profiles. *Gene* 762. doi:10.1016/j.gene.2020.145026. |  |
| Cannabis flowering | 203 | McGehee, C. S., Apicella, P., Raudales, R., Berkowitz, G., Ma, Y., Durocher, S., et al. (2019). First Report of Root Rot and Wilt Caused by Pythium myriotylum on Hemp (Cannabis sativa) in the United States. *PLANT Dis.* 103, 3288. doi:10.1094/PDIS-11-18-2028-PDN. |  |
| Cannabis flowering | 204 | Mediavilla, V., Leupin, M., and Keller, A. (2001). Influence of the growth stage of industrial hemp on the yield formation in relation to certain fibre quality traits. *Ind. Crops Prod.* 13, 49–56. doi:10.1016/S0926-6690(00)00052-2. |  |
| Cannabis flowering | 205 | Meija, J., McRae, G., Miles, C. O., and Melanson, J. E. Thermal stability of cannabinoids in dried cannabis: a kinetic study. *Anal. Bioanal. Chem.* doi:10.1007/s00216-020-03098-2. |  |
| Cannabis flowering | 206 | Merlin, M. D. (2003). Archaeological evidence for the tradition of psychoactive plant use in the old world. *Econ. Bot.* 57, 295–323. doi:10.1663/0013-0001(2003)057{[}0295:AEFTTO]2.0.CO;2. |  |
| Cannabis flowering | 207 | Miller, B. L., Stogner, J. M., and Miller, J. M. (2016). Exploring Butane Hash Oil Use: A Research Note. *J. Psychoactive Drugs* 48, 44–49. doi:10.1080/02791072.2015.1118173. |  |
| Cannabis flowering | 208 | Mizia, P., Cygan, M., Denysenko, M., Kwolek, D., Chramiec-Glabik, A., Grabowska-Joachimiak, A., et al. (2016). DEVELOPMENT OF A RAPD-BASED MALE-SPECIFIC MOLECULAR MARKER IN JAPANESE HOP (HUMULUS JAPONICUS SIEBOLD & ZUCC.). *ACTA Biol. CRACOVIENSIA Ser. Bot.* 58, 21–27. doi:10.1515/abcsb-2016-0017. |  |
| Cannabis flowering | 209 | MOHANRAM, H. Y., and JAISWAL, V. S. (1971). FEMINIZATION OF MALE FLOWERS OF CANNABIS-SATIVA L BY A MORPHACTIN. *NATURWISSENSCHAFTEN* 58, 149+. doi:10.1007/BF00593110. |  |
| Cannabis flowering | 210 | MOHANRAM, H. Y., and JAISWAL, V. S. (1972). INDUCTION OF MALE FLOWERS ON FEMALE PLANTS OF CANNABIS-SATIVA BY GIBBERELLINS AND ITS INHIBITION BY ABSCISIC ACID. *Planta* 105, 263-. doi:10.1007/BF00385397. |  |
| Cannabis flowering | 211 | Moher, M., Jones, M., and Zheng, Y. (2021). Photoperiodic Response of In Vitro Cannabis sativa Plants. *HORTSCIENCE* 56, 108–113. doi:10.21273/HORTSCI15452-20. |  |
| Cannabis flowering | 212 | Moliterni, V. M. C., Cattivelli, L., Ranalli, P., and Mandolino, G. (2004). The sexual differentiation of Cannabis sativa L.: A morphological and molecular study. *EUPHYTICA* 140, 95–106. doi:10.1007/s10681-004-4758-7. |  |
| Cannabis flowering | 213 | Mondino, A., Fernandez, S., Garcia-Carnelli, C., Castro, M. J., Umpierrez, E., Torterolo, P., et al. (2019). Vaporized Cannabis differentially modulates sexual behavior of female rats according to the dose. *Pharmacol. Biochem. Behav.* 187. doi:10.1016/j.pbb.2019.172814. |  |
| Cannabis flowering | 214 | Monthony, A. S., and Jones, A. M. P. (2020). Flower Power: A Rapid In Vitro Regeneration Protocol from In Vitro Cannabis sativa Inflorescences. *Vitr. Cell. Dev. Biol.* 56, S25. |  |
| Cannabis flowering | 215 | Moreno-Sanz, G. (2016). Can You Pass the Acid Test? Critical Review and Novel Therapeutic Perspectives of Delta(9)-Tetrahydrocannabinolic Acid A. *CANNABIS CANNABINOID Res.* 1, 124–130. doi:10.1089/can.2016.0008. |  |
| Cannabis flowering | 216 | Moreno, T., Montanes, F., Tallon, S. J., Fenton, T., and King, J. W. (2020). Extraction of cannabinoids from hemp (Cannabis sativa L.) using high pressure solvents: An overview of different processing options. *J. Supercrit. Fluids* 161. doi:10.1016/j.supflu.2020.104850. |  |
| Cannabis flowering | 217 | Moulins, J. R., Blais, M., Montsion, K., Tully, J., Mohan, W., Gagnon, M., et al. (2018). Multiresidue Method of Analysis of Pesticides in Medical Cannabis. *J. AOAC Int.* 101, 1948–1960. doi:10.5740/jaoacint.17-0495. |  |
| Cannabis flowering | 218 | Mudge, E. M., and Brown, P. N. (2020). Determination of Cannabinoids in Cannabis sativa Dried Flowers and Oils by LC-UV: Single-Laboratory Validation, First Action 2018.10. *J. AOAC Int.* 103, 489–493. doi:10.5740/jaoacint.19-0197. |  |
| Cannabis flowering | 219 | Mudge, E. M., Murch, S. J., and Brown, P. N. (2017). Leaner and greener analysis of cannabinoids. *Anal. Bioanal. Chem.* 409, 3153–3163. doi:10.1007/s00216-017-0256-3. |  |
| Cannabis flowering | 220 | Muessig, J., Haag, K., Musio, S., Bjelkova, M., Albrecht, K., Uhrlaub, B., et al. (2020). Biobased `Mid-performance’ composites using losses from the hackling process of long hemp - A feasibility study as part of the development of a biorefinery concept-. *Ind. Crops Prod.* 145. doi:10.1016/j.indcrop.2019.111938. |  |
| Cannabis flowering | 221 | Muscara, C., Smeriglio, A., Trombetta, D., Mandalari, G., La Camera, E., Occhiuto, C., et al. Antioxidant and antimicrobial activity of two standardized extracts from a new Chinese accession of non-psychotropicCannabis sativaL. *Phyther. Res.* doi:10.1002/ptr.6891. |  |
| Cannabis flowering | 222 | Musio, S., Muessig, J., and Amaducci, S. (2018). Optimizing Hemp Fiber Production for High Performance Composite Applications. *Front. Plant Sci.* 9. doi:10.3389/fpls.2018.01702. |  |
| Cannabis flowering | 223 | Naftali, T., Schleider, L. B.-L., Dotan, I., Lansky, E. P., Benjaminov, F. S., and Konikoff, F. M. (2013). Cannabis Induces a Clinical Response in Patients With Crohn’s Disease: A Prospective Placebo-Controlled Study. *Clin. Gastroenterol. Hepatol.* 11, 1276+. doi:10.1016/j.cgh.2013.04.034. |  |
| Cannabis flowering | 224 | Nagy, D. U., Cianfaglione, K., Maggi, F., Sut, S., and Dall’Acqua, S. (2019). Chemical Characterization of Leaves, Male and Female Flowers from Spontaneous Cannabis (Cannabis sativa L.) Growing in Hungary. *Chem. Biodivers.* 16. doi:10.1002/cbdv.201800562. |  |
| Cannabis flowering | 225 | Nallathambi, R., Mazuz, M., Ion, A., Selvaraj, G., Weininger, S., Fridlender, M., et al. (2017). Anti-Inflammatory Activity in Colon Models Is Derived from Delta 9-Tetrahydrocannabinolic Acid That Interacts with Additional Compounds in Cannabis Extracts. *CANNABIS CANNABINOID Res.* 2, 167–182. doi:10.1089/can.2017.0027. |  |
| Cannabis flowering | 226 | Namdar, D., Charuvi, D., Ajjampura, V., Mazuz, M., Ion, A., Kamara, I., et al. (2019). LED lighting affects the composition and biological activity of Cannabis sativa secondary metabolites. *Ind. Crops Prod.* 132, 177–185. doi:10.1016/j.indcrop.2019.02.016. |  |
| Cannabis flowering | 227 | Namdar, D., Mazuz, M., Ion, A., and Koltai, H. (2018). Variation in the compositions of cannabinoid and terpenoids in Cannabis sativa derived from inflorescence position along the stem and extraction methods. *Ind. Crops Prod.* 113, 376–382. doi:10.1016/j.indcrop.2018.01.060. |  |
| Cannabis flowering | 228 | NIGAM, R. K. (1983). SOME NUCLEIC-ACID BASES AND THEIR EFFECT ON THE RATE OF GROWTH AND FLOWERING IN CANNABIS-SATIVA L .3. EFFECT OF URACIL. *Comp. Physiol. Ecol.* 8, 362–364. |  |
| Cannabis flowering | 229 | NIGAM, R. K., VARKEY, M., and REUBEN, D. E. (1981). IRRADIATION INDUCED CHANGES IN FLOWER FORMATION IN CANNABIS-SATIVA L. *Biol. Plant.* 23, 389–391. doi:10.1007/BF02877422. |  |
| Cannabis flowering | 230 | Noestheden, M., Friedlander, G., Anspach, J., Krepich, S., Hyland, K. C., and Zandberg, W. F. (2018). Chromatographic characterisation of 11 phytocannabinoids: Quantitative and fit-to-purpose performance as a function of extra-column variance. *Phytochem. Anal.* 29, 507–515. doi:10.1002/pca.2761. |  |
| Cannabis flowering | 231 | Nsuala, B. N., Enslin, G., and Viljoen, A. (2015). ``Wild cannabis{’’}: A review of the traditional use and phytochemistry of Leonotis leonurus. *J. Ethnopharmacol.* 174, 520–539. doi:10.1016/j.jep.2015.08.013. |  |
| Cannabis flowering | 232 | O’Brien, C., and Arathi, H. S. (2019). Bee diversity and abundance on flowers of industrial hemp (Cannabis sativa L.). *Biomass Bioenergy* 122, 331–335. doi:10.1016/j.biombioe.2019.01.015. |  |
| Cannabis flowering | 233 | Okey, S. A., and Meier, M. H. (2020). A within-person comparison of the subjective effects of higher vs. lower-potency cannabis. *Drug Alcohol Depend.* 216. doi:10.1016/j.drugalcdep.2020.108225. |  |
| Cannabis flowering | 234 | Orenstein, D. G., and Glantz, S. A. (2018). Regulating Cannabis Manufacturing: Applying Public Health Best Practices from Tobacco Control. *J. Psychoactive Drugs* 50, 19–32. doi:10.1080/02791072.2017.1422816. |  |
| Cannabis flowering | 235 | Pacifici, R., Marchei, E., Salvatore, F., Guandalini, L., Busardo, F. P., and Pichini, S. (2017). Evaluation of cannabinoids concentration and stability in standardized preparations of cannabis tea and cannabis oil by ultra-high performance liquid chromatography tandem mass spectrometry. *Clin. Chem. Lab. Med.* 55, 1555–1563. doi:10.1515/cclm-2016-1060. |  |
| Cannabis flowering | 236 | Pacifici, R., Marchei, E., Salvatore, F., Guandalini, L., Busardo, F. P., and Pichini, S. (2018). Evaluation of long-term stability of cannabinoids in standardized preparations of cannabis flowering tops and cannabis oil by ultra-high-performance liquid chromatography tandem mass spectrometry. *Clin. Chem. Lab. Med.* 56, E94–E96. doi:10.1515/cclm-2017-0758. |  |
| Cannabis flowering | 237 | Pacifici, R., Marchei, E., Salvatore, F., Guandalini, L., Busardo, F. P., and Pichini, S. (2019a). Stability of cannabinoids in cannabis FM1 flowering tops and oil preparation evaluated by ultra-high performance liquid chromatography tandem mass spectrometry. *Clin. Chem. Lab. Med.* 57, E165–E168. doi:10.1515/cclm-2018-1145. |  |
| Cannabis flowering | 238 | Pacifici, R., Pichini, S., Pellegrini, M., Tittarelli, R., Pantano, F., Mannocchi, G., et al. (2019b). Determination of cannabinoids in oral fluid and urine of ``light cannabis{’’} consumers: a pilot study. *Clin. Chem. Lab. Med.* 57, 238–243. doi:10.1515/cclm-2018-0566. |  |
| Cannabis flowering | 239 | Pagnania, G., Pellegrini, M., Galieni, A., D’Egidio, S., Matteucci, F., Ricci, A., et al. (2018). Plant growth-promoting rhizobacteria (PGPR) in Cannabis sativa `Finola’ cultivation: An alternative fertilization strategy to improve plant growth and quality characteristics. *Ind. Crops Prod.* 123, 75–83. doi:10.1016/j.indcrop.2018.06.033. |  |
| Cannabis flowering | 240 | Peil, A., Flachowsky, H., Schumann, E., and Weber, W. E. (2003). Sex-linked AFLP markers indicate a pseudoautosomal region in hemp (Cannabis sativa L.). *Theor. Appl. Genet.* 107, 102–109. doi:10.1007/s00122-003-1212-5. |  |
| Cannabis flowering | 241 | Pepper, J. K., Lee, Y. O., Eggers, M. E., Allen, J. A., Thompson, J., and Nonnemaker, J. M. (2020). Perceptions of US and Canadian Cannabis Package Warnings Among US Adults. *Drug Alcohol Depend.* 217. doi:10.1016/j.drugalcdep.2020.108275. |  |
| Cannabis flowering | 242 | Perez-Acevedo, A. P., Pacifici, R., Mannocchi, G., Gottardi, M., Poyatos, L., Papaseit, E., et al. Disposition of cannabinoids and their metabolites in serum, oral fluid, sweat patch and urine from healthy individuals treated with pharmaceutical preparations of medical cannabis. *Phyther. Res.* doi:10.1002/ptr.6931. |  |
| Cannabis flowering | 243 | Peschel, W. (2016). Quality Control of Traditional Cannabis Tinctures: Pattern, Markers, and Stability. *Sci. Pharm.* 84, 567–584. doi:10.3390/scipharm84030567. |  |
| Cannabis flowering | 244 | Petit, J., Salentijn, E. M. J., Paulo, M.-J., Denneboom, C., and Trindade, L. M. (2020a). Genetic Architecture of Flowering Time and Sex Determination in Hemp (Cannabis sativa L.): A Genome-Wide Association Study. *Front. Plant Sci.* 11. doi:10.3389/fpls.2020.569958. |  |
| Cannabis flowering | 245 | Petit, J., Salentijn, E. M. J., Paulo, M.-J., Thouminot, C., van Dinter, B. J., Magagnini, G., et al. (2020b). Genetic Variability of Morphological, Flowering, and Biomass Quality Traits in Hemp (Cannabis sativa L.). *Front. Plant Sci.* 11. doi:10.3389/fpls.2020.00102. |  |
| Cannabis flowering | 246 | Petzke, F., Karst, M., Gastmeier, K., Radbruch, L., Steffen, E., and Haeuser, W. (2019). Position paper onmedical cannabis and cannabis-basedmedicines in pain medicine. *SCHMERZ* 33, 449–465. doi:10.1007/s00482-019-00407-2. |  |
| Cannabis flowering | 247 | Pilar Perez-Acevedo, A., Paolo Busardo, F., Pacifici, R., Mannocchi, G., Gottardi, M., Poyatos, L., et al. (2020). Disposition of Cannabidiol Metabolites in Serum and Urine from Healthy Individuals Treated with Pharmaceutical Preparations of Medical Cannabis. *PHARMACEUTICALS* 13. doi:10.3390/ph13120459. |  |
| Cannabis flowering | 248 | Piunno, K. F., Golenia, G., Boudko, E. A., Downey, C., and Jones, A. M. P. (2019). Regeneration of shoots from immature and mature inflorescences of Cannabis sativa. *Can. J. PLANT Sci.* 99, 556–559. doi:10.1139/cjps-2018-0308. |  |
| Cannabis flowering | 249 | Popova, L., McDonald, E. A., Sidhu, S., Barry, R., Maruyama, T. A. R., Sheon, N. M., et al. (2017). Perceived harms and benefits of tobacco, marijuana, and electronic vaporizers among young adults in Colorado: implications for health education and research. *ADDICTION* 112, 1821–1829. doi:10.1111/add.13854. |  |
| Cannabis flowering | 250 | Potter, D. J., and Duncombe, P. (2012). The Effect of Electrical Lighting Power and Irradiance on Indoor-Grown Cannabis Potency and Yield. *J. Forensic Sci.* 57, 618–622. doi:10.1111/j.1556-4029.2011.02024.x. |  |
| Cannabis flowering | 251 | Poulsen, H. A., and Sutherland, G. J. (2000). The potency of cannabis in New Zealand from 1976 to 1996. *Sci. JUSTICE* 40, 171–176. doi:10.1016/S1355-0306(00)71972-1. |  |
| Cannabis flowering | 252 | Presto, M. H., Lyberg, K., and Lindberg, J. E. (2011). Digestibility of amino acids in organically cultivated white-flowering faba bean and cake from cold-pressed rapeseed, linseed and hemp seed in growing pigs. *Arch. Anim. Nutr.* 65, 21–33. doi:10.1080/1745039X.2010.534897. |  |
| Cannabis flowering | 253 | Prince, M. A., and Conner, B. T. (2019). Examining links between cannabis potency and mental and physical health outcomes. *Behav. Res. Ther.* 115, 111–120. doi:10.1016/j.brat.2018.11.008. |  |
| Cannabis flowering | 254 | Prince, M. A., Conner, B. T., and Pearson, M. R. (2018). Quantifying Cannabis: A Field Study of Marijuana Quantity Estimation. *Psychol. Addict. Behav.* 32, 426–433. doi:10.1037/adb0000370. |  |
| Cannabis flowering | 255 | Pritchard, F. J. (1916). Change of sex in hemp - Mutilation makes female plants of cannabis sativa produce male flowers - Change in nutrition probably responsible for the result. *J. Hered.* 7, 325–329. doi:10.1093/oxfordjournals.jhered.a110739. |  |
| Cannabis flowering | 256 | Punja, Z. K. Epidemiology ofFusarium oxysporumcausing root and crown rot of cannabis (Cannabis sativaL., marijuana) plants in commercial greenhouse production. *Can. J. PLANT Pathol.* doi:10.1080/07060661.2020.1788165. |  |
| Cannabis flowering | 257 | Punja, Z. K. First report ofFusarium proliferatumcausing crown and stem rot, and pith necrosis, in cannabis (Cannabis sativaL., marijuana) plants. *Can. J. PLANT Pathol.* doi:10.1080/07060661.2020.1793222. |  |
| Cannabis flowering | 258 | Punja, Z. K. (2018). Flower and foliage-infecting pathogens of marijuana (Cannabis sativa L.) plants. *Can. J. PLANT Pathol.* 40, 514–527. doi:10.1080/07060661.2018.1535467. |  |
| Cannabis flowering | 259 | Punja, Z. K., and Holmes, J. E. (2020). Hermaphroditism in Marijuana (Cannabis sativaL.) Inflorescences - Impact on Floral Morphology, Seed Formation, Progeny Sex Ratios, and Genetic Variation. *Front. Plant Sci.* 11. doi:10.3389/fpls.2020.00718. |  |
| Cannabis flowering | 260 | Radoiu, M., Kaur, H., Bakowska-Barczak, A., and Splinter, S. (2020). Microwave-Assisted Industrial Scale Cannabis Extraction. *TECHNOLOGIES* 8. doi:10.3390/technologies8030045. |  |
| Cannabis flowering | 261 | Raharjo, T. J., Chang, W. T., Choi, Y. H., Peltenburg-Looman, A. M. G., and Verpoorte, R. (2004). Olivetol as product of a polyketide synthase in Cannabis saliva L. *PLANT Sci.* 166, 381–385. doi:10.1016/j.plantsci.2003.09.027. |  |
| Cannabis flowering | 262 | RAM, H. Y. M., and JAISWAL, V. S. (1970). INDUCTION OF FEMALE FLOWERS ON MALE PLANTS OF CANNABIS-SATIVA L BY 2-CHLOROETHANEPHOS-PHONIC ACID. *Experientia* 26, 214-. doi:10.1007/BF01895593. |  |
| Cannabis flowering | 263 | RAM, H. Y. M., and SETT, R. (1982). INDUCTION OF FERTILE MALE FLOWERS IN GENETICALLY FEMALE CANNABIS-SATIVA PLANTS BY SILVER-NITRATE AND SILVER THIOSULFATE ANIONIC COMPLEX. *Theor. Appl. Genet.* 62, 369–375. |  |
| Cannabis flowering | 264 | Rana, T. S., and Datt, B. (1997). Ethnobotanical observation among Jaunsaris of Jaunsar-Bawar, Dehra Dun (UP), India. *Int. J. Pharmacogn.* 35, 371–374. doi:10.1080/09251619708951285. |  |
| Cannabis flowering | 265 | Rao, G. G. P. (2018). Molecular characterization of phytoplasma associated with four important ornamental plant species in India and identification of natural potential spread sources. *3 BIOTECH* 8. doi:10.1007/s13205-018-1126-1. |  |
| Cannabis flowering | 266 | Richins, R. D., Rodriguez-Uribe, L., Lowe, K., Ferral, R., and O’Connell, M. A. (2018). Accumulation of bioactive metabolites in cultivated medical Cannabis. *PLoS One* 13, e0201119. doi:10.1371/journal.pone.0201119. |  |
| Cannabis flowering | 267 | Riggle, J., Nilsson, Z., and Spikerman, D. (2016). Extraction and quantitation of cannabinoids in locally grown medicinal cannabis flowers and other extraction products. *Abstr. Pap. Am. Chem. Soc.* 251. |  |
| Cannabis flowering | 268 | Rodziewicz, P., Loroch, S., Marczak, L., Sickmann, A., and Kayser, O. (2019). Cannabinoid synthases and osmoprotective metabolites accumulate in the exudates of Cannabis sativa L. glandular trichomes. *PLANT Sci.* 284, 108–116. doi:10.1016/j.plantsci.2019.04.008. |  |
| Cannabis flowering | 269 | Roggen, M. (2019). Healthy and Concentrated Cannabis Plants: How to Use Acronyms to Optimize Production. *J. AOAC Int.* 102, 421–426. doi:10.5740/jaoacint.18-0205. |  |
| Cannabis flowering | 270 | Rolli, E., Marieschi, M., Maietti, S., Sacchetti, G., and Bruni, R. (2014). Comparative phytotoxicity of 25 essential oils on pre- and post-emergence development of Solanum lycopersicum L.: A multivariate approach. *Ind. Crops Prod.* 60, 280–290. doi:10.1016/j.indcrop.2014.06.021. |  |
| Cannabis flowering | 271 | Romero, P., Peris, A., Vergara, K., and Matus, J. T. (2020). Comprehending and improving cannabis specialized metabolism in the systems biology era. *PLANT Sci.* 298. doi:10.1016/j.plantsci.2020.110571. |  |
| Cannabis flowering | 272 | Rotermann, M. (2019). Analysis of trends in the prevalence of cannabis use and related metrics in Canada. *Heal. REPORTS* 30, 3–13. doi:10.25318/82-003-x201900600001-eng. |  |
| Cannabis flowering | 273 | Rothschild, M., Bergstrom, G., and Wangberg, S. A. (2005). Cannabis sativa: volatile compounds from pollen and entire male and female plants of two variants, Northern Lights and Hawaian Indica. *Bot. J. Linn. Soc.* 147, 387–397. doi:10.1111/j.1095-8339.2005.00417.x. |  |
| Cannabis flowering | 274 | Ruchlemer, R., Amit-Kohn, M., Raveh, D., and Hanus, L. (2015). Inhaled medicinal cannabis and the immunocompromised patient. *Support. CARE CANCER* 23, 819–822. doi:10.1007/s00520-014-2429-3. |  |
| Cannabis flowering | 275 | Ruiz Contreras, A. E., Mendez Diaz, M., Prieto Gomez, B., Romano, A., Caynas, S., and Prospero Garcia, O. (2010). Brain, drugs and genes. *SALUD Ment.* 33, 535–542. |  |
| Cannabis flowering | 276 | Sagar, K. A., Lambros, A. M., Dahlgren, M. K., Smith, R. T., and Gruber, S. A. (2018). Made from concentrate? A national web survey assessing dab use in the United States. *Drug Alcohol Depend.* 190, 133–142. doi:10.1016/j.drugalcdep.2018.05.022. |  |
| Cannabis flowering | 277 | Sakamoto, K., Abe, T., Matsuyama, T., Yoshida, S., Ohmido, N., Fukui, K., et al. (2005). RAPID markers encoding retrotransposable elements are linked to the male sex in Cannabis sativa L. *GENOME* 48, 931–936. doi:10.1139/g05-056. |  |
| Cannabis flowering | 278 | Salentijn, E. M. J., Petit, J., and Trindade, L. M. (2019). The Complex Interactions Between Flowering Behavior and Fiber Quality in Hemp. *Front. Plant Sci.* 10. doi:10.3389/fpls.2019.00614. |  |
| Cannabis flowering | 279 | Salentijn, E. M. J., Zhang, Q., Amaducci, S., Yang, M., and Trindade, L. M. (2015). New developments in fiber hemp (Cannabis sativa L.) breeding. *Ind. Crops Prod.* 68, 32–41. doi:10.1016/j.indcrop.2014.08.011. |  |
| Cannabis flowering | 280 | Sandler, L. N., and Gibson, K. A. (2019). A call for weed research in industrial hemp (Cannabis sativa L). *WEED Res.* 59, 255–259. doi:10.1111/wre.12368. |  |
| Cannabis flowering | 281 | SARATH, G., and RAM, H. Y. M. (1979). COMPARATIVE EFFECT OF SILVER ION AND GIBBERELLIC-ACID ON THE INDUCTION OF MALE FLOWERS ON FEMALE CANNABIS PLANTS. *Experientia* 35, 333–334. doi:10.1007/BF01964334. |  |
| Cannabis flowering | 282 | Sarkar, S., Banerjee, J., and Gantait, S. (2017). Sex-oriented research on dioecious crops of Indian subcontinent: an updated review. *3 BIOTECH* 7. doi:10.1007/s13205-017-0723-8. |  |
| Cannabis flowering | 283 | Sausserde, R., and Adamovics, A. (2013). IMPACT OF NITROGEN FERTILIZER RATES ON INDUSTRIAL HEMP GROWTH AND DEVELOPMENT. in *RESEARCH FOR RURAL DEVELOPMENT 2013, VOL 1* Research for Rural Development., ed. Treija, S and Skujeniece, S, 50–55. |  |
| Cannabis flowering | 284 | Schilling, S., McCabe, P. F., and Melzer, R. (2020). Love is in the air: ethylene and sex determination in Cucurbita pepo. *J. Exp. Bot.* 71, 4–6. doi:10.1093/jxb/erz412. |  |
| Cannabis flowering | 285 | Schluttenhofer, C., and Yuan, L. (2017). Challenges towards Revitalizing Hemp: A Multifaceted Crop. *Trends Plant Sci.* 22, 917–929. doi:10.1016/j.tplants.2017.08.004. |  |
| Cannabis flowering | 286 | Seemann, B., Alon, T., Tsizin, S., Fialkov, A. B., and Amirav, A. (2015). Electron ionization LC-MS with supersonic molecular beams-the new concept, benefits and applications. *J. MASS Spectrom.* 50, 1252–1263. doi:10.1002/jms.3695. |  |
| Cannabis flowering | 287 | Serdar, U., Beyhan, N., Demirsoy, L., and Demirsoy, H. (2010). Some Phenological Properties of Chestnut Genotypes in the Black Sea Region, Turkey. in *I EUROPEAN CONGRESS ON CHESTNUT - CASTANEA 2009* Acta Horticulturae., ed. Bounous, G and Beccaro, GL, 135–142. |  |
| Cannabis flowering | 288 | Sexton, M., Shelton, K., Haley, P., and West, M. (2018). Evaluation of Cannabinoid and Terpenoid Content: Cannabis Flower Compared to Supercritical CO2 Concentrate (vol 84, pg 234, 2017). *Planta Med.* 84, E3. doi:10.1055/s-0043-122272. |  |
| Cannabis flowering | 289 | Shi, Y., Cao, Y., Shang, C., and Pacula, R. L. (2019). The impacts of potency, warning messages, and price on preferences for Cannabis flower products. *Int. J. DRUG POLICY* 74, 1–10. doi:10.1016/j.drugpo.2019.07.037. |  |
| Cannabis flowering | 290 | Singh, S. K., Richmond, M. D., Pearce, R. C., Bailey, W. A., Hou, X., Pattanaik, S., et al. (2020). Maleic hydrazide elicits global transcriptomic changes in chemically topped tobacco to influence shoot bud development. *Planta* 252. doi:10.1007/s00425-020-03460-9. |  |
| Cannabis flowering | 291 | Skaper, S. D., and Di Marzo, V. (2012). Endocannabinoids in nervous system health and disease: the big picture in a nutshell Introduction. *Philos. Trans. R. Soc. B-BIOLOGICAL Sci.* 367, 3193–3200. doi:10.1098/rstb.2012.0313. |  |
| Cannabis flowering | 292 | Small, E., and Naraine, S. G. U. (2016). Expansion of female sex organs in response to prolonged virginity in Cannabis sativa (marijuana). *Genet. Resour. Crop Evol.* 63, 339–348. doi:10.1007/s10722-015-0253-3. |  |
| Cannabis flowering | 293 | Smart, R., Caulkins, J. P., Kilmer, B., Davenport, S., and Midgette, G. (2017). Variation in cannabis potency and prices in a newly legal market: evidence from 30 million cannabis sales in Washington state. *ADDICTION* 112, 2167–2177. doi:10.1111/add.13886. |  |
| Cannabis flowering | 294 | Smeriglio, A., Trombetta, D., Alloisio, S., Cornara, L., Denaro, M., Garbati, P., et al. (2020). Promising in vitro antioxidant, anti-acetylcholinesterase and neuroactive effects of essential oil from two non-psychotropic Cannabis sativa L. biotypes. *Phyther. Res.* 34, 2287–2302. doi:10.1002/ptr.6678. |  |
| Cannabis flowering | 295 | Solano, J., Anabalon, L., Figueroa, A., and Gangitano, D. (2020). ITS barcoding using high resolution melting analysis of Cannabis sativa drug seizures in Chile: A forensic application. *FORENSIC Sci. Int.* 316. doi:10.1016/j.forsciint.2020.110550. |  |
| Cannabis flowering | 296 | Song, B. H., Wang, X. Q., Li, F. Z., and Hong, D. Y. (2001). Further evidence for paraphyly of the Celtidaceae from the chloroplast gene matK. *PLANT Syst. Evol.* 228, 107–115. doi:10.1007/s006060170041. |  |
| Cannabis flowering | 297 | Souleman, A. M. A., Gaafar, A. E.-D. M., Abdel-Salami, O. M., and ElShebiney, S. A. (2017). Determination of delta-9-tetrahydrocannabinol content of cannabis seizures in Egypt. *Asian Pac. J. Trop. Med.* 10, 291–294. doi:10.1016/j.apjtm.2017.03.011. |  |
| Cannabis flowering | 298 | Spikerman, D., Mandella, B., Hunt, R., and Riggle, J. (2017). Quantitation and characterization of anthocyanins in medical cannabis flowers cultivated in eastern Oregon. *Abstr. Pap. Am. Chem. Soc.* 253. |  |
| Cannabis flowering | 299 | Spindle, T. R., Bonn-Miller, M. O., and Vandrey, R. (2019). Changing landscape of cannabis: novel products, formulations, and methods of administration. *Curr. Opin. Psychol.* 30, 98–102. doi:10.1016/j.copsyc.2019.04.002. |  |
| Cannabis flowering | 300 | Spitzer-Rimon, B., Duchin, S., Bernstein, N., and Kamenetsky, R. (2019). Architecture and Florogenesis in Female Cannabis sativa Plants. *Front. Plant Sci.* 10. doi:10.3389/fpls.2019.00350. |  |
| Cannabis flowering | 301 | SRIRAM, N., and RAM, H. Y. M. (1984). SEX-ASSOCIATED DIFFERENCES IN PEROXIDASES AND ETHYLENE PRODUCTION AND THEIR MODIFICATION BY ETHEPHON TREATMENT IN THE FLOWERS OF CANNABIS-SATIVA L. *Curr. Sci.* 53, 735–739. |  |
| Cannabis flowering | 302 | Stastnik, O., Karasek, F., Stenclova, H., Burdova, E., Kalhotka, L., Trojan, V., et al. (2016). THE EFFECT OF HEMP BY-PRODUCTS FEEDING ON GUT MICROBIOTA AND GROWTH OF BROILER CHICKENS. in *PROCEEDINGS OF INTERNATIONAL PHD STUDENTS CONFERENCE, (MENDELNET 2016)*, ed. Polak, O and Cerkal, R and Belcredi, NB and Horky, P and Vacek, P, 289–293. |  |
| Cannabis flowering | 303 | Stith, S. S., Diviant, J. P., Brockelman, F., Keeling, K., Hall, B., Lucern, S., et al. (2020). Alleviative effects of Cannabis flower on migraine and headache. *J. Integr. Med.* 18, 416–424. doi:10.1016/j.joim.2020.07.004. |  |
| Cannabis flowering | 304 | Stith, S. S., Vigil, J. M., Brockelman, F., Keeling, K., and Hall, B. (2019). The Association between Cannabis Product Characteristics and Symptom Relief. *Sci. Rep.* 9. doi:10.1038/s41598-019-39462-1. |  |
| Cannabis flowering | 305 | Stolze, N., Bader, C., Henning, C., Mastin, J., Holmes, A. E., and Sutlief, A. L. (2019a). Automated image analysis with ImageJ of yeast colony forming units from cannabis flowers. *J. Microbiol. Methods* 164. doi:10.1016/j.mimet.2019.105681. |  |
| Cannabis flowering | 306 | Stolze, N., Sutlief, A., and Holmes, A. (2019b). Using ImageJ for automated counting of colony forming units of yeast and molds in cannabis flowers. *Abstr. Pap. Am. Chem. Soc.* 257. |  |
| Cannabis flowering | 307 | Stonehouse, G. C., McCarron, B. J., Guignardi, Z. S., El Mehdawi, A. F., Lima, L. W., Fakra, S. C., et al. (2020). Selenium Metabolism in Hemp (Cannabis sativa L.)-Potential for Phytoremediation and Biofortification. *Environ. Sci. Technol.* 54, 4221–4230. doi:10.1021/acs.est.9b07747. |  |
| Cannabis flowering | 308 | Stout, J. M., Boubakir, Z., Ambrose, S. J., Purves, R. W., and Page, J. E. (2012). The hexanoyl-CoA precursor for cannabinoid biosynthesis is formed by an acyl-activating enzyme in Cannabis sativa trichomes. *PLANT J.* 71, 353–365. doi:10.1111/j.1365-313X.2012.04949.x. |  |
| Cannabis flowering | 309 | Struik, P. C., Amaducci, S., Bullard, M. J., Stutterheim, N. C., Venturi, G., and Cromack, H. T. H. (2000). Agronomy of fibre hemp (Cannabis sativa L.) in Europe. *Ind. Crops Prod.* 11, 107–118. doi:10.1016/S0926-6690(99)00048-5. |  |
| Cannabis flowering | 310 | Sun, X., and Dey, S. K. (2012). Endocannabinoid Signaling in Female Reproduction. *ACS Chem. Neurosci.* 3, 349–355. doi:10.1021/cn300014e. |  |
| Cannabis flowering | 311 | Swift, W., Hall, W., and Copeland, J. (1998). Characteristics of long-term cannabis users in Sydney, Australia. *Eur. Addict. Res.* 4, 190–197. doi:10.1159/000018952. |  |
| Cannabis flowering | 312 | Szejko, N., Fremer, C., and Mueller-Vahl, K. R. (2020). Cannabis Improves Obsessive-Compulsive Disorder-Case Report and Review of the Literature. *Front. PSYCHIATRY* 11. doi:10.3389/fpsyt.2020.00681. |  |
| Cannabis flowering | 313 | Tang, K., Struik, P. C., Yin, X., Thouminot, C., Bjelkova, M., Stramkale, V., et al. (2016). Comparing hemp (Cannabis sativa L.) cultivars for dual-purpose production under contrasting environments. *Ind. Crops Prod.* 87, 33–44. doi:10.1016/j.indcrop.2016.04.026. |  |
| Cannabis flowering | 314 | Taura, F., Iijima, M., Lee, J.-B., Hashimoto, T., Asakawa, Y., and Kurosaki, F. (2014). Daurichromenic Acid-producing Oxidocyclase in the Young Leaves of Rhododendron dauricum. *Nat. Prod. Commun.* 9, 1329–1332. |  |
| Cannabis flowering | 315 | Taura, F., Tanaka, S., Taguchi, C., Fukamizu, T., Tanaka, H., Shoyama, Y., et al. (2009). Characterization of olivetol synthase, a polyketide synthase putatively involved in cannabinoid biosynthetic pathway. *FEBS Lett.* 583, 2061–2066. doi:10.1016/j.febslet.2009.05.024. |  |
| Cannabis flowering | 316 | Techen, N., Chandra, S., Lata, H., ElSohly, M. A., and Khan, I. A. (2010). Genetic Identification of Female Cannabis sativa Plants at Early Developmental Stage. *Planta Med.* 76, 1938–1939. doi:10.1055/s-0030-1249978. |  |
| Cannabis flowering | 317 | Tellez-Zenteno, J. F., Ladino, L. D., and Hernandez-Ronquillo, L. (2020). The Use of Cannabis as a Treatment for Epilepsy in Adult Patients: Are Side Effects a Limitation of Use? *J. Clin. Neurophysiol.* 37, 9–14. doi:10.1097/WNP.0000000000000637. |  |
| Cannabis flowering | 318 | Thiessen, L. D., Schappe, T., Cochran, S., Hicks, K., and Post, A. R. (2020). Surveying for Potential Diseases and Abiotic Disorders of Industrial Hemp (Cannabis sativa) Production. *PLANT Heal. Prog.* 21, 321–332. doi:10.1094/PHP-03-20-0017-RS. |  |
| Cannabis flowering | 319 | Toonen, M., Ribot, S., and Thissen, J. (2006). Yield of Illicit Indoor Cannabis Cultivation in The Netherlands. *J. Forensic Sci.* 51, 1050–1054. doi:10.1111/j.1556-4029.2006.00228.x. |  |
| Cannabis flowering | 320 | Tose, L. V, Santos, N. A., Rodrigues, R. R. T., Murgu, M., Gomes, A. F., Vasconcelos, G. A., et al. (2017). Isomeric separation of cannabinoids by UPLC combined with ionic mobility mass spectrometry (TWIM-MS) Part I. *Int. J. Mass Spectrom.* 418, 112–121. doi:10.1016/j.ijms.2016.10.018. |  |
| Cannabis flowering | 321 | Turgeman, I., and Bar-Sela, G. (2019). Cannabis for cancer - illusion or the tip of an iceberg: a review of the evidence for the use of Cannabis and synthetic cannabinoids in oncology. *Expert Opin. Investig. Drugs* 28, 285–296. doi:10.1080/13543784.2019.1561859. |  |
| Cannabis flowering | 322 | Turgeman, L., and Bar -Seta, G. (2017). Cannabis Use in Palliative Oncology: A Review of the Evidence for Popular Indicatios. *Isr. Med. Assoc. J.* 19, 85–88. |  |
| Cannabis flowering | 323 | Ueno, L. F., Mian, M. N., Altman, B. R., Giandelone, E., Luce, M., and Earleywine, M. Age-Related Differences in Cannabis Product Use. *J. Psychoactive Drugs*. doi:10.1080/02791072.2020.1870778. |  |
| Cannabis flowering | 324 | Valdes-Donoso, P., Sumner, D. A., and Goldstein, R. (2020). Costs of cannabis testing compliance: Assessing mandatory testing in the California cannabis market. *PLoS One* 15. doi:10.1371/journal.pone.0232041. |  |
| Cannabis flowering | 325 | VanderWerf, H. M. G., Mathijssen, E., and Haverkort, A. J. (1996). The potential of hemp (Cannabis sativa L) for sustainable fibre production: A crop physiological appraisal. *Ann. Appl. Biol.* 129, 109–123. |  |
| Cannabis flowering | 326 | VANDERWERF, H. M. G., WIJLHUIZEN, M., and DESCHUTTER, J. A. A. (1995). PLANT-DENSITY AND SELF-THINNING AFFECT YIELD AND QUALITY OF FIBER HEMP (CANNABIS-SATIVA L). *F. Crop. Res.* 40, 153–164. doi:10.1016/0378-4290(94)00103-J. |  |
| Cannabis flowering | 327 | Vanhoenacker, G., Van Rompaey, P., De Keukeleire, D., and Sandra, P. (2002). Chemotaxonomic features associated with flavonoids of cannabinoid-free cannabis (Cannabis sativa subsp sativa L.) in relation to hops (Humulus lupulus L.). *Nat. Prod. Lett.* 16, 57–63. doi:10.1080/1057563029001/4863. |  |
| Cannabis flowering | 328 | Vuerich, M., Ferfuia, C., Zuliani, F., Piani, B., Sepulcri, A., and Baldini, M. (2019). Yield and Quality of Essential Oils in Hemp Varieties in Different Environments. *AGRONOMY-BASEL* 9. doi:10.3390/agronomy9070356. |  |
| Cannabis flowering | 329 | Walsh, D., Nelson, K. A., and Mahmoud, F. A. (2003). Established and potential therapeutic applications of cannabinoids in oncology. *Support. CARE CANCER* 11, 137–143. doi:10.1007/s00520-002-0387-7. |  |
| Cannabis flowering | 330 | Wang, Y.-H., Avula, B., ElSohly, M. A., Radwan, M. M., Wang, M., Wanas, A. S., et al. (2018). Quantitative Determination of (9) -THC, CBG, CBD, Their Acid Precursors and Five Other Neutral Cannabinoids by UHPLC-UV-MS. *Planta Med.* 84, 260–266. doi:10.1055/s-0043-124873. |  |
| Cannabis flowering | 331 | Welling, M. T., Liu, L., Raymond, C. A., Ansari, O., and King, G. J. (2018). Developmental Plasticity of the Major Alkyl Cannabinoid Chemotypes in a Diverse Cannabis Genetic Resource Collection. *Front. Plant Sci.* 9. doi:10.3389/fpls.2018.01510. |  |
| Cannabis flowering | 332 | Wendelmuth, C., and Gastmeier, K. (2019). High-dose dronabinol treatment vs. medicinal cannabis flowers. *SCHMERZ* 33, 392–398. doi:10.1007/s00482-019-00402-7. |  |
| Cannabis flowering | 333 | Westerhuis, W., Struik, P. C., van Dam, J. E. G., and Stomph, T. J. (2009). Postponed sowing does not alter the fibre/wood ratio or fibre extractability of fibre hemp (Cannabis sativa). *Ann. Appl. Biol.* 155, 333–348. doi:10.1111/j.1744-7348.2009.00342.x. |  |
| Cannabis flowering | 334 | Westerhuis, W., van Delden, S. H., van Dam, J. E. G., Marinho, J. P. P., Struik, P. C., and Stomph, T. J. (2019). Plant weight determines secondary fibre development in fibre hemp (Cannabis sativa L.). *Ind. Crops Prod.* 139. doi:10.1016/j.indcrop.2019.111493. |  |
| Cannabis flowering | 335 | Wilcox, J., Pazdanska, M., Milligan, C., Chan, D., MacDonald, S. J., and Donnelly, C. (2020). Analysis of Aflatoxins and Ochratoxin A in Cannabis and Cannabis Products by LC-Fluorescence Detection Using Cleanup with Either Multiantibody Immunoaffinity Columns or an Automated System with In-Line Reusable Immunoaffinity Cartridges. *J. AOAC Int.* 103, 494–503. doi:10.5740/jaoacint.19-0176. |  |
| Cannabis flowering | 336 | Yang, R., Berthold, E. C., McCurdy, C. R., Benevenute, S. da S., Brym, Z. T., and Freeman, J. H. (2020). Development of Cannabinoids in Flowers of Industrial Hemp (Cannabis sativa L.): A Pilot Study. *J. Agric. Food Chem.* 68, 6058–6064. doi:10.1021/acs.jafc.0c01211. |  |
| Cannabis flowering | 337 | Yep, B., Gale V, N., and Zheng, Y. (2020a). Aquaponic and Hydroponic Solutions Modulate NaCl-Induced Stress in Drug-Type Cannabis sativa L. *Front. Plant Sci.* 11. doi:10.3389/fpls.2020.01169. |  |
| Cannabis flowering | 338 | Yep, B., Gale V, N., and Zheng, Y. (2020b). Comparing hydroponic and aquaponic rootzones on the growth of two drug-type Cannabis sativa L. cultivars during the flowering stage. *Ind. Crops Prod.* 157. doi:10.1016/j.indcrop.2020.112881. |  |
| Cannabis flowering | 339 | Zager, J. J., Lange, I., Srividya, N., Smith, A., and Lange, B. M. (2019). Gene Networks Underlying Cannabinoid and Terpenoid Accumulation in Cannabis. *PLANT Physiol.* 180, 1877–1897. doi:10.1104/pp.18.01506. |  |
| Cannabis flowering | 340 | Zain, N. A. B. M., Zin, N. A. B. M., Mahmud, N. F. B., Isnin, N., and Zain, N. B. M. (2016). The Role of Malaysian Non-Government Organization in Promoting Medical Marijuana. *Adv. Sci. Lett.* 22, 4181–4184. doi:10.1166/as1.2016.8094. |  |
| Cannabis flowering | 341 | Zenginbal, H. (2017). A COMPARISON OF PROFESSIONAL AND NON-PROFESSIONAL TYING AND WRAPPING MATERIALS ON GRAFTING SUCCESS OF JAPANESE FLOWERING CRABAPPLE. *Propag. Ornam. PLANTS* 17, 12–19 |  |
| Photoperiodic hemp | 342 | BORTHWICK, H. A., and SCULLY, N. J. (1954). PHOTOPERIODIC RESPONSES OF HEMP. *Bot. Gaz.* 116, 14–29. doi:10.1086/335843. |  |
| Photoperiodic hemp | 343 | Hall, J., Bhattarai, S. P., and Midmore, D. J. (2012). Review of Flowering Control in Industrial Hemp. *J. Nat. FIBERS* 9, 23–36. doi:10.1080/15440478.2012.651848. |  |
| Cannabis lighting | 344 | [Anonymous] (1999). Cannabis trials get the green light. *Chem. Ind.*, 44. |  |
| Cannabis lighting | 345 | Abdollahi, M., Sefidkon, F., Calagari, M., Mousavi, A., and Mahomoodally, M. F. (2020). A comparative study of seed yield and oil composition of four cultivars of Hemp (Cannabis sativa L.) grown from three regions in northern Iran. *Ind. Crops Prod.* 152. doi:10.1016/j.indcrop.2020.112397. |  |
| Cannabis lighting | 346 | Abu-Amna, M., Salti, T., Khoury, M., Cohen, I., and Bar-Sela, G. (2021). Medical Cannabis in Oncology: a Valuable Unappreciated Remedy or an Undesirable Risk? *Curr. Treat. Options Oncol.* 22. doi:10.1007/s11864-020-00811-2. |  |
| Cannabis lighting | 347 | Adda, J., McConnell, B., and Rasul, I. (2014). Crime and the Depenalization of Cannabis Possession: Evidence from a Policing Experiment. *J. Polit. Econ.* 122, 1130–1202. doi:10.1086/676932. |  |
| Cannabis lighting | 348 | Adrian, M. (2015). What the History of Drugs Can Teach Us About the Current Cannabis Legalization Process: Unfinished Business. *Subst. Use Misuse* 50, 990–1004. doi:10.3109/10826084.2015.1013725. |  |
| Cannabis lighting | 349 | Afsahi, K., and Darwich, S. (2016). Hashish in Morocco and Lebanon: A comparative study. *Int. J. DRUG POLICY* 31, 190–198. doi:10.1016/j.drugpo.2016.02.024. |  |
| Cannabis lighting | 350 | Aigner, M., Treasure, J., Kaye, W., Kasper, S., and Disorders, W. T. F. E. (2011). World Federation of Societies of Biological Psychiatry (WFSBP) Guidelines for the Pharmacological Treatment of Eating Disorders. *WORLD J. Biol. PSYCHIATRY* 12, 400–443. doi:10.3109/15622975.2011.602720. |  |
| Cannabis lighting | 351 | Akano, O. F. (2017). Marijuana Use and Self-reported Quality of Eyesight. *Optom. Vis. Sci.* 94, 630–633. doi:10.1097/OPX.0000000000001069. |  |
| Cannabis lighting | 352 | Akyeampong, E. (2005). Diaspora and drug trafficking in West Africa: A case study of Ghana. *Afr. Aff. (Lond).* 104, 429–447. doi:10.1093/afraf/adi015. |  |
| Cannabis lighting | 353 | Alshenguity, A. A., Alruhaili, E. M. S., Alreheli, A. Q., AlQaidi, S. H., Almuzaini, R. A., and Karbouji, M. A. (2019). SOCIO-DEMOGRAPHIC CORRELATES AND PATTERNS OF USE OF CAPTAGON USERS VISITING ALAMAL MENTAL HEALTH COMPLEX IN ALMADINAH. *INDO Am. J. Pharm. Sci.* 6, 1283–1291. doi:10.5281/zenodo.2543401. |  |
| Cannabis lighting | 354 | Altamura, A. C., Delvecchio, G., Marotta, G., Oldani, L., Pigoni, A., Ciappolino, V., et al. (2017). Structural and metabolic differentiation between bipolar disorder with psychosis and substance-induced psychosis: An integrated MRI/PET study. *Eur. PSYCHIATRY* 41, 85–94. doi:10.1016/j.eurpsy.2016.09.009. |  |
| Cannabis lighting | 355 | Amatullah, S., Kandasamy, A., Benegal, V., and Narayanan, G. (2020). Exploring identity, culture, and psychosis in cannabis dependence - an interpretative phenomenological case study from India. *Ment. Heal. Relig. \& Cult.* 23, 347–362. doi:10.1080/13674676.2020.1737920. |  |
| Cannabis lighting | 356 | Ameller, A., and Gorwood, P. (2015). Attributable risk of co-morbid substance use disorder in poor Observance to pharmacological treatment and the occurrence of relapse in schizophrenia. *Enceph. Psychiatr. Clin. Biol. Ther.* 41, 174–183. doi:10.1016/j.encep.2015.02.002. |  |
| Cannabis lighting | 357 | Amirav, I., Luder, A., Viner, Y., and Finkel, M. (2011). Decriminalization of Cannabis - potential risks for children? *ACTA Paediatr.* 100, 618–619. doi:10.1111/j.1651-2227.2010.02081.x. |  |
| Cannabis lighting | 358 | Ancrum, C., and Treadwell, J. (2017). Beyond ghosts, gangs and good sorts: Commercial cannabis cultivation and illicit enterprise in England’s disadvantaged inner cities. *CRIME MEDIA Cult.* 13, 69–84. doi:10.1177/1741659016646414. |  |
| Cannabis lighting | 359 | Andre, C. M., Hausman, J. F., and Guerriero, G. (2016). Cannabis sativa: The plant of the thousand and one molecules. *Front. Plant Sci.* 7. doi:10.3389/fpls.2016.00019. |  |
| Cannabis lighting | 360 | Arie, E., Rosen, B., and Namdar, D. (2020). Cannabis and Frankincense at the Judahite Shrine of Arad. *TEL AVIV-JOURNAL Inst. Archaeol. TEL AVIV Univ.* 47, 5–28. doi:10.1080/03344355.2020.1732046. |  |
| Cannabis lighting | 361 | Arkell, T. R., Lintzeris, N., Kevin, R. C., Ramaekers, J. G., Vandrey, R., Irwin, C., et al. (2019). Cannabidiol (CBD) content in vaporized cannabis does not prevent tetrahydrocannabinol (THC)-induced impairment of driving and cognition. *Psychopharmacology (Berl).* 236, 2713–2724. doi:10.1007/s00213-019-05246-8. |  |
| Cannabis lighting | 362 | Armour, M., Sinclair, J., Chalmers, K. J., and Smith, C. A. (2019). Self-management strategies amongst Australian women with endometriosis: a national online survey. *BMC Complement. Altern. Med.* 19. doi:10.1186/s12906-019-2431-x. |  |
| Cannabis lighting | 363 | Armstrong, K. A., Watling, C. N., and Davey, J. D. (2018). Deterrence of drug driving: The impact of the ACT drug driving legislation and detection techniques. *Transp. Res. PART F-TRAFFIC Psychol. Behav.* 54, 138–147. doi:10.1016/j.trf.2018.01.014. |  |
| Cannabis lighting | 364 | Assirelli, A., Dal Re, L., Esposito, S., Cocchi, A., and Santangelo, E. (2020). The Mechanical Harvesting of Hemp Using In-Field Stand-Retting: A Simpler Approach Converted to the Production of Fibers for Industrial Use. *SUSTAINABILITY* 12. doi:10.3390/su12218795. |  |
| Cannabis lighting | 365 | Auger, N., Rheaume, M.-A., Low, N., Lee, G. E., Ayoub, A., and Luu, T. M. (2020). Impact of Prenatal Exposure to Opioids, Cocaine, and Cannabis on Eye Disorders in Children. *J. Addict. Med.* 14, 459–466. doi:10.1097/ADM.0000000000000621. |  |
| Cannabis lighting | 366 | Babson, K. A., Boden, M. T., and Bonn-Miller, M. O. (2013). The impact of perceived sleep quality and sleep efficiency/duration on cannabis use during a self-guided quit attempt. *Addict. Behav.* 38, 2707–2713. doi:10.1016/j.addbeh.2013.06.012. |  |
| Cannabis lighting | 367 | Backer, R., Schwinghamer, T., Rosenbaum, P., McCarty, V., Eichhorn Bilodeau, S., Lyu, D., et al. (2019). Closing the yield gap for cannabis: A meta-analysis of factors determining cannabis yield. *Front. Plant Sci.* 10, 495. doi:10.3389/fpls.2019.00495. |  |
| Cannabis lighting | 368 | Bahorik, A. L., Leibowitz, A., Sterling, S. A., Travis, A., Weisner, C., and Satre, D. D. (2017). Patterns of marijuana use among psychiatry patients with depression and its impact on recovery. *J. Affect. Disord.* 213, 168–171. doi:10.1016/j.jad.2017.02.016. |  |
| Cannabis lighting | 369 | Bailey, J. A., Hill, K. G., Guttmannova, K., Epstein, M., Abbott, R. D., Steeger, C. M., et al. (2016). Associations Between Parental and Grandparental Marijuana Use and Child Substance Use Norms in a Prospective, Three-Generation Study. *J. Adolesc. Heal.* 59, 262–268. doi:10.1016/j.jadohealth.2016.04.010. |  |
| Cannabis lighting | 370 | Bakdash, A., AL-Mathloum, A. M. K., ElAmin, E. H. A., Abu Taha, N. M. T., Kumar, S., and Nasr, F. A. (2018). Single-dose acute toxicity of THJ-2201 designer Cannabis drug: LD50 and hematological and histological changes in mice. *Egypt. J. FORENSIC Sci.* 8. doi:10.1186/s41935-018-0079-1. |  |
| Cannabis lighting | 371 | Baker, D., and Pryce, G. (2003). The therapeutic potential of cannabis in multiple sclerosis. *Expert Opin. Investig. Drugs* 12, 561–567. |  |
| Cannabis lighting | 372 | Barrantes-Vidal, N., Grant, P., and Kwapil, T. R. (2015). The Role of Schizotypy in the Study of the Etiology of Schizophrenia Spectrum Disorders. *Schizophr. Bull.* 41, S408–S416. doi:10.1093/schbul/sbu191. |  |
| Cannabis lighting | 373 | Barre, T., Nishimwe, M. L., Protopopescu, C., Marcellin, F., Carrat, F., Dorival, C., et al. (2020). Cannabis use is associated with a lower risk of diabetes in chronic hepatitis C-infected patients (ANRS CO22 Hepather cohort). *J. Viral Hepat.* 27, 1473–1483. doi:10.1111/jvh.13380. |  |
| Cannabis lighting | 374 | Barrett, D. (2019). Canada, cannabis and the relationship between UN child rights and drug control treaties. *Int. J. DRUG POLICY* 71, 29–35. doi:10.1016/j.drugpo.2019.02.010. |  |
| Cannabis lighting | 375 | Barrington-Trimis, J. L., Cho, J., Ewusi-Boisvert, E., Hasin, D., Unger, J. B., Miech, R. A., et al. (2020). Risk of Persistence and Progression of Use of 5 Cannabis Products After Experimentation Among Adolescents. *JAMA Netw. OPEN* 3. doi:10.1001/jamanetworkopen.2019.19792. |  |
| Cannabis lighting | 376 | Bauerle, W. L., McCullough, C., Iversen, M., and Hazlett, M. (2020). Leaf Age and Position Effects on Quantum Yield and Photosynthetic Capacity in Hemp Crowns. *PLANTS-BASEL* 9. doi:10.3390/plants9020271. |  |
| Cannabis lighting | 377 | Beal, K. (2019). Considerations in the addition of cannabis to chocolate. *Curr. Opin. FOOD Sci.* 28, 14–17. doi:10.1016/j.cofs.2019.02.007. |  |
| Cannabis lighting | 378 | Bears Augustyn, M., Loughran, T., Larroulet, P., and Henry, K. L. (2020). Economic Effects of Adolescent to Adult Patterns of Cannabis Use: Full-Time Employment and Employment Stability. *J. Drug Issues* 50, 579–603. doi:10.1177/0022042620943538. |  |
| Cannabis lighting | 379 | Bedrouni, W. (2018). On the use of digital technologies to reduce the public health impacts of cannabis legalization in Canada. *Can. J. PUBLIC Heal. Can. SANTE PUBLIQUE* 109, 748–751. doi:10.17269/s41997-018-0117-7. |  |
| Cannabis lighting | 380 | Behnood, A., and Mannering, F. (2017). The effects of drug and alcohol consumption on driver injury severities in single-vehicle crashes. *TRAFFIC Inj. Prev.* 18, 456–462. doi:10.1080/15389588.2016.1262540. |  |
| Cannabis lighting | 381 | Behr, M., Legay, S., Zizkova, E., Motyka, V., Dobrev, P. I., Hausman, J.-F., et al. (2016). Studying Secondary Growth and Bast Fiber Development: The Hemp Hypocotyl Peeks behind the Wall. *Front. Plant Sci.* 7. doi:10.3389/fpls.2016.01733. |  |
| Cannabis lighting | 382 | Belhassen, Y., and Shani, A. (2012). Hotel workers’ substance use and abuse. *Int. J. Hosp. Manag.* 31, 1292–1302. doi:10.1016/j.ijhm.2012.03.011. |  |
| Cannabis lighting | 383 | Benelli, G., Pavela, R., Lupidi, G., Nabissi, M., Petrelli, R., Kamte, S. L. N., et al. (2018a). The crop-residue of fiber hemp cv. Futura 75: from a waste product to a source of botanical insecticides. *Environ. Sci. Pollut. Res.* 25, 10515–10525. doi:10.1007/s11356-017-0635-5. |  |
| Cannabis lighting | 384 | Benelli, G., Pavela, R., Petrelli, R., Cappellacci, L., Santini, G., Fiorini, D., et al. (2018b). The essential oil from industrial hemp (Cannabis sativa L.) by-products as an effective tool for insect pest management in organic crops. *Ind. Crops Prod.* 122, 308–315. doi:10.1016/j.indcrop.2018.05.032. |  |
| Cannabis lighting | 385 | Bentzley, J. P., Tomko, R. L., and Gray, K. M. (2016). Low Pretreatment Impulsivity and High Medication Adherence Increase the Odds of Abstinence in a Trial of N-Acetylcysteine in Adolescents with Cannabis Use Disorder. *J. Subst. Abuse Treat.* 63, 72–77. doi:10.1016/j.jsat.2015.12.003. |  |
| Cannabis lighting | 386 | Berlowitz, I., Walt, H., Ghasarian, C., O’Shaughnessy, D. M., Mabit, J., Rush, B., et al. (2020). Who Turns to Amazonian Medicine for Treatment of Substance Use Disorder? Patient Characteristics at the Takiwasi Addiction Treatment Center. *J. Stud. Alcohol Drugs* 81, 416–425. |  |
| Cannabis lighting | 387 | Bersani, F. S., Santacroce, R., Coviello, M., Imperatori, C., Francesconi, M., Vicinanza, R., et al. (2016). Cannabis: a self-medication drug for weight management? The never ending story. *DRUG Test. Anal.* 8, 177–179. doi:10.1002/dta.1891. |  |
| Cannabis lighting | 388 | Bilodeau, S. E., Wu, B.-S., Rufyikiri, A.-S., MacPherson, S., and Lefsrud, M. (2019). An Update on Plant Photobiology and Implications for Cannabis Production. *Front. Plant Sci.* 10. doi:10.3389/fpls.2019.00296. |  |
| Cannabis lighting | 389 | Blecha, L., Benyamina, A., and Reynaud, M. (2010). Family management of cannabis in adolescent. *Arch. Pediatr.* 17, 191–194. doi:10.1016/j.arcped.2009.09.018. |  |
| Cannabis lighting | 390 | BLOCK, R. I., and GHONEIM, M. M. (1993). EFFECTS OF CHRONIC MARIJUANA USE ON HUMAN COGNITION. *Psychopharmacology (Berl).* 110, 219–228. doi:10.1007/BF02246977. |  |
| Cannabis lighting | 391 | Boccio, C. M., and Beaver, K. M. (2017). Examining the influence of adolescent marijuana use on adult intelligence: Further evidence in the causation versus spuriousness debate. *Drug Alcohol Depend.* 177, 199–206. doi:10.1016/j.drugalcdep.2017.04.007. |  |
| Cannabis lighting | 392 | Bocker, K. B. E., Gerritsen, J., Hunault, C. C., Kruidenier, M., Mensinga, T. T., and Kenemans, J. L. (2010). Cannabis with high Delta(9)-THC contents affects perception and visual selective attention acutely: An event-related potential study. *Pharmacol. Biochem. Behav.* 96, 67–74. doi:10.1016/j.pbb.2010.04.008. |  |
| Cannabis lighting | 393 | Boehnke, K. F., Scott, J. R., Litinas, E., Sisley, S., Williams, D. A., and Clauw, D. J. (2020). High-Frequency Medical Cannabis Use Is Associated With Worse Pain Among Individuals With Chronic Pain. *J. PAIN* 21, 570–581. doi:10.1016/j.jpain.2019.09.006. |  |
| Cannabis lighting | 394 | Bolhuis, K., Kushner, S. A., Yalniz, S., Hillegers, M. H. J., Jaddoe, V. W. V, Tiemeier, H., et al. (2018). Maternal and paternal cannabis use during pregnancy and the risk of psychotic-like experiences in the offspring. *Schizophr. Res.* 202, 322–327. doi:10.1016/j.schres.2018.06.067. |  |
| Cannabis lighting | 395 | Bolla, K. I., Brown, K., Eldreth, D., Tate, K., and Cadet, J. L. (2002). Dose-related neurocognitive effects of marijuana use. *Neurology* 59, 1337–1343. doi:10.1212/01.WNL.0000031422.66442.49. |  |
| Cannabis lighting | 396 | Bolognini, M., Plancherel, B., Laget, J., and Halfon, O. (2003). Adolescent’s suicide attempts: Populations at risk, vulnerability, and substance use. *Subst. Use Misuse* 38, 1651–1669. doi:10.1081/JA-120024235. |  |
| Cannabis lighting | 397 | Bonnet, U. (2016). Abrupt Quitting of Long-term Heavy Recreational Cannabis Use is Not Followed by Significant Changes in Blood Pressure and Heart Rate. *Pharmacopsychiatry* 49, 23–25. doi:10.1055/s-0035-1565242. |  |
| Cannabis lighting | 398 | Bonnet, U., Canbay, A., Specka, M., and Scherbaum, N. (2018). Long-Term Heavy Recreational Cannabis Use and Serum Delta-9-Tetrahydrocannabinol Levels are not Associated with an Impaired Liver Function in Cannabis Dependents. *J. Psychoactive Drugs* 50, 355–360. doi:10.1080/02791072.2018.1482031. |  |
| Cannabis lighting | 399 | Bonnet, U., and Preuss, U. W. (2017). The cannabis withdrawal syndrome: current insights. *Subst. Abuse Rehabil.* 8, 9–37. doi:10.2147/SAR.S109576. |  |
| Cannabis lighting | 400 | Bonny-Noach, H. (2019). Harm reduction drug policy in Israel: what has been accomplished and what still needs to be done? *Isr. J. Health Policy Res.* 8. doi:10.1186/s13584-019-0343-3. |  |
| Cannabis lighting | 401 | Boucher, A. A., Arnold, J. C., Duffy, L., Schofield, P. R., Micheau, J., and Karl, T. (2007). Heterozygous neuregulin 1 mice are more sensitive to the behavioural effects of Delta(9)-tetrahydrocannabinol. *Psychopharmacology (Berl).* 192, 325–336. doi:10.1007/s00213-007-0721-3. |  |
| Cannabis lighting | 402 | Boucher, A. A., Hunt, G. E., Micheau, J., Huang, X., McGregor, I. S., Karl, T., et al. (2011). The schizophrenia susceptibility gene neuregulin 1 modulates tolerance to the effects of cannabinoids. *Int. J. Neuropsychopharmacol.* 14, 631–643. doi:10.1017/S146114571000091X. |  |
| Cannabis lighting | 403 | Boumrah, Y., Baroudi, S., Kecir, M., and Bouanani, S. (2020). Characterization of Algerian-Seized Hashish Over Eight Years (2011-2018). Part I: Physical Categorization. *J. Forensic Sci.* 65, 1835–1844. doi:10.1111/1556-4029.14534. |  |
| Cannabis lighting | 404 | Bourne, D., Plinke, W., Hooker, E. R., and Nielson, C. M. (2017). Cannabis use and bone mineral density: NHANES 2007-2010. *Arch. Osteoporos.* 12. doi:10.1007/s11657-017-0320-9. |  |
| Cannabis lighting | 405 | Bramness, J. G., Khiabani, H. Z., and Morland, J. (2010). Impairment due to cannabis and ethanol: clinical signs and additive effects. *ADDICTION* 105, 1080–1087. doi:10.1111/j.1360-0443.2010.02911.x. |  |
| Cannabis lighting | 406 | Brand, E. J., and Zhao, Z. (2017). Cannabis in Chinese Medicine: Are Some Traditional Indications Referenced in Ancient Literature Related to Cannabinoids? *Front. Pharmacol.* 8. doi:10.3389/fphar.2017.00108. |  |
| Cannabis lighting | 407 | Breitbarth, A. K., Morgan, J., and Jones, A. L. (2018). E-cigarettes-An unintended illicit drug delivery system. *Drug Alcohol Depend.* 192, 98–111. doi:10.1016/j.drugalcdep.2018.07.031. |  |
| Cannabis lighting | 408 | Brenneisen, R., Meyer, P., Chtioui, H., Saugy, M., and Kamber, M. (2010). Plasma and urine profiles of Delta(9)-tetrahydrocannabinol and its metabolites 11-hydroxy-Delta(9)-tetrahydrocannabinol and 11-nor-9-carboxy-Delta(9)-tetrahydrocannabinol after cannabis smoking by male volunteers to estimate recent consumption by athletes. *Anal. Bioanal. Chem.* 396, 2493–2502. doi:10.1007/s00216-009-3431-3. |  |
| Cannabis lighting | 409 | Bressloff, P. C., Cowan, J. D., Golubitsky, M., Thomas, P. J., and Wiener, M. C. (2002). What geometric visual hallucinations tell us about the visual cortex. *NEURAL Comput.* 14, 473–491. doi:10.1162/089976602317250861. |  |
| Cannabis lighting | 410 | Brewerton, T. D., and Anderson, O. Cannabinoid Hyperemesis Syndrome Masquerading as an Eating Disorder. doi:10.1002/eat.22515. |  |
| Cannabis lighting | 411 | Brighenti, V., Licata, M., Pedrazzi, T., Maran, D., Bertelli, D., Pellati, F., et al. (2019). Development of a new method for the analysis of cannabinoids in honey by means of high-performance liquid chromatography coupled with electrospray ionisation-tandem mass spectrometry detection. *J. Chromatogr. A* 1597, 179–186. doi:10.1016/j.chroma.2019.03.034. |  |
| Cannabis lighting | 412 | Brogan, A. P., Eubanks, L. M., Koob, G. F., Dickerson, T. J., and Janda, K. D. (2007). Antibody-catalyzed oxidation of Delta(9)-tetrahydrocannabinol. *J. Am. Chem. Soc.* 129, 3698–3702. doi:10.1021/ja070022m. |  |
| Cannabis lighting | 413 | Bron, T. I., Bijlenga, D., Kasander, M. V, Spuijbroek, A. T., Beekman, A. T. F., and Kooij, J. J. S. (2013). Long-term relationship between methylphenidate and tobacco consumption and nicotine craving in adults with ADHD in a prospective cohort study. *Eur. Neuropsychopharmacol.* 23, 542–554. doi:10.1016/j.euroneuro.2012.06.004. |  |
| Cannabis lighting | 414 | Brooks, S. J., Funk, S. G., Young, S. Y., and Schioth, H. B. (2017). The Role of Working Memory for Cognitive Control in Anorexia Nervosa versus Substance Use Disorder. *Front. Psychol.* 8. doi:10.3389/fpsyg.2017.01651. |  |
| Cannabis lighting | 415 | Brousse, G., Geneste-Saelens, J., Cabe, J., and Cottencin, O. (2018). Alcohol and emergencies. *Press. MEDICALE* 47, 667–676. doi:10.1016/j.lpm.2018.06.001. |  |
| Cannabis lighting | 416 | Brown, A. S. (2011). The environment and susceptibility to schizophrenia. *Prog. Neurobiol.* 93, 23–58. doi:10.1016/j.pneurobio.2010.09.003. |  |
| Cannabis lighting | 417 | Broyd, S. J., van Hell, H. H., Beale, C., Yuecel, M., and Solowij, N. (2016). Acute and Chronic Effects of Cannabinoids on Human Cognition-A Systematic Review. *Biol. Psychiatry* 79, 557–567. doi:10.1016/j.biopsych.2015.12.002. |  |
| Cannabis lighting | 418 | Brunt, T. M., and Bossong, M. G. The neuropharmacology of cannabinoid receptor ligands in central signaling pathways. *Eur. J. Neurosci.* doi:10.1111/ejn.14982. |  |
| Cannabis lighting | 419 | Bucholz, K. K., McCutcheon, V. V, Agrawal, A., Dick, D. M., Hesselbrock, V. M., Kramer, J. R., et al. (2017). Comparison of Parent, Peer, Psychiatric, and Cannabis Use Influences Across Stages of Offspring Alcohol Involvement: Evidence from the COGA Prospective Study. *Alcohol. Exp. Res.* 41, 359–368. doi:10.1111/acer.13293. |  |
| Cannabis lighting | 420 | Buckner, J. D., Zvolensky, M. J., Businelle, M. S., and Gallagher, M. W. (2018). Direct and indirect effects of false safety behaviors on cannabis use and related problems. *Am. J. Addict.* 27, 29–34. doi:10.1111/ajad.12659. |  |
| Cannabis lighting | 421 | Buja, A., Lion, C., Scioni, M., Vian, P., Genetti, B., Vittadello, F., et al. (2017). SOGS-RA gambling scores and substance use in adolescents. *J. Behav. Addict.* 6, 425–433. doi:10.1556/2006.6.2017.043. |  |
| Cannabis lighting | 422 | Buja, A., Mortali, C., Mastrobattista, L., Minutillo, A., Pichini, S., Genetti, B., et al. (2019). Pathways connecting socioeconomic variables, substance abuse and gambling behaviour: a cross-sectional study on a sample of Italian high-school students. *BMJ Open* 9. doi:10.1136/bmjopen-2019-031737. |  |
| Cannabis lighting | 423 | Bujarski, S. J., Galang, J. N., Short, N. A., Trafton, J. A., Gifford, E. V, Kimerling, R., et al. (2016). Cannabis Use Disorder Treatment Barriers and Facilitators Among Veterans With PTSD. *Psychol. Addict. Behav.* 30, 73–81. doi:10.1037/adb0000131. |  |
| Cannabis lighting | 424 | Cabeen, R. P., Allman, J. M., and Toga, A. W. (2020). THC Exposure is Reflected in the Microstructure of the Cerebral Cortex and Amygdala of Young Adults. *Cereb. CORTEX* 30, 4949–4963. doi:10.1093/cercor/bhaa087. |  |
| Cannabis lighting | 425 | Cakici Es, A., Cakici, M., Iskender, C., and Kizilgul, Z. (2020). Psychoactive substance use profile and risk factors in TRNC risky regions: TRNC four regions sample. *ANADOLU Psikiyatr. DERGISI-ANATOLIAN J. PSYCHIATRY* 21, 165–172. doi:10.5455/apd.58339. |  |
| Cannabis lighting | 426 | Caldeira, K. M., Arria, A. M., Allen, H. K., Bugbee, B. A., Vincent, K. B., and O’Grady, K. E. (2017). Continuity of drunk and drugged driving behaviors four years post-college. *Drug Alcohol Depend.* 180, 332–339. doi:10.1016/j.drugalcdep.2017.08.032. |  |
| Cannabis lighting | 427 | Callaghan, R. C., Allebeck, P., and Sidorchuk, A. (2013). Marijuana use and risk of lung cancer: a 40-year cohort study. *CANCER CAUSES Control* 24, 1811–1820. doi:10.1007/s10552-013-0259-0. |  |
| Cannabis lighting | 428 | Campbell, L. G., Naraine, S. G. U., and Dusfresne, J. (2019). Phenotypic plasticity influences the success of clonal propagation in industrial pharmaceutical Cannabis sativa. *PLoS One* 14. doi:10.1371/journal.pone.0213434. |  |
| Cannabis lighting | 429 | Carrieri, V., Madio, L., and Principe, F. (2019). Light cannabis and organized crime: Evidence from (unintended) liberalization in Italy. *Eur. Econ. Rev.* 113, 63–76. doi:10.1016/j.euroecorev.2019.01.003. |  |
| Cannabis lighting | 430 | Carrieri, V., Madio, L., and Principe, F. (2020). Do-It-Yourself medicine? The impact of light cannabis liberalization on prescription drugs. *J. Health Econ.* 74. doi:10.1016/j.jhealeco.2020.102371. |  |
| Cannabis lighting | 431 | Castel, P., Simon, P., Barbier, M., Sunyach, C., Tassistro, V., Manzoni, O., et al. (2020). Focus on the endocannabinoid system and the reprotoxicity of marijuana in female users. *Gynecol. Obstet. Fertil. \& Senol.* 48, 384–392. doi:10.1016/j.gofs.2020.01.024. |  |
| Cannabis lighting | 432 | Caulkins, J. P., Davenport, S., Doanvo, A., Furlong, K., Siddique, A., Turner, M., et al. (2019). Triangulating web & general population surveys: Do results match legal cannabis market sales? *Int. J. DRUG POLICY* 73, 293–300. doi:10.1016/j.drugpo.2019.06.010. |  |
| Cannabis lighting | 433 | Caulkins, J. P., Pardo, B., and Kilmer, B. (2020). Intensity of cannabis use: Findings from three online surveys. *Int. J. DRUG POLICY* 79. doi:10.1016/j.drugpo.2020.102740. |  |
| Cannabis lighting | 434 | Cecho, R., Baska, T., Svihrova, V., and Hudeckova, H. (2017). LEGISLATIVE NORMS TO CONTROL CANNABIS USE IN THE LIGHT OF ITS PREVALENCE IN THE CZECH REPUBLIC, POLAND, SLOVAKIA, AND HUNGARY. *Cent. Eur. J. Public Health* 25, 261–265. doi:10.21101/cejph.a5019. |  |
| Cannabis lighting | 435 | Celerier, E., Ahdepil, T., Wikander, H., Berrendero, F., Nyberg, F., and Maldonado, R. (2006). Influence of the anabolic-androgenic steroid nandrolone on cannabinoid dependence. *Neuropharmacology* 50, 788–806. doi:10.1016/j.neuropharm.2005.11.017. |  |
| Cannabis lighting | 436 | Chagas, M. H. N., Crippa, J. A. S., Zuardi, A. W., Hallak, J. E. C., Machado-de-Sousa, J. P., Hirotsu, C., et al. (2013). Effects of acute systemic administration of cannabidiol on sleep-wake cycle in rats. *J. Psychopharmacol.* 27, 312–316. doi:10.1177/0269881112474524. |  |
| Cannabis lighting | 437 | Chalmers, J., and Ritter, A. (2011). The business cycle and drug use in Australia: Evidence from repeated cross-sections of individual level data. *Int. J. DRUG POLICY* 22, 341–352. doi:10.1016/j.drugpo.2011.03.006. |  |
| Cannabis lighting | 438 | Chan, N. W., Burkhardt, J., and Flyr, M. (2020). THE EFFECTS OF RECREATIONAL MARIJUANA LEGALIZATION AND DISPENSING ON OPIOID MORTALITY. *Econ. Inq.* 58, 589–606. doi:10.1111/ecin.12819. |  |
| Cannabis lighting | 439 | Chandra, S., Lata, H., Mehmedic, Z., Khan, I. A., and ElSohly, M. A. (2015). Light dependence of photosynthesis and water vapor exchange characteristics in different high Delta(9)-THC yielding varieties of Cannabis sativa L. *J. Appl. Res. Med. Aromat. PLANTS* 2, 39–47. doi:10.1016/j.jarmap.2015.03.002. |  |
| Cannabis lighting | 440 | Charron, C. B., and Leung, J. M. (2019). The Safety and Efficacy of Marijuana in Persons Living with HIV. *AIDS Rev.* 21, 84–92. doi:10.24875/AIDSRev.19000060. |  |
| Cannabis lighting | 441 | Chauhan, A., Verma, R., Kumari, S., Sharma, A., Shandilya, P., Li, X., et al. (2020). Photocatalytic dye degradation and antimicrobial activities of Pure and Ag-doped ZnO using Cannabis sativa leaf extract. *Sci. Rep.* 10. doi:10.1038/s41598-020-64419-0. |  |
| Cannabis lighting | 442 | Chen, T., Yao, S., Merlin, M., Mai, H., Qiu, Z., Hu, Y., et al. (2014). Identification of Cannabis Fiber from the Astana Cemeteries, Xinjiang, China, with Reference to Its Unique Decorative Utilization. *Econ. Bot.* 68, 59–66. doi:10.1007/s12231-014-9261-z. |  |
| Cannabis lighting | 443 | Cheung, J. T. W., Mann, R. E., Ialomiteanu, A., Stoduto, G., Chan, V., Ala-Leppilampi, K., et al. (2010). Anxiety and Mood Disorders and Cannabis Use. *Am. J. Drug Alcohol Abuse* 36, 118–122. doi:10.3109/00952991003713784. |  |
| Cannabis lighting | 444 | Choi, J.-S., Lee, N. Y., Oh, S.-E., Son, K.-C., and Kim, E.-S. (2011). Developmental Ultrastructure of Glandular Trichomes of Rosmarinus officinalis: Secretory Cavity and Secretory Vesicle Formation. *J. PLANT Biol.* 54, 135–142. doi:10.1007/s12374-011-9157-8. |  |
| Cannabis lighting | 445 | Chousidis, I., Chatzimitakos, T., Leonardos, D., Filiou, M. D., Stalikas, C. D., and Leonardos, I. D. (2020). Cannabinol in the spotlight: Toxicometabolomic study and behavioral analysis of zebrafish embryos exposed to the unknown cannabinoid. *Chemosphere* 252. doi:10.1016/j.chemosphere.2020.126417. |  |
| Cannabis lighting | 446 | Citti, C., Pacchetti, B., Vandelli, M. A., Forni, F., and Cannazza, G. (2018). Analysis of cannabinoids in commercial hemp seed oil and decarboxylation kinetics studies of cannabidiolic acid (CBDA). *J. Pharm. Biomed. Anal.* 149, 532–540. doi:10.1016/j.jpba.2017.11.044. |  |
| Cannabis lighting | 447 | Clark, D. B., Kirisci, L., and Tarter, R. E. (1998). Adolescent versus adult onset and the development of substance use disorders in males. *Drug Alcohol Depend.* 49, 115–121. doi:10.1016/S0376-8716(97)00154-3. |  |
| Cannabis lighting | 448 | Colizzi, M., and Bhattacharyya, S. (2018). “Neurocognitive effects of cannabis: Lessons learned from human experimental studies,” in *PSYCHEDELIC NEUROSCIENCE* Progress in Brain Research., ed. Calvey, T, 179–216. doi:10.1016/bs.pbr.2018.08.010. |  |
| Cannabis lighting | 449 | Colizzi, M., Weltens, N., McGuire, P., Van Oudenhove, L., and Bhattacharyya, S. (2019). Descriptive Psychopathology of the Acute Effects of Intravenous Delta-9-Tetrahydrocannabinol Administration in Humans. *BRAIN Sci.* 9. doi:10.3390/brainsci9040093. |  |
| Cannabis lighting | 450 | Comelli, F., Giagnoni, G., Bettoni, I., Colleoni, M., and Costa, B. (2008). Antihyperalgesic effect of a Cannabis sativa extract in a rat model of neuropathic pain: Mechanisms involved. *Phyther. Res.* 22, 1017–1024. doi:10.1002/ptr.2401. |  |
| Cannabis lighting | 451 | Conroy, D. A., and Arnedt, J. T. (2014). Sleep and Substance Use Disorders: An Update. *Curr. Psychiatry Rep.* 16. doi:10.1007/s11920-014-0487-3. |  |
| Cannabis lighting | 452 | Cope, Z. A., Halberstadt, A. L., van Enkhuizen, J., Flynn, A. D., Breier, M., Swerdlow, N. R., et al. (2016). Premature responses in the five-choice serial reaction time task reflect rodents’ temporal strategies: evidence from no-light and pharmacological challenges. *Psychopharmacology (Berl).* 233, 3513–3525. doi:10.1007/s00213-016-4389-4. |  |
| Cannabis lighting | 453 | Copur, M., Turkcan, A., and Erdogmus, M. (2005). Substance abuse, conduct disorder and crime: Assessment in a juvenile detention house in Istanbul, Turkey. *PSYCHIATRY Clin. Neurosci.* 59, 151–154. doi:10.1111/j.1440-1819.2005.01350.x. |  |
| Cannabis lighting | 454 | Correa, J. B., Myers, M. G., Tully, L. K., and Doran, N. (2020). Co-occurring Use of Cannabis and Tobacco and the Presence of Acute Respiratory Symptoms among Young Adult Light and Intermittent Smokers. *Subst. USE \& MISUSE* 55, 2129–2137. doi:10.1080/10826084.2020.1793366. |  |
| Cannabis lighting | 455 | Cosentino, S. L., Riggi, E., Testa, G., Scordia, D., and Copani, V. (2013). Evaluation of European developed fibre hemp genotypes (Cannabis sativa L.) in semi-arid Mediterranean environment. *Ind. Crops Prod.* 50, 312–324. doi:10.1016/j.indcrop.2013.07.059. |  |
| Cannabis lighting | 456 | Costa, M. A. (2016). The endocannabinoid system: A novel player in human placentation. *Reprod. Toxicol.* 61, 58–67. doi:10.1016/j.reprotox.2016.03.002. |  |
| Cannabis lighting | 457 | CRAWLEY, J. N., CORWIN, R. L., ROBINSON, J. K., FELDER, C. C., DEVANE, W. A., and AXELROD, J. (1993). ANANDAMIDE, AN ENDOGENOUS LIGAND OF THE CANNABINOID RECEPTOR, INDUCES HYPOMOTILITY AND HYPOTHERMIA IN-VIVO IN RODENTS. *Pharmacol. Biochem. Behav.* 46, 967–972. doi:10.1016/0091-3057(93)90230-Q. |  |
| Cannabis lighting | 458 | Crocker, C. E., and Tibbo, P. G. (2018). The interaction of gender and cannabis in early phase psychosis. *Schizophr. Res.* 194, 18–25. doi:10.1016/j.schres.2017.04.046. |  |
| Cannabis lighting | 459 | Cronce, J. M., Bittinger, J. N., Di Lodovico, C. M., and Liu, J. (2017). Independent Versus Co-occurring Substance Use in Relation to Gambling Outcomes in Older Adolescents and Young Adults. *J. Adolesc. Heal.* 60, 528–533. doi:10.1016/j.jadohealth.2016.10.021. |  |
| Cannabis lighting | 460 | Crook, C. L., Savin, M. J., Byrd, D., Summers, A. C., Guzman, V. A., Morris, E. P., et al. The neurocognitive effects of a past cannabis use disorder in a diverse sample of people living with HIV. *AIDS CARE-PSYCHOLOGICAL SOCIO-MEDICAL Asp. AIDS/HIV*. doi:10.1080/09540121.2020.1822504. |  |
| Cannabis lighting | 461 | D’Agostino, A., Gismondi, A., Di Marco, G., Lo Castro, M., Olevano, R., Cinti, T., et al. (2019). Lifestyle of a Roman Imperial community: ethnobotanical evidence from dental calculus of the Ager Curensis inhabitants. *J. Ethnobiol. Ethnomed.* 15. doi:10.1186/s13002-019-0334-z. |  |
| Cannabis lighting | 462 | D’Souza, D. C., Pittman, B., Perry, E., and Simen, A. (2009). Preliminary evidence of cannabinoid effects on brain-derived neurotrophic factor (BDNF) levels in humans. *Psychopharmacology (Berl).* 202, 569–578. doi:10.1007/s00213-008-1333-2. |  |
| Cannabis lighting | 463 | Dark, P. (2005). Mid- to late-Holocene vegetational and land-use change in the Hadrian’s Wall region: a radiocarbon-dated pollen sequence from Crag Lough, Northumberland, England. *J. Archaeol. Sci.* 32, 601–618. doi:10.1016/j.jas.2004.11.009. |  |
| Cannabis lighting | 464 | Daughtry, C. S. T., and Walthall, C. L. (1998). Spectral discrimination of Cannabis sativa L. leaves and canopies. *Remote Sens. Environ.* 64, 192–201. doi:10.1016/S0034-4257(98)00002-9. |  |
| Cannabis lighting | 465 | Dautzenberg, B. (2019). Tobacco and children: Birth of an addiction. *Bull. L Acad. Natl. Med.* 203, 549–556. doi:10.1016/j.banm.2019.06.009. |  |
| Cannabis lighting | 466 | Davis, J. P., Christie, N. C., Pakdaman, S., Hummer, J. F., DeLeon, J., Clapp, J. D., et al. (2020). Multifaceted impulsivity as a moderator of social anxiety and cannabis use during pregaming. *J. Anxiety Disord.* 76. doi:10.1016/j.janxdis.2020.102320. |  |
| Cannabis lighting | 467 | Day, N. L., Goldschmidt, L., and Thomas, C. A. (2006). Prenatal marijuana exposure contributes to the prediction of marijuana use at age 14. *ADDICTION* 101, 1313–1322. doi:10.1111/j.1360-0443.2006.01523.x. |  |
| Cannabis lighting | 468 | de Bruijn, P. B., Jeppsson, K.-H., Sandin, K., and Nilsson, C. (2009). Mechanical properties of lime-hemp concrete containing shives and fibres. *Biosyst. Eng.* 103, 474–479. doi:10.1016/j.biosystemseng.2009.02.005. |  |
| Cannabis lighting | 469 | de Meijer, E. P. M., Hammond, K. M., and Micheler, M. (2009). The inheritance of chemical phenotype in Cannabis sativa L. (III): variation in cannabichromene proportion. *EUPHYTICA* 165, 293–311. doi:10.1007/s10681-008-9787-1. |  |
| Cannabis lighting | 470 | Decorte, T. (2010). The case for small-scale domestic cannabis cultivation. *Int. J. Drug Policy* 21, 271–275. doi:10.1016/j.drugpo.2010.01.009. |  |
| Cannabis lighting | 471 | Degenhardt, L., Cheng, H., and Anthony, J. C. (2007). Assessing cannabis dependence in community surveys: methodological issues. *Int. J. Methods Psychiatr. Res.* 16, 43–51. doi:10.1002/mpr.206. |  |
| Cannabis lighting | 472 | Dei Cas, M., Casagni, E., Saccardo, A., Arnoldi, S., Young, C., Scotti, S., et al. (2020). The Italian panorama of cannabis light preparation: Determination of cannabinoids by LC-UV. *FORENSIC Sci. Int.* 307. doi:10.1016/j.forsciint.2019.110113. |  |
| Cannabis lighting | 473 | Deiana, S. (2013). Medical use of cannabis. Cannabidiol: A new light for schizophrenia? *DRUG Test. Anal.* 5, 46–51. doi:10.1002/dta.1425. |  |
| Cannabis lighting | 474 | Delourme, J., Delattre, C., Godard, P., Steenhouwer, F., and Just, N. (2009). Respiratory consequences of inhalation of adulterated cannabis. *Rev. Mal. Respir.* 26, 552–556. doi:10.1016/S0761-8425(09)74675-3. |  |
| Cannabis lighting | 475 | Demant, J., and Ravn, S. (2010). Identifying drug risk perceptions in Danish youths: Ranking exercises in focus groups. *DRUGS-EDUCATION Prev. POLICY* 17, 528–543. doi:10.3109/09687630903286818. |  |
| Cannabis lighting | 476 | Denton, T. M., Schmidt, S., Critchley, C., and Stewart, G. R. (2001). Natural abundance of stable carbon and nitrogen isotopes in Cannabis sativa reflects growth conditions. *Aust. J. Plant Physiol.* 28, 1005–1012. |  |
| Cannabis lighting | 477 | Deogan, C., Zarabi, N., Stenstrom, N., Hogberg, P., Skarstrand, E., Manrique-Garcia, E., et al. (2015). Cost-Effectiveness of School-Based Prevention of Cannabis Use. *Appl. Health Econ. Health Policy* 13, 525–542. doi:10.1007/s40258-015-0175-4. |  |
| Cannabis lighting | 478 | Dhawan, K., Kumar, S., and Sharma, A. (2002). Reversal of cannabinoids (Delta(9)-THC) by the benzoflavone moiety from methanol extract of Passiflora incarnata Linneaus in mice: a possible therapy for cannabinoid addiction. *J. Pharm. Pharmacol.* 54, 875–881. doi:10.1211/0022357021779069. |  |
| Cannabis lighting | 479 | Di Bello, M. P., Bloise, E., Mazzetto, S. E., and Mele, G. (2017). Formulation and Chemical Stability in Aqueous Media of Cannabidiol Embedded in Cardanol-Based Nanovesicles. *ACS Sustain. Chem. Eng.* 5, 8870–8875. doi:10.1021/acssuschemeng.7b01658. |  |
| Cannabis lighting | 480 | Di Forti, M., Dempster, E., Quattrone, D., Hannon, E., Burrage, J., Mansell, G., et al. (2019a). CAN DNA METHYLATION PROFILING SHED LIGHT ON THE BIOLOGY OF CANNABIS ASSOCIATED PSYCHOSIS? PRELIMINARY DATA ON THE EWAS CANNABIS USE SIGNATURE IN THE EUGEI CASE-CONTROL STUDY. *Eur. Neuropsychopharmacol.* 29, 1221. doi:10.1016/j.euroneuro.2018.08.283. |  |
| Cannabis lighting | 481 | Di Forti, M., Dempster, E., Quattrone, D., Tripoli, G., Kandaswamy, R., Morgan, C., et al. (2019b). DNA METHYLATION PROFILING MIGHT SHED LIGHT ON THE BIOLOGY OF CANNABIS ASSOCIATED PSYCHOSIS. *Schizophr. Bull.* 45, S122. doi:10.1093/schbul/sbz022.082. |  |
| Cannabis lighting | 482 | Di Marzo, V., and Silvestri, C. (2019). Lifestyle and Metabolic Syndrome: Contribution of the Endocannabinoidome. *Nutrients* 11. doi:10.3390/nu11081956. |  |
| Cannabis lighting | 483 | Di Pietro, M. C., Doering-Silveira, E. B., Oliveira, M. P. T., Rosa-Oliveira, L. Q., and Da Silveira, D. X. (2007). Factors associated with the use of solvents and cannabis by medical students. *Addict. Behav.* 32, 1740–1744. doi:10.1016/j.addbeh.2006.12.004. |  |
| Cannabis lighting | 484 | Dimitrijevic, I., Aksic, M., Aleksic, D., Dimitrijevic, N., Andelic, S., Stijak, L., et al. (2013). EFFECTS OF TETRA HYDRO CANNABINOL TO THE DENDRITC TREE AND SYNAPSES OF THE ACCUMBENS NUCLEUS OF WISTAR RATS. *ACTA Vet.* 63, 37–44. doi:10.2298/AVB1301037D. |  |
| Cannabis lighting | 485 | Dinu, A. R., Rogobete, A. F., Bratu, T., Popovici, S. E., Bedreag, O. H., Papurica, M., et al. (2020). Cannabis Sativa Revisited-Crosstalk between microRNA Expression, Inflammation, Oxidative Stress, and Endocannabinoid Response System in Critically Ill Patients with Sepsis. *CELLS* 9. doi:10.3390/cells9020307. |  |
| Cannabis lighting | 486 | Doerr, A., Espinoza, A., and Acevedo, J. (2014). The experience of time in habitual teenage marijuana smokers. *ACTAS Esp. Psiquiatr.* 42, 49–56. |  |
| Cannabis lighting | 487 | Donoghue, K., and Doody, G. A. (2012). Effect of Illegal Substance Use on Cognitive Function in Individuals With a Psychotic Disorder, A Review and Meta-Analysis. *Neuropsychology* 26, 785–801. doi:10.1037/a0029685. |  |
| Cannabis lighting | 488 | Donohue, B., Hill, H. H., Azrin, N. H., Cross, C., and Strada, M. J. (2007). Psychometric support for contemporaneous and retrospective youth and parent reports of adolescent marijuana use frequency in an adolescent outpatient treatment population. *Addict. Behav.* 32, 1787–1797. doi:10.1016/j.addbeh.2006.12.005. |  |
| Cannabis lighting | 489 | Donohue, B., Plant, C. P., Chow, G., Schubert, K., Bradshaw, K., Cappellano, J. U., et al. (2019). Contribution of Illicit/Non-Prescribed Marijuana and Hard-Drug Use to Child-Abuse and Neglect Potential while Considering Social Desirability. *Br. J. Soc. Work* 49, 77–95. doi:10.1093/bjsw/bcy027. |  |
| Cannabis lighting | 490 | Dorn, N. (2004). UK policing of drug traffickers and users: Policy implementation in the contexts of national law, European traditions, international drug conventions, and security after 2001. *J. Drug Issues* 34, 533–550. doi:10.1177/002204260403400304. |  |
| Cannabis lighting | 491 | Doss, M. K., Weafer, J., Gallo, D. A., and de Wit, H. (2018). Delta(9)-Tetrahydrocannabinol at Retrieval Drives False Recollection of Neutral and Emotional Memories. *Biol. Psychiatry* 84, 743–750. doi:10.1016/j.biopsych.2018.04.020. |  |
| Cannabis lighting | 492 | Dughiero, G., Schifano, F., and Forza, G. (2001). Personality dimensions and psychopathological profiles of Ecstasy users. *Hum. Psychopharmacol. Exp.* 16, 635–639. doi:10.1002/hup.346. |  |
| Cannabis lighting | 493 | Eaves, J., Eaves, S., Morphy, C., and Murray, C. (2020). The relationship between light intensity, cannabis yields, and profitability. *Agron. J.* 112, 1466–1470. doi:10.1002/agj2.20008. |  |
| Cannabis lighting | 494 | Eggan, S. M., Melchitzky, D. S., Sesack, S. R., Fish, K. N., and Lewis, D. A. (2010). RELATIONSHIP OF CANNABINOID CB1 RECEPTOR AND CHOLECYSTOKININ IMMUNOREACTIVITY IN MONKEY DORSOLATERAL PREFRONTAL CORTEX. *Neuroscience* 169, 1651–1661. doi:10.1016/j.neuroscience.2010.06.011. |  |
| Cannabis lighting | 495 | Egginton, R., and Parker, H. (2002). From one-off triers to regular users: Measuring the regularity of drug taking in a cohort of English adolescents (1996-1999). *Addict. Res. Theory* 10, 97–114. doi:10.1080/16066350290001597. |  |
| Cannabis lighting | 496 | Eide, A. H., and Acuda, S. W. (1997). Cultural orientation and use of cannabis and inhalants among secondary school children in Zimbabwe. *Soc. Sci. Med.* 45, 1241–1249. doi:10.1016/S0277-9536(97)00052-X. |  |
| Cannabis lighting | 497 | El Marroun, H., Bolhuis, K., Franken, I. H. A., Jaddoe, V. W. V, Hillegers, M. H., Lahey, B. B., et al. (2019). Preconception and prenatal cannabis use and the risk of behavioural and emotional problems in the offspring; a multi-informant prospective longitudinal study. *Int. J. Epidemiol.* 48, 287–296. doi:10.1093/ije/dyy186. |  |
| Cannabis lighting | 498 | Ellickson, P. L., Martino, S. C., and Collins, R. L. (2004). Marijuana use from adolescence to young adulthood: Multiple developmental trajectories and their associated outcomes. *Heal. Psychol.* 23, 299–307. doi:10.1037/0278-6133.23.3.299. |  |
| Cannabis lighting | 499 | Elmes, M. W., Kaczocha, M., Berger, W. T., Leung, K., Ralph, B. P., Wang, L., et al. (2015). Fatty Acid-binding Proteins (FABPs) Are Intracellular Carriers for Delta(9)-Tetrahydrocannabinol (THC) and Cannabidiol (CBD). *J. Biol. Chem.* 290, 8711–8721. doi:10.1074/jbc.M114.618447. |  |
| Cannabis lighting | 500 | EMRICH, H. M., WEBER, M. M., WENDL, A., ZIHL, J., VONMEYER, L., and HANISCH, W. (1991). REDUCED BINOCULAR DEPTH INVERSION AS AN INDICATOR OF CANNABIS-INDUCED CENSORSHIP IMPAIRMENT. *Pharmacol. Biochem. Behav.* 40, 689–690. doi:10.1016/0091-3057(91)90383-D. |  |
| Cannabis lighting | 501 | Enns, A., and Orpana, H. (2020). Autonomy, competence and relatedness and cannabis and alcohol use among youth in Canada: a cross-sectional analysis. *Heal. Promot. CHRONIC Dis. Prev. CANADA-RESEARCH POLICY Pract.* 40, 201–210. doi:10.24095/hpcdp.40.5/6.09. |  |
| Cannabis lighting | 502 | Esch, P., Bocquet, V., Pull, C., Couffignal, S., Graas, M., Lair, M.-L., et al. (2011). Psychosocial risk and protective factors of secondary school dropout in Luxembourg: the protocol of an exploratory case-control study. *BMC Public Health* 11. doi:10.1186/1471-2458-11-555. |  |
| Cannabis lighting | 503 | Fallu, J.-S., Briere, F. N., and Janosz, M. (2014). Latent classes of substance use in adolescent cannabis users: predictors and subsequent substance-related harm. *Front. PSYCHIATRY* 5. doi:10.3389/fpsyt.2014.00009. |  |
| Cannabis lighting | 504 | Fernandez-Tendero, E., Day, A., Legros, S., Habrant, A., Hawkins, S., and Chabbert, B. (2017). Changes in hemp secondary fiber production related to technical fiber variability revealed by light microscopy and attenuated total reflectance Fourier transform infrared spectroscopy. *PLoS One* 12. doi:10.1371/journal.pone.0179794. |  |
| Cannabis lighting | 505 | Fiani, B., Sarhadi, K. J., Soula, M., Zafar, A., and Quadri, S. A. (2020). Current application of cannabidiol (CBD) in the management and treatment of neurological disorders. *Neurol. Sci.* 41, 3085–3098. doi:10.1007/s10072-020-04514-2. |  |
| Cannabis lighting | 506 | Fisk, J. E., and Montgomery, C. (2009). Evidence for selective executive function deficits in ecstasy/polydrug users. *J. Psychopharmacol.* 23, 40–50. doi:10.1177/0269881108089815. |  |
| Cannabis lighting | 507 | Fitzgerald, P. B., Williams, S., and Daskalakis, Z. J. (2009). A Transcranial Magnetic Stimulation Study of the Effects of Cannabis Use on Motor Cortical Inhibition and Excitability. *NEUROPSYCHOPHARMACOLOGY* 34, 2368–2375. doi:10.1038/npp.2009.71. |  |
| Cannabis lighting | 508 | Floether, L., Peitek, K., Bucher, M., and Benndorf, R. A. (2019). Medical Cannabis-Related Relapse in a Patient with a History of Alcohol Abuse. *Dtsch. MEDIZINISCHE WOCHENSCHRIFT* 144, 1135–1137. doi:10.1055/a-0805-1973. |  |
| Cannabis lighting | 509 | Franz, F., Angerer, V., Jechle, H., Pegoro, M., Ertl, H., Weinfurtner, G., et al. (2017). Immunoassay screening in urine for synthetic cannabinoids - an evaluation of the diagnostic efficiency. *Clin. Chem. Lab. Med.* 55, 1375–1384. doi:10.1515/cclm-2016-0831. |  |
| Cannabis lighting | 510 | French, L., Gray, C., Leonard, G., Perron, M., Pike, G. B., Richer, L., et al. (2015). Early Cannabis Use, Polygenic Risk Score for Schizophrenia, and Brain Maturation in Adolescence. *JAMA PSYCHIATRY* 72, 1002–1011. doi:10.1001/jamapsychiatry.2015.1131. |  |
| Cannabis lighting | 511 | Fried, P. A., Watkinson, B., and Gray, R. (2005). Neurocognitive consequences of marihuana - a comparison with pre-drug performance. *Neurotoxicol. Teratol.* 27, 231–239. doi:10.1016/j.ntt.2004.11.003. |  |
| Cannabis lighting | 512 | Fried, P., Watkinson, B., James, D., and Gray, F. (2002). Current and former marijuana use: preliminary findings of a longitudinal study of effects on IQ in Young adults. *Can. Med. Assoc. J.* 166, 887–891. |  |
| Cannabis lighting | 513 | Fucci, N. (2003). Growing cannabis with naphthalene in Rome. *FORENSIC Sci. Int.* 138, 91–93. doi:10.1016/j.forsciint.2003.08.007. |  |
| Cannabis lighting | 514 | Gallardo, E., Barroso, M., and Queiroz, J. A. (2009). LC-MS: a powerful tool in workplace drug testing. *DRUG Test. Anal.* 1, 109–115. doi:10.1002/dta.26. |  |
| Cannabis lighting | 515 | Gallate, J. E., Saharov, T., Mallet, P. E., and McGregor, I. S. (1999). Increased motivation for beer in rats following administration of a cannabinoid CB1 receptor agonist. *Eur. J. Pharmacol.* 370, 233–240. doi:10.1016/S0014-2999(99)00170-3. |  |
| Cannabis lighting | 516 | Giasson-Gariepy, K., Potvin, S., Ghabrash, M., Bruneau, J., and Jutras-Aswad, D. (2017). Cannabis and cue-induced craving in cocaine-dependent individuals: A pilot study. *Addict. Behav.* 73, 4–8. doi:10.1016/j.addbeh.2017.03.025. |  |
| Cannabis lighting | 517 | Gilbert, D. G., Rabinovich, N. E., and McDaniel, J. T. (2020). Nicotine patch for cannabis withdrawal symptom relief: a randomized controlled trial. *Psychopharmacology (Berl).* 237, 1507–1519. doi:10.1007/s00213-020-05476-1. |  |
| Cannabis lighting | 518 | Giupponi, L., Leoni, V., Carrer, M., Ceciliani, G., Sala, S., Panseri, S., et al. (2020). Overview on Italian hemp production chain, related productive and commercial activities and legislative framework. *Ital. J. Agron.* 15, 194–205. doi:10.4081/ija.2020.1552. |  |
| Cannabis lighting | 519 | Gloss, D. (2015). An Overview of Products and Bias in Research. *NEUROTHERAPEUTICS* 12, 731–734. doi:10.1007/s13311-015-0370-x. |  |
| Cannabis lighting | 520 | Goldschmidt, L., Richardson, G. A., Cornelius, M. D., and Day, N. L. (2004). Prenatal marijuana and alcohol exposure and academic achievement at age 10. *Neurotoxicol. Teratol.* 26, 521–532. doi:10.1016/j.ntt.2004.04.003. |  |
| Cannabis lighting | 521 | Gomes-Medeiros, D., de Faria, P. H., de Sousa Campos, G. W., and Tofoli, L. F. (2019). Drug policy and Collective Health: necessary dialogues. *Cad. Saude Publica* 35. doi:10.1590/0102-311X00242618. |  |
| Cannabis lighting | 522 | Goyal, H., Singla, U., Gupta, U., and May, E. (2017). Role of cannabis in digestive disorders. *Eur. J. Gastroenterol. Hepatol.* 29, 135–143. doi:10.1097/MEG.0000000000000779. |  |
| Cannabis lighting | 523 | Grafstrom, K., Andersson, K., Pettersson, N., Dalgaard, J., and Dunne, S. J. (2019). Effects of long term storage on secondary metabolite profiles of cannabis resin. *FORENSIC Sci. Int.* 301, 331–340. doi:10.1016/j.forsciint.2019.05.035. |  |
| Cannabis lighting | 524 | Grard, A., Kunst, A., Kuipers, M., Richter, M., Rimpela, A., Federico, B., et al. (2018). Same-Sex Friendship, School Gender Composition, and Substance Use: A Social Network Study of 50 European Schools. *Subst. Use Misuse* 53, 998–1007. doi:10.1080/10826084.2017.1392976. |  |
| Cannabis lighting | 525 | Gray, K. M., Sonne, S. C., McClure, E. A., Ghitza, U. E., Matthews, A. G., McRae-Clark, A. L., et al. (2017). A randomized placebo-controlled trial of N-acetylcysteine for cannabis use disorder in adults. *Drug Alcohol Depend.* 177, 249–257. doi:10.1016/j.drugalcdep.2017.04.020. |  |
| Cannabis lighting | 526 | Greenwood, C. J., Youssef, G. J., Betts, K. S., Letcher, P., Mcintosh, J., Spry, E., et al. (2019). A comparison of longitudinal modelling approaches: Alcohol and cannabis use from adolescence to young adulthood. *Drug Alcohol Depend.* 201, 58–64. doi:10.1016/j.drugalcdep.2019.05.001. |  |
| Cannabis lighting | 527 | Gressler, L. E., Baltz, A. P., Costantino, R. C., Slejko, J. F., and Onukwugha, E. (2021). Exploring the Use of State Medical Cannabis Legislation as a Proxy for Medical Cannabis Use Among Patients Receiving Chemotherapy. *Curr. Treat. Options Oncol.* 22. doi:10.1007/s11864-020-00803-2. |  |
| Cannabis lighting | 528 | Grossman, M., Bowie, C. R., Lepage, M., Malla, A. K., Joober, R., and Iyer, S. N. (2017). Smoking status and its relationship to demographic and clinical characteristics in first episode psychosis. *J. Psychiatr. Res.* 85, 83–90. doi:10.1016/j.jpsychires.2016.10.022. |  |
| Cannabis lighting | 529 | Guerriero, G., Behr, M., Backes, A., Faleri, C., Hausman, J.-F., Lutts, S., et al. (2017a). Bast fibre formation: insights from Next-Generation Sequencing. in *3RD INTERNATIONAL CONFERENCE ON NATURAL FIBERS: ADVANCED MATERIALS FOR A GREENER WORLD, ICNF 2017* Procedia Engineering., ed. Fangueiro, R, 229–235. doi:10.1016/j.proeng.2017.07.033. |  |
| Cannabis lighting | 530 | Guerriero, G., Behr, M., Hausman, J.-F., and Legay, S. (2017b). Textile Hemp vs. Salinity: Insights from a Targeted Gene Expression Analysis. *Genes (Basel).* 8. doi:10.3390/genes8100242. |  |
| Cannabis lighting | 531 | Gukasyan, N., and Strain, E. C. (2020). Relationship between cannabis use frequency and major depressive disorder in adolescents: Findings from the National Survey on Drug Use and Health 2012-2017. *Drug Alcohol Depend.* 208. doi:10.1016/j.drugalcdep.2020.107867. |  |
| Cannabis lighting | 532 | Gunderson, E. W., Haughey, H. M., Ait-Daoud, N., Joshi, A. S., and Hart, C. L. (2012). ``Spice{’’} and ``K2{’’} Herbal Highs: A Case Series and Systematic Review of the Clinical Effects and Biopsychosocial Implications of Synthetic Cannabinoid Use in Humans. *Am. J. Addict.* 21, 320–326. doi:10.1111/j.1521-0391.2012.00240.x. |  |
| Cannabis lighting | 533 | Haardoerfer, R., Berg, C. J., Lewis, M., Payne, J., Pillai, D., McDonald, B., et al. (2016). Polytobacco, marijuana, and alcohol use patterns in college students: A latent class analysis. *Addict. Behav.* 59, 58–64. doi:10.1016/j.addbeh.2016.03.034. |  |
| Cannabis lighting | 534 | Hadland, S. E., Knight, J. R., and Harris, S. K. (2015). Medical Marijuana: Review of the Science and Implications for Developmental-Behavioral Pediatric Practice. *J. Dev. Behav. Pediatr.* 36, 115–123. doi:10.1097/DBP.0000000000000129. |  |
| Cannabis lighting | 535 | Haines-Saah, R. J., Johnson, J. L., Repta, R., Ostry, A., Young, M. L., Shoveller, J., et al. (2014). The privileged normalization of marijuana use - an analysis of Canadian newspaper reporting, 1997-2007. *Crit. Public Health* 24, 47–61. doi:10.1080/09581596.2013.771812. |  |
| Cannabis lighting | 536 | Haines, N., Vassileva, J., and Ahn, W.-Y. (2018). The Outcome-Representation Learning Model: A Novel Reinforcement Learning Model of the Iowa Gambling Task. *Cogn. Sci.* 42, 2534–2561. doi:10.1111/cogs.12688. |  |
| Cannabis lighting | 537 | Hall, J., Bhattarai, S. P., and Midmore, D. J. (2012). Review of Flowering Control in Industrial Hemp. *J. Nat. FIBERS* 9, 23–36. doi:10.1080/15440478.2012.651848. |  |
| Cannabis lighting | 538 | Hall, J., Bhattarai, S. P., and Midmore, D. J. (2014). The Effects of Photoperiod on Phenological Development and Yields of Industrial Hemp. *J. Nat. FIBERS* 11, 87–106. doi:10.1080/15440478.2013.846840. |  |
| Cannabis lighting | 539 | Hall, W. D., and Lynskey, M. (2005). Is cannabis a gateway drug? Testing hypotheses about the relationship between cannabis use and the use of other illicit drugs. *Drug Alcohol Rev.* 24, 39–48. doi:10.1080/09595230500126698. |  |
| Cannabis lighting | 540 | Hall, W., Degenhardt, L., and Teesson, M. (2004). Cannabis use and psychotic disorders: an update. *Drug Alcohol Rev.* 23, 433–443. doi:10.1080/09595230412331324554. |  |
| Cannabis lighting | 541 | Haller, S., Curtis, L., Badan, M., Bessero, S., Albom, M., Chantraine, F., et al. (2013). Combined Grey Matter VBM and White Matter TBSS Analysis in Young First Episode Psychosis Patients With and Without Cannabis Consumption. *BRAIN Topogr.* 26, 641–647. doi:10.1007/s10548-013-0288-8. |  |
| Cannabis lighting | 542 | Hambrecht, M., and Hafner, H. (1996). Does substance abuse result in schizophrenia? *NERVENARZT* 67, 36–45. |  |
| Cannabis lighting | 543 | Hammond, C. J. (2019). ``A LIGHT IN THE DARKNESS?{’’} RELATIONSHIPS BETWEEN CANNABIS USE AND NEGATIVE EMOTIONALITY AT THE DISORDER, SYMPTOM, TREATMENT RESPONSE, AND CIRCUIT LEVEL. *J. Am. Acad. CHILD Adolesc. PSYCHIATRY* 58, S91–S92. doi:10.1016/j.jaac.2019.07.508. |  |
| Cannabis lighting | 544 | Han, X., He, Y., Bi, G.-H., Zhang, H.-Y., Song, R., Liu, Q.-R., et al. (2017). CB1 Receptor Activation on VgluT2-Expressing Glutamatergic Neurons Underlies Delta(9)-Tetrahydrocannabinol (Delta(9)-THC)-Induced Aversive Effects in Mice. *Sci. Rep.* 7. doi:10.1038/s41598-017-12399-z. |  |
| Cannabis lighting | 545 | Hanninen, T., Thygesen, A., Mehmood, S., Madsen, B., and Hughes, M. (2012). Mechanical processing of bast fibres: The occurrence of damage and its effect on fibre structure. *Ind. Crops Prod.* 39, 7–11. doi:10.1016/j.indcrop.2012.01.025. |  |
| Cannabis lighting | 546 | Hanus, L. O., Martin Meyer, S., Munoz, E., Taglialatela-Scafati, O., and Appendino, G. (2016). Phytocannabinoids: a unified critical inventory. *Nat. Prod. Rep.* 33, 1357–1392. doi:10.1039/c6np00074f. |  |
| Cannabis lighting | 547 | Happyana, N., and Kayser, O. (2020). Metabolic Changes in the Trichomes of Cannabis sativa var. bedrobinol Analyzed by H-1-NMR-Based Metabolomics. *Indones. J. Chem.* 20, 1246–1254. doi:10.22146/ijc.48765. |  |
| Cannabis lighting | 548 | Hartman, R. L., Richman, J. E., Hayes, C. E., and Huestis, M. A. (2016). Drug Recognition Expert (DRE) examination characteristics of cannabis impairment. *Accid. Anal. Prev.* 92, 219–229. doi:10.1016/j.aap.2016.04.012. |  |
| Cannabis lighting | 549 | Hasan, A. R., Poddar, C. K., Prasad, B. K., and Singh, M. N. (2018). COMPARATIVE STUDY OF PULMONARY FUNCTION TESTS AMONG SMOKERS AND NON-SMOKERS IN A TERTIARY CARE HOSPITAL IN KOSHI REGION (NORTHERN BIHAR), INDIA. *J. Evol. Med. Dent. Sci.* 7, 1341–1346. doi:10.14260/jemds/2018/305. |  |
| Cannabis lighting | 550 | Hasan, S. S., Shaikh, A., Ochani, R. K., Ashrafi, M. M., Ansari, Z. N., Abbas, S. H., et al. Perception and practices regarding cannabis consumption in Karachi, Pakistan: A cross-sectional study. *J. Ethn. Subst. Abuse*. doi:10.1080/15332640.2019.1667287. |  |
| Cannabis lighting | 551 | Hathaway, A. D., Comeau, N. C., and Erickson, P. G. (2011). Cannabis normalization and stigma: Contemporary practices of moral regulation. *Criminol. Crim. JUSTICE* 11, 451–469. doi:10.1177/1748895811415345. |  |
| Cannabis lighting | 552 | Hathaway, A., Mostaghim, A., Kolar, K., Erickson, P. G., and Osborne, G. (2016). A nuanced view of normalisation: Attitudes of cannabis non-users in a study of undergraduate students at three Canadian universities. *Drugs Educ. Prev. Policy* 23, 238–246. doi:10.3109/09687637.2015.1112362. |  |
| Cannabis lighting | 553 | Hawley, D., Graham, T., Stasiak, M., and Dixon, M. (2018). Improving Cannabis Bud Quality and Yield with Subcanopy Lighting. *HORTSCIENCE* 53, 1593–1599. doi:10.21273/HORTSCI13173-18. |  |
| Cannabis lighting | 554 | Heffernan, T. M., Ling, J., Parrott, A. C., Buchanan, T., Scholey, A. B., and Rodgers, J. (2005). Self-rated everyday and prospective memory abilities of cigarette smokers and non-smokers: a web-based study. *Drug Alcohol Depend.* 78, 235–241. doi:10.1016/j.drugalcdep.2004.11.008. |  |
| Cannabis lighting | 555 | Helyes, Z., Kemeny, A., Cseko, K., Szoke, E., Elekes, K., Mester, M., et al. (2017). Marijuana smoke induces severe pulmonary hyperresponsiveness, inflammation, and emphysema in a predictive mouse model not via CB1 receptor activation. *Am. J. Physiol. Cell. Mol. Physiol.* 313, L267–L277. doi:10.1152/ajplung.00354.2016. |  |
| Cannabis lighting | 556 | HEMPHILL, J. K., MAHLBERG, P. G., TURNER, J. C., and HUGHES, W. (1976). EFFECTS OF WAVELENGTHS OF LIGHT ON CANNABINOID COMPOSITION IN CANNABIS. *PLANT Physiol.* 57, 106. |  |
| Cannabis lighting | 557 | Herbig, C., and Sirocko, F. (2013). Palaeobotanical evidence for agricultural activities in the Eifel region during the Holocene: plant macro-remain and pollen analyses from sediments of three maar lakes in the Quaternary Westeifel Volcanic Field (Germany, Rheinland-Pfalz). *Veg. Hist. Archaeobot.* 22, 447–462. doi:10.1007/s00334-012-0387-6. |  |
| Cannabis lighting | 558 | Hernandez-Estrada, A., Reza, M., and Hughes, M. (2020). The Structure of Dislocations in Hemp (Cannabis sativa L.) Fibres and Implications for Mechanical Behaviour. *BIORESOURCES* 15, 2579–2595. doi:10.15376/biores.15.2.2579-2595. |  |
| Cannabis lighting | 559 | Hesselink, J. M. K. (2013). Evolution in pharmacologic thinking around the natural analgesic palmitoylethanolamide: from nonspecific resistance to PPAR-alpha agonist and effective nutraceutical. *J. Pain Res.* 6, 625–634. doi:10.2147/JPR.S48653. |  |
| Cannabis lighting | 560 | Hickman, M., Vickerman, P., Macleod, J., Kirkbride, J., and Jones, P. B. (2007). Cannabis and schizophrenia: model projections of the impact of the rise in cannabis use on historical and future trends in schizophrenia in England and Wales. *ADDICTION* 102, 597–606. doi:10.1111/j.1360-0443.2006.01710.x. |  |
| Cannabis lighting | 561 | Hickman, M., Vickerman, P., Macleod, J., Lewis, G., Zammit, S., Kirkbride, J., et al. (2009). If cannabis caused schizophrenia-how many cannabis users may need to be prevented in order to prevent one case of schizophrenia? England and Wales calculations. *ADDICTION* 104, 1856–1861. doi:10.1111/j.1360-0443.2009.02736.x. |  |
| Cannabis lighting | 562 | Highet, G. (2003). Cannabis and smoking research: interviewing young people in self-selected friendship pairs. *Health Educ. Res.* 18, 108–118. doi:10.1093/her/18.1.108. |  |
| Cannabis lighting | 563 | Ho, M.-C., Chang, C. F., Li, R.-H., and Tang, T.-C. (2013). Attentional Biases for Betel Nut Cues in Heavy and Light Chewers. *Psychol. Addict. Behav.* 27, 1044–1049. doi:10.1037/a0030562. |  |
| Cannabis lighting | 564 | Holland, B. J., Francis, P. S., Li, B., Tsuzuki, T., Adcock, J. L., Barnett, N. W., et al. (2012). Chemiluminescence detection of cannabinoids and related compounds with acidic potassium permanganate. *DRUG Test. Anal.* 4, 675–679. doi:10.1002/dta.1328. |  |
| Cannabis lighting | 565 | HOLLISTER, L. E., and KANTER, S. L. (1980). LABORATORY VERIFICATION OF HEAVY AND LIGHT USERS OF CANNABIS. *Drug Alcohol Depend.* 5, 151–152. doi:10.1016/0376-8716(80)90192-1. |  |
| Cannabis lighting | 566 | Hughes, M., Mott, L., Hague, J., and Hill, C. A. S. (1999). The toughness of vegetable fiber-reinforced unsaturated polyester composites. in *FIFTH INTERNATIONAL CONFERENCE ON WOODFIBER-PLASTIC COMPOSITES*, 175–184. |  |
| Cannabis lighting | 567 | Impey, D., El-Marj, N., Parks, A., Choueiry, J., Fisher, D., and Knott, V. J. (2015). Mismatch negativity in tobacco-naive cannabis users and its alteration with acute nicotine administration. *Pharmacol. Biochem. Behav.* 136, 73–81. doi:10.1016/j.pbb.2015.07.002. |  |
| Cannabis lighting | 568 | Iseppi, R., Brighenti, V., Licata, M., Lambertini, A., Sabia, C., Messi, P., et al. (2019). Chemical Characterization and Evaluation of the Antibacterial Activity of Essential Oils from Fibre-Type Cannabis sativa L. (Hemp). *MOLECULES* 24. doi:10.3390/molecules24122302. |  |
| Cannabis lighting | 569 | Izgelov, D., Freidman, M., and Hoffman, A. (2020). Investigation of cannabidiol gastro retentive tablets based on regional absorption of cannabinoids in rats. *Eur. J. Pharm. Biopharm.* 152, 229–235. doi:10.1016/j.ejpb.2020.05.010. |  |
| Cannabis lighting | 570 | James, P. D., Comiskey, C., and Smyth, B. P. (2019). ``Debt on Me Head{’’} A Qualitative Study of the Experience of Teenage Cannabis Users in Treatment. *J. Addict. Nurs.* 30, 211–218. doi:10.1097/JAN.0000000000000301. |  |
| Cannabis lighting | 571 | Jewell, R. S., Thomas, J. D., and Dodds, R. A. (2011). Attempted ignition of petrol vapour by lit cigarettes and lit cannabis resin joints. *Sci. JUSTICE* 51, 72–76. doi:10.1016/j.scijus.2010.10.002. |  |
| Cannabis lighting | 572 | Jones, B. T., Bruce, G., Livingstone, S., and Reed, E. (2006). Alcohol-related attentional bias in problem drinkers with the flicker change blindness paradigm. *Psychol. Addict. Behav.* 20, 171–177. doi:10.1037/0893-164X.20.2.171. |  |
| Cannabis lighting | 573 | Joya, X., Papaseit, E., Civit, E., Pellegrini, M., Vall, O., Garcia-Algar, O., et al. (2009). Unsuspected Exposure to Cocaine in Preschool Children From a Mediterranean City Detected by Hair Analysis. *Ther. Drug Monit.* 31, 391–395. doi:10.1097/FTD.0b013e31819c3f2b. |  |
| Cannabis lighting | 574 | Justinova, Z., Munzar, P., Panlilio, L. V, Yasar, S., Redhi, G. H., Tanda, G., et al. (2008). Blockade of THC-Seeking Behavior and relapse in monkeys by the cannabinoid CB(1)-Receptor antagonist rimonabant. *NEUROPSYCHOPHARMACOLOGY* 33, 2870–2877. doi:10.1038/npp.2008.21. |  |
| Cannabis lighting | 575 | Kalliomaki, J., Philipp, A., Baxendale, J., Annas, P., Karlsten, R., and Segerdahl, M. (2012). Lack of effect of central nervous system-active doses of nabilone on capsaicin-induced pain and hyperalgesia. *Clin. Exp. Pharmacol. Physiol.* 39, 336–342. doi:10.1111/j.1440-1681.2012.05674.x. |  |
| Cannabis lighting | 576 | Karis, K., Eskla, K.-L., Kaare, M., Taht, K., Tuusov, J., Visnapuu, T., et al. (2018). Altered Expression Profile of IgLON Family of Neural Cell Adhesion Molecules in the Dorsolateral Prefrontal Cortex of Schizophrenic Patients. *Front. Mol. Neurosci.* 11. doi:10.3389/fnmol.2018.00008. |  |
| Cannabis lighting | 577 | Karoly, H. C., Schacht, J. P., Meredith, L. R., Jacobus, J., Tapert, S. F., Gray, K. M., et al. (2019). Investigating a novel fMRI cannabis cue reactivity task in youth. *Addict. Behav.* 89, 20–28. doi:10.1016/j.addbeh.2018.09.015. |  |
| Cannabis lighting | 578 | Kaskie, B., Bobitt, J., Herrera, J., Bhagianadh, D., Segal-Gidan, F., Brummel-Smith, K., et al. (2021). Cannabis Use among Persons with Dementia and Their Caregivers: Lighting up an Emerging Issue for Clinical Gerontologists. *Clin. Gerontol.* 44, 42–52. doi:10.1080/07317115.2020.1852465. |  |
| Cannabis lighting | 579 | Kaur, M., Sharma, A., Kaur, R., Katnoria, J. K., and Nagpal, A. K. (2016). Palynological studies of some roadside plants under exposure to traffic stress. *Aerobiologia (Bologna).* 32, 245–254. doi:10.1007/s10453-015-9394-2. |  |
| Cannabis lighting | 580 | Kavallieratos, N. G., Boukouvala, M. C., Ntalli, N., Skourti, A., Karagianni, E. S., Nika, E. P., et al. (2020). Effectiveness of eight essential oils against two key stored-product beetles, Prostephanus truncatus (Horn) and Trogoderma granarium Everts. *FOOD Chem. Toxicol.* 139. doi:10.1016/j.fct.2020.111255. |  |
| Cannabis lighting | 581 | Keith, D. R., Hart, C. L., McNeil, M. P., Silver, R., and Goodwin, R. D. (2015). Frequent marijuana use, binge drinking and mental health problems among undergraduates. *Am. J. Addict.* 24, 499–506. doi:10.1111/ajad.12201. |  |
| Cannabis lighting | 582 | Kendler, K. S., Loenn, S. L., Sundquist, J., and Sundquist, K. (2015). Smoking and Schizophrenia in Population Cohorts of Swedish Women and Men: A Prospective Co-Relative Control Study. *Am. J. Psychiatry* 172, 1092–1100. doi:10.1176/appi.ajp.2015.15010126. |  |
| Cannabis lighting | 583 | Khajuria, M., Rahul, V. P., and Vyas, D. (2020). Photochemical efficiency is negatively correlated with the Delta(9) - tetrahydrocannabinol content in Cannabis sativa L. *PLANT Physiol. Biochem.* 151, 589–600. doi:10.1016/j.plaphy.2020.04.003. |  |
| Cannabis lighting | 584 | Khan, M. M. R., Chen, Y., Belsham, T., Lague, C., Landry, H., Peng, Q., et al. (2011). Fineness and tensile properties of hemp (Cannabis sativa L.) fibres. *Biosyst. Eng.* 108, 9–17. doi:10.1016/j.biosystemseng.2010.10.004. |  |
| Cannabis lighting | 585 | Khlat, M., Van Cleemput, O., Bricard, D., and Legleye, S. (2020). Use of tobacco, alcohol and cannabis in late adolescence: roles of family living arrangement and socioeconomic group. *BMC Public Health* 20. doi:10.1186/s12889-020-09476-w. |  |
| Cannabis lighting | 586 | Khokhar, J. Y., Dwiel, L. L., Henricks, A. M., Doucette, W. T., and Green, A. I. (2018). The link between schizophrenia and substance use disorder: A unifying hypothesis. *Schizophr. Res.* 194, 78–85. doi:10.1016/j.schres.2017.04.016. |  |
| Cannabis lighting | 587 | Kim, D., Kim, W., Kwak, M.-S., Chung, G. E., Yim, J. Y., and Ahmed, A. (2017). Inverse association of marijuana use with nonalcoholic fatty liver disease among adults in the United States. *PLoS One* 12. doi:10.1371/journal.pone.0186702. |  |
| Cannabis lighting | 588 | Kim, E. S., and Mahlberg, P. G. (2000). Early development of the secretory cavity of peltate glands in Humulus lupulus L. (Cannabaceae). *Mol. Cells* 10, 487–492. doi:10.1007/s10059-000-0487-5. |  |
| Cannabis lighting | 589 | Kim, E. S., and Mahlberg, P. G. (2003). Secretory vesicle formation in the secretory cavity of glandular trichomes of Cannabis sativa L. (Cannabaceae). *Mol. Cells* 15, 387–395. |  |
| Cannabis lighting | 590 | Kleis, J., Germerott, T., Halter, S., Heroux, V., Roehrich, J., Schwarz, C. S., et al. (2020). The synthetic cannabinoid 5F-MDMB-PICA: A case series. *FORENSIC Sci. Int.* 314. doi:10.1016/j.forsciint.2020.110410. |  |
| Cannabis lighting | 591 | Kmietowicz, Z. (2010). Countries should consider legalising cannabis in light of futility of bans. *Br. Med. J.* 341. doi:10.1136/bmj.c5471. |  |
| Cannabis lighting | 592 | Koola, M. M., McMahon, R. P., Wehring, H. J., Liu, F., Mackowick, K. M., Warren, K. R., et al. (2012). Alcohol and cannabis use and mortality in people with schizophrenia and related psychotic disorders. *J. Psychiatr. Res.* 46, 987–993. doi:10.1016/j.jpsychires.2012.04.019. |  |
| Cannabis lighting | 593 | Kopruszinski, C. M., Navratilova, E., Vagnerova, B., Swiokla, J., Patwardhan, A., Dodick, D., et al. (2020). Cannabinoids induce latent sensitization in a preclinical model of medication overuse headache. *CEPHALALGIA* 40, 68–78. doi:10.1177/0333102419865252. |  |
| Cannabis lighting | 594 | Korn, L., Haynie, D. L., Luk, J. W., and Simons-Morton, B. G. (2018). Prospective associations between cannabis use and negative and positive health and social measures among emerging adults. *Int. J. DRUG POLICY* 58, 55–63. doi:10.1016/j.drugpo.2018.05.003. |  |
| Cannabis lighting | 595 | Korponay, C., Kosson, D. S., Decety, J., Kiehl, K. A., and Koenigs, M. (2017). Brain Volume Correlates With Duration of Abstinence From Substance Abuse in a Region-Specific and Substance-Specific Manner. *Biol. PSYCHIATRY-COGNITIVE Neurosci. NEUROIMAGING* 2, 626–635. doi:10.1016/j.bpsc.2017.03.011. |  |
| Cannabis lighting | 596 | Kraszkiewicz, A., Kachel, M., Parafiniuk, S., Zajac, G., Niedziolka, I., and Sprawka, M. (2019). Assessment of the Possibility of Using Hemp Biomass (Cannabis Sativa L.) for Energy Purposes: A Case Study. *Appl. Sci.* 9. doi:10.3390/app9204437. |  |
| Cannabis lighting | 597 | Krauss, M. J., Rajbhandari, B., Sowles, S. J., Spitznagel, E. L., and Cavazos-Rehg, P. (2017). A latent class analysis of poly-marijuana use among young adults. *Addict. Behav.* 75, 159–165. doi:10.1016/j.addbeh.2017.07.021. |  |
| Cannabis lighting | 598 | Kristjansson, A. L., Sigfusdottir, I. D., and Allegrante, J. P. (2013). Adolescent substance use and peer use: a multilevel analysis of cross-sectional population data. *Subst. Abus. Treat. Prev. POLICY* 8. doi:10.1186/1747-597X-8-27. |  |
| Cannabis lighting | 599 | Kurzthaler, I., Hummer, M., Miller, C., Sperner-Unterweger, B., Gunther, V., Wechdorn, H., et al. (1999). Effect of cannabis use on cognitive functions and driving ability. *J. Clin. Psychiatry* 60, 395–399. doi:10.4088/JCP.v60n0609. |  |
| Cannabis lighting | 600 | Kuypers, K. P. C., Perna, E. B. de S. F., Dolder, P. C., Toennes, S. W., Theunissen, E. L., Mason, N. L., et al. (2018). Drug liking and wanting, not impulsive action or reflection is increased by 4-fluoroamphetamine. *Psychopharmacology (Berl).* 235, 2349–2356. doi:10.1007/s00213-018-4931-7. |  |
| Cannabis lighting | 601 | Labra, V. C., Santibanez, C. A., Gajardo-Gomez, R., Diaz, E. F., Gomez I, G., and Orellana, J. A. (2018). The Neuroglial Dialog Between Cannabinoids and Hemichannels. *Front. Mol. Neurosci.* 11. doi:10.3389/fnmol.2018.00079. |  |
| Cannabis lighting | 602 | Lafaye, G., Desterke, C., Marulaz, L., and Benyamina, A. (2019). Cannabidiol affects circadian clock core complex and its regulation in microglia cells. *Addict. Biol.* 24, 921–934. doi:10.1111/adb.12660. |  |
| Cannabis lighting | 603 | Lake, S., Kerr, T., Capler, R., Shoveller, J., Montaner, J., and Milloy, M.-J. (2017). High-intensity cannabis use and HIV clinical outcomes among HIV-positive people who use illicit drugs in Vancouver, Canada. *Int. J. DRUG POLICY* 42, 63–70. doi:10.1016/j.drugpo.2017.02.009. |  |
| Cannabis lighting | 604 | Lalge, A., Cerny, P., Trojan, V., and Vyhnanek, T. (2017). THE EFFECTS OF RED, BLUE AND WHITE LIGHT ON THE GROWTH AND DEVELOPMENT OF CANNABIS SATIVA L. in *PROCEEDINGS OF 24TH INTERNATIONAL PHD STUDENTS CONFERENCE (MENDELNET 2017)*, ed. Cerkal, R and Belcredi, NB and Prokesova, L and Vacek, P, 646–651. |  |
| Cannabis lighting | 605 | Landi, S., Berni, R., Capasso, G., Hausman, J.-F., Guerriero, G., and Esposito, S. (2019). Impact of Nitrogen Nutrition on Cannabis sativa: An Update on the Current Knowledge and Future Prospects. *Int. J. Mol. Sci.* 20. doi:10.3390/ijms20225803. |  |
| Cannabis lighting | 606 | Lascaux, M., Ionescu, S., and Phan, O. (2016). Effectiveness of formalised therapy for adolescents with cannabis dependence: A randomised trial. *DRUGS-EDUCATION Prev. POLICY* 23, 404–409. doi:10.3109/09687637.2016.1153603. |  |
| Cannabis lighting | 607 | Lata, H., Chandra, S., Khan, I., and ElSohly, M. A. (2009). Thidiazuron-induced high-frequency direct shoot organogenesis of Cannabis sativa L. *Vitr. Cell. Dev. Biol.* 45, 12–19. doi:10.1007/s11627-008-9167-5. |  |
| Cannabis lighting | 608 | Laudien, R., Schultze, R., and Wieser, J. (2010). Fast detection of narcotics by single photon ionization mass spectrometry and laser ion mobility spectrometry. in *OPTICS AND PHOTONICS FOR COUNTERTERRORISM AND CRIME FIGHTING VI AND OPTICAL MATERIALS IN DEFENCE SYSTEMS TECHNOLOGY VII* Proceedings of SPIE-The International Society for Optical Engineering., ed. Lewis, C and Burgess, D and Zamboni, R and Kajzar, F and Heckman, EM doi:10.1117/12.864697. |  |
| Cannabis lighting | 609 | Leal, W. E., and Jackson, D. B. (2019). The role of energy drink consumption in the intention to initiate marijuana use among adolescents. *Addict. Behav.* 93, 240–245. doi:10.1016/j.addbeh.2019.02.008. |  |
| Cannabis lighting | 610 | Legleye, S., Kraus, L., Piontek, D., Phan, O., and Jouanne, C. (2012). Validation of the Cannabis Abuse Screening Test in a Sample of Cannabis Inpatients. *Eur. Addict. Res.* 18, 193–200. doi:10.1159/000336553. |  |
| Cannabis lighting | 611 | Lenne, M. G., Fry, C. L. M., Dietze, P., and Rumbold, G. (2001). Attitudes and experiences of people who use cannabis and drive: implications for drugs and driving legislation in Victoria, Australia. *DRUGS-EDUCATION Prev. POLICY* 8, 307–313. |  |
| Cannabis lighting | 612 | Leos-Toro, C., Fong, G. T., Meyer, S. B., and Hammond, D. (2020). Cannabis labelling and consumer understanding of THC levels and serving sizes. *Drug Alcohol Depend.* 208. doi:10.1016/j.drugalcdep.2020.107843. |  |
| Cannabis lighting | 613 | Lerner, A. G., Goodman, C., Bor, O., and Ran, S. L. (2014). Synthetic Cannabis Substances (SPS) Use and Hallucinogen Persisting Perception Disorder (HPPD): Two Case Reports. *Isr. J. Psychiatry Relat. Sci.* 51, 277–280. |  |
| Cannabis lighting | 614 | Lev-Ran, S., Roerecke, M., Le Foll, B., George, T. P., McKenzie, K., and Rehm, J. (2014). The association between cannabis use and depression: a systematic review and meta-analysis of longitudinal studies. *Psychol. Med.* 44, 797–810. doi:10.1017/S0033291713001438. |  |
| Cannabis lighting | 615 | Levy-Cooperman, N., Harrison, S. J., Chen, N. L., and Chakraborty, B. (2012). Differences in cannabinoid-induced changes in cognitive performance in light stimulant and cannabis users versus heavy cannabis users. *Eur. Neuropsychopharmacol.* 22, S403–S404. doi:10.1016/S0924-977X(12)70630-2. |  |
| Cannabis lighting | 616 | Liang, Y.-C., Huang, C.-C., and Hsu, K.-S. (2004). Therapeutic Potential of Cannabinoids in Trigeminal Neuralgia. *CNS \& Neurol. Disord. TARGETS* 3, 507–514. doi:10.2174/1568007043336833. |  |
| Cannabis lighting | 617 | Linden-Carmichael, A. N., and Allen, H. K. Profiles of Alcohol and Marijuana Use Among Simultaneous Alcohol and Marijuana Users: Individual Differences in Demographics and Substance Use. *J. Drug Issues*. doi:10.1177/0022042620979617. |  |
| Cannabis lighting | 618 | LINDGREN, J. E., OHLSSON, A., AGURELL, S., HOLLISTER, L., and GILLESPIE, H. (1981). CLINICAL EFFECTS AND PLASMA-LEVELS OF DELTA-9-TETRAHYDROCANNABINOL (DELTA-9-THC) IN HEAVY AND LIGHT USERS OF CANNABIS. *Psychopharmacology (Berl).* 74, 208–212. doi:10.1007/BF00427095. |  |
| Cannabis lighting | 619 | Linger, P., Ostwald, A., and Haensler, J. (2005). Cannabis sativa L. growing on heavy metal contaminated soil: growth, cadmium uptake and photosynthesis. *Biol. Plant.* 49, 567–576. doi:10.1007/s10535-005-0051-4. |  |
| Cannabis lighting | 620 | Lippiello, P., Balestrini, S., Leo, A., Coppola, A., Citraro, R., Elia, M., et al. (2016). From Cannabis to Cannabidiol to Treat Epilepsy, Where Are We? *Curr. Pharm. Des.* 22, 6426–6433. doi:10.2174/1381612822666160831122722. |  |
| Cannabis lighting | 621 | Lisson, S. N., Mendham, N. J., and Carberry, P. S. (2000). Development of a hemp (Cannabis sativa L.) simulation model 3. The effect of plant density on leaf appearance, expansion and senescence. *Aust. J. Exp. Agric.* 40, 419–423. doi:10.1071/EA99060. |  |
| Cannabis lighting | 622 | Liu, Y., van den Wildenberg, W. P. M., de Graaf, Y., Ames, S. L., Baldacchino, A., Bo, R., et al. (2019). Is (poly-) substance Use associated with impaired inhibitory control? A fmega-analysis controlling for confounders. *Neurosci. Biobehav. Rev.* 105, 288–304. doi:10.1016/j.neubiorev.2019.07.006. |  |
| Cannabis lighting | 623 | Livadariu, O., Raiciu, D., Maximilian, C., and Capitanu, E. (2019). Studies regarding treatments of LED-s emitted fight on sprouting hemp (Cannabis sativa L.). *Rom. Biotechnol. Lett.* 24, 485–490. doi:10.25083/rbl/24.3/485.490. |  |
| Cannabis lighting | 624 | Loberg, E.-M., Helle, S., Nygard, M., Berle, J. O., Kroken, R. A., and Johnsen, E. (2014). The cannabis pathway to non-affective psychosis may reflect less neurobiological vulnerability. *Front. PSYCHIATRY* 5. doi:10.3389/fpsyt.2014.00159. |  |
| Cannabis lighting | 625 | Long, L. E., Chesworth, R., Arnold, J. C., and Karl, T. (2010a). A follow-up study: acute behavioural effects of Delta(9)-THC in female heterozygous Neuregulin 1 transmembrane domain mutant mice. *Psychopharmacology (Berl).* 211, 277–289. doi:10.1007/s00213-010-1896-6. |  |
| Cannabis lighting | 626 | Long, L. E., Chesworth, R., Huang, X.-F., McGregor, I. S., Arnold, J. C., and Karl, T. (2010b). A behavioural comparison of acute and chronic Delta(9)-tetrahydrocannabinol and cannabidiol in C57BL/6JArc mice. *Int. J. Neuropsychopharmacol.* 13, 861–876. doi:10.1017/S1461145709990605. |  |
| Cannabis lighting | 627 | Lucas, P. (2009). Moral regulation and the presumption of guilt in Health Canada’s medical cannabis policy and practice. *Int. J. DRUG POLICY* 20, 296–303. doi:10.1016/j.drugpo.2008.09.007. |  |
| Cannabis lighting | 628 | Lupi, M., Martinotti, G., Santacroce, R., Cinosi, E., Carlucci, M., Marini, S., et al. (2017). Transcranial Direct Current Stimulation in Substance Use Disorders: A Systematic Review of Scientific Literature. *J. ECT* 33, 203–209. doi:10.1097/YCT.0000000000000401. |  |
| Cannabis lighting | 629 | Lyketsos, C. G., Garrett, E., Liang, K. Y., and Anthony, J. C. (1999). Cannabis use and cognitive decline in persons under 65 years of age. *Am. J. Epidemiol.* 149, 794–800. |  |
| Cannabis lighting | 630 | Lynch, M. Themes and tones of cannabis news reports and legalization outcomes. *MEDIA Cult. \& Soc.* doi:10.1177/0163443720960905. |  |
| Cannabis lighting | 631 | Lyvers, M. (2006). Recreational ecstasy use and the neurotoxic potential of MDMA: current status of the controversy and methodological issues. *Drug Alcohol Rev.* 25, 269–276. doi:10.1080/09595230600657758. |  |
| Cannabis lighting | 632 | Maccarrone, M., Bab, R., Biro, T., Cabral, G. A., Dey, S. K., Di Marzo, V., et al. (2015). Endocannabinoid signaling at the periphery: 50 years after THC. *TRENDS Pharmacol. Sci.* 36, 277–296. doi:10.1016/j.tips.2015.02.008. |  |
| Cannabis lighting | 633 | Macher, R., Guatney, L., and Earleywine, M. (2013). Effort During Neuropsychological Assessment in Heavy and Light Cannabis Users. *Arch. Clin. Neuropsychol.* 28, 551. |  |
| Cannabis lighting | 634 | Magnus, S., Hallgrimsdottir, H., Bates-Eamer, N., and Konrad, V. (2020). Overgrowing the Border? An Examination of Cascadian Culture and Cannabis Legalization. *J. Borderl. Stud.* 35, 505–526. doi:10.1080/08865655.2019.1619474. |  |
| Cannabis lighting | 635 | MAHLBERG, P. G., and HEMPHILL, J. K. (1983). EFFECT OF LIGHT QUALITY ON CANNABINOID CONTENT OF CANNABIS-SATIVA L (CANNABACEAE). *Bot. Gaz.* 144, 43–48. doi:10.1086/337342. |  |
| Cannabis lighting | 636 | Mai, H., Yang, Y., Jiang, H., Wang, B., and Wang, C. (2017). Investigating the materials and manufacture of Jinzi: The lining of Futou (Chinese traditional male headwear) from the Astana Cemeteries, Xinjiang, China. *J. Cult. Herit.* 27, 116–124. doi:10.1016/j.culher.2017.02.018. |  |
| Cannabis lighting | 637 | Maida, V., and Corban, J. (2017). Topical Medical Cannabis: A New Treatment for Wound Paind-Three Cases of Pyoderma Gangrenosum. *J. Pain Symptom Manage.* 54, 732–736. doi:10.1016/j.jpainsymman.2017.06.005. |  |
| Cannabis lighting | 638 | Mallat, A., Hezode, C., and Lotersztajn, S. (2008). Environmental factors as disease accelerators during chronic hepatitis C. *J. Hepatol.* 48, 657–665. doi:10.1016/j.jhep.2008.01.004. |  |
| Cannabis lighting | 639 | Mallett, K. A., Turrisi, R., Trager, B. M., Sell, N., and Linden-Carmichael, A. N. (2019). An Examination of Consequences Among College Student Drinkers on Occasions Involving Alcohol-Only, Marijuana-Only, or Combined Alcohol and Marijuana Use. *Psychol. Addict. Behav.* 33, 331–336. doi:10.1037/adb0000458. |  |
| Cannabis lighting | 640 | Mallinson, D. J., and Hannah, A. L. (2020). Policy and Political Learning: The Development of Medical Marijuana Policies in the States. *PUBLIUS-THE J. Fed.* 50, 344–369. doi:10.1093/publius/pjaa006. |  |
| Cannabis lighting | 641 | Manevski, K., Laerke, P. E., Jiao, X., Santhome, S., and Jorgensen, U. (2017). Biomass productivity and radiation utilisation of innovative cropping systems for biorefinery. *Agric. For. Meteorol.* 233, 250–264. doi:10.1016/j.agrformet.2016.11.245. |  |
| Cannabis lighting | 642 | Manly, J. T., Oshri, A., Lynch, M., Herzog, M., and Wortel, S. (2013). Child Neglect and the Development of Externalizing Behavior Problems: Associations With Maternal Drug Dependence and Neighborhood Crime. *Child Maltreat.* 18, 17–29. doi:10.1177/1077559512464119. |  |
| Cannabis lighting | 643 | Manning, K., Garey, L., Paulus, D. J., Buckner, J. D., Hogan, J. B. D., Schmidt, N. B., et al. (2019). Typology of cannabis use among adults: A latent class approach to risk and protective factors. *Addict. Behav.* 92, 6–13. doi:10.1016/j.addbeh.2018.12.008. |  |
| Cannabis lighting | 644 | Mantzoukas, S., Ntoukas, A., Lagogiannis, I., Kalyvas, N., Eliopoulos, P., and Poulas, K. (2020). Larvicidal Action of Cannabidiol Oil and Neem Oil against Three Stored Product Insect Pests: Effect on Survival Time and in Progeny. *BIOLOGY-BASEL* 9. doi:10.3390/biology9100321. |  |
| Cannabis lighting | 645 | Manza, P., Tomasi, D., and Volkow, N. D. (2018). Subcortical Local Functional Hyperconnectivity in Cannabis Dependence. *Biol. PSYCHIATRY-COGNITIVE Neurosci. NEUROIMAGING* 3, 285–293. doi:10.1016/j.bpsc.2017.11.004. |  |
| Cannabis lighting | 646 | Marangoni, C., Hernandez, M., and Faedda, G. L. (2016). The role of environmental exposures as risk factors for bipolar disorder: A systematic review of longitudinal studies. *J. Affect. Disord.* 193, 165–174. doi:10.1016/j.jad.2015.12.055. |  |
| Cannabis lighting | 647 | Marchei, E., Tittarelli, R., Pellegrini, M., Rotolo, M. C., Pacifici, R., and Pichini, S. (2020). Is ``light cannabis{’’} really light? Determination of cannabinoids content in commercial products. *Clin. Chem. Lab. Med.* 58, E175–E177. doi:10.1515/cclm-2020-0040. |  |
| Cannabis lighting | 648 | Marchini, M., Charvoz, C., Dujourdy, L., Baldovini, N., and Filippi, J.-J. (2014). Multidimensional analysis of cannabis volatile constituents: Identification of 5,5-dimethyl-1-vinylbicyclo{[}2.1.1]hexane as a volatile marker of hashish, the resin of Cannabis sativa L. *J. Chromatogr. A* 1370, 200–215. doi:10.1016/j.chroma.2014.10.045. |  |
| Cannabis lighting | 649 | Maremmani, A. G. I., Pani, P. P., Rovai, L., Bacciardi, S., and Maremmani, I. (2017). Toward the Identification of a Specific Psychopathology of Substance Use Disorders. *Front. PSYCHIATRY* 8. doi:10.3389/fpsyt.2017.00068. |  |
| Cannabis lighting | 650 | Mariani, J. J., Pavlicova, M., Choi, C. J., Basaraba, C., Carpenter, K. M., Mahony, A. L., et al. (2021). Quetiapine treatment for cannabis use disorder. *Drug Alcohol Depend.* 218. doi:10.1016/j.drugalcdep.2020.108366. |  |
| Cannabis lighting | 651 | Marini, E., Magi, G., Ferretti, G., Bacchetti, T., Giuliani, A., Pugnaloni, A., et al. (2018). Attenuation of Listeria monocytogenes Virulence by Cannabis sativa L. Essential Oil. *Front. Cell. Infect. Microbiol.* 8. doi:10.3389/fcimb.2018.00293. |  |
| Cannabis lighting | 652 | Mariotti, K. de C., Alexandre Marcelo, M. C., Ortiz, R. S., Borille, B. T., dos Reis, M., Fett, M. S., et al. (2016). Seized cannabis seeds cultivated in greenhouse: A chemical study by gas chromatography-mass spectrometry and chemometric analysis. *Sci. JUSTICE* 56, 35–41. doi:10.1016/j.scijus.2015.09.002. |  |
| Cannabis lighting | 653 | Marmet, S., Studer, J., Lemoine, M., Grazioli, V. S., Bertholet, N., and Gmel, G. (2019). Reconsidering the associations between self-reported alcohol use disorder and mental health problems in the light of co-occurring addictions in young Swiss men. *PLoS One* 14. doi:10.1371/journal.pone.0222806. |  |
| Cannabis lighting | 654 | Mashreghi, M., and Shayestehpour, S. (2011). Effect of various concentrations of Crocus sativus and Cannabis sativa extracts on luminescent biosensor Escherichia coli SM10 S1. *JUNDISHAPUR J. Microbiol.* 4, S35–S41. |  |
| Cannabis lighting | 655 | Mason, M. J., Zaharakis, N. M., Russell, M., and Childress, V. (2018). A pilot trial of text-delivered peer network counseling to treat young adults with cannabis use disorder. *J. Subst. Abuse Treat.* 89, 1–10. doi:10.1016/j.jsat.2018.03.002. |  |
| Cannabis lighting | 656 | Mason, W. A., Hanson, K., Fleming, C. B., Ringle, J. L., and Haggerty, K. P. (2015). Washington State Recreational Marijuana Legalization: Parent and Adolescent Perceptions, Knowledge, and Discussions in a Sample of Low-Income Families. *Subst. Use Misuse* 50, 541–545. doi:10.3109/10826084.2014.952447. |  |
| Cannabis lighting | 657 | Matyas, F., Urban, G. M., Watanabe, M., Mackie, K., Zimmer, A., Freund, T. F., et al. (2008). Identification of the sites of 2-arachidonoylglycerol synthesis and action imply retrograde endocannabinoid signaling at both GABAergic and glutamatergic synapses in the ventral tegmental area. *Neuropharmacology* 54, 95–107. doi:10.1016/j.neuropharm.2007.05.028. |  |
| Cannabis lighting | 658 | Mazian, B., Bergeret, A., Benezet, J.-C., and Malhautier, L. (2018). Influence of field retting duration on the biochemical, microstructural, thermal and mechanical properties of hemp fibres harvested at the beginning of flowering. *Ind. Crops Prod.* 116, 170–181. doi:10.1016/j.indcrop.2018.02.062. |  |
| Cannabis lighting | 659 | Mazina, J., Spiljova, A., Vaher, M., Kaljurand, M., and Kulp, M. (2015). A rapid capillary electrophoresis method with LED-induced native fluorescence detection for the analysis of cannabinoids in oral fluid. *Anal. METHODS* 7, 7741–7747. doi:10.1039/c5ay01595b. |  |
| Cannabis lighting | 660 | Mcdonnell, P. E. (2018). My experiences of psychosis and what caused it; my experiences with mental health services and other things that helped or hindered my journey. doi:10.1080/17522439.2018.1484507. |  |
| Cannabis lighting | 661 | McGarvey, P., Huang, J., McCoy, M., Orvis, J., Katsir, Y., Lotringer, N., et al. (2020). De novo assembly and annotation of transcriptomes from two cultivars of Cannabis sativa with diff erent cannabinoid profiles. *Gene* 762. doi:10.1016/j.gene.2020.145026. |  |
| Cannabis lighting | 662 | Mcgowan, M. K. (2020). Load Calculations for Cannabis Grow Facilities. *ASHRAE J.* 62, 82–87. |  |
| Cannabis lighting | 663 | McGrath, J. J., Saha, S., Al-Hamzawi, A., Andrade, L., Benjet, C., Bromet, E. J., et al. (2016). The Bidirectional Associations Between Psychotic Experiences and DSM-IV Mental Disorders. *Am. J. Psychiatry* 173, 997–1006. doi:10.1176/appi.ajp.2016.15101293. |  |
| Cannabis lighting | 664 | McMillan, B., Sherlock, K., and Conner, M. (2003). Expanding the traditional user versus non-user dichotomy amongst ecstasy users. *J. Community Appl. Soc. Psychol.* 13, 15–28. doi:10.1002/casp.704. |  |
| Cannabis lighting | 665 | Meacham, M. C., Vogel, E. A., and Thrul, J. (2020). Vaping-Related Mobile Apps Available in the Google Play Store After the Apple Ban: Content Review. *J. Med. INTERNET Res.* 22. doi:10.2196/20009. |  |
| Cannabis lighting | 666 | Mechoulam, R., and Parker, L. A. (2013). “The Endocannabinoid System and the Brain,” in *ANNUAL REVIEW OF PSYCHOLOGY, VOL 64* Annual Review of Psychology., ed. Fiske, ST, 21–47. doi:10.1146/annurev-psych-113011-143739. |  |
| Cannabis lighting | 667 | Mechoulam, R., Peters, M., Murillo-Rodrigiiez, E., and Hanus, L. O. (2007). Cannabidiol - Recent advances. *Chem. Biodivers.* 4, 1678–1692. doi:10.1002/cbdv.200790147. |  |
| Cannabis lighting | 668 | Meehan-Atrash, J., Korzun, T., and Ziegler, A. (2019a). Cannabis Inhalation and Voice Disorders A Systematic Review. *JAMA Otolaryngol. \& NECK Surg.* 145, 956–964. doi:10.1001/jamaoto.2019.1986. |  |
| Cannabis lighting | 669 | Meehan-Atrash, J., Luo, W., McWhirter, K. J., and Strongin, R. M. (2019b). Aerosol Gas-Phase Components from Cannabis E-Cigarettes and Dabbing: Mechanistic Insight and Quantitative Risk Analysis. *ACS OMEGA* 4, 16111–16120. doi:10.1021/acsomega.9b02301. |  |
| Cannabis lighting | 670 | Meijer, W. J. M., and Mathijssen, E. (1996). Analysis of crop performance in research on inulin, fibre and oilseed crops. *Ind. Crops Prod.* 5, 253–264. doi:10.1016/S0926-6690(97)82785-9. |  |
| Cannabis lighting | 671 | MEIJER, W. J. M., VANDERWERF, H. M. G., MATHIJSSEN, E., and VANDENBRINK, P. W. M. (1995). CONSTRAINTS TO DRY-MATTER PRODUCTION IN FIBER HEMP (CANNABIS-SATIVA L). *Eur. J. Agron.* 4, 109–117. doi:10.1016/S1161-0301(14)80022-1. |  |
| Cannabis lighting | 672 | Metrik, J., Aston, E. R., Kahler, C. W., Rohsenow, D. J., McGeary, J. E., Knopik, V. S., et al. (2016). Cue-elicited increases in incentive salience for marijuana: Craving, demand, and attentional bias. *Drug Alcohol Depend.* 167, 82–88. doi:10.1016/j.drugalcdep.2016.07.027. |  |
| Cannabis lighting | 673 | Meurer, M. M., Chakrala, K., Gowda, D., Burns, C., Kelly, R., and Schlabritz-Loutsevitch, N. (2018). A case of cannabinoid hyperemesis syndrome with Heliobacter pylori and preeclampsia during pregnancy. *Subst. Abus.* 39, 9–13. doi:10.1080/08897077.2017.1356790. |  |
| Cannabis lighting | 674 | Milani, R. M., Parrott, A. C., Turner, J. J. D., and Fox, H. C. (2004). Gender differences in self-reported anxiety, depression, and somatization among ecstasy/MDMA polydrug users, alcohol/tobacco users, and nondrug users. *Addict. Behav.* 29, 965–971. doi:10.1016/j.addbeh.2004.02.044. |  |
| Cannabis lighting | 675 | Minerbi, A., Haeuser, W., and Fitzcharles, M.-A. (2019). Medical Cannabis for Older Patients. *Drugs Aging* 36, 39–51. doi:10.1007/s40266-018-0616-5. |  |
| Cannabis lighting | 676 | Mirski, R., Dziurka, D., and Trocinski, A. (2018). Insulation Properties of Boards Made from Long Hemp (Cannabis sativa L.) Fibers. *BIORESOURCES* 13, 6591–6599. |  |
| Cannabis lighting | 677 | Mon, A., Durazzo, T. C., Abe, C., Gazdzinski, S., Pennington, D., Schmidt, T., et al. (2014). Structural brain differences in alcohol-dependent individuals with and without comorbid substance dependence. *Drug Alcohol Depend.* 144, 170–177. doi:10.1016/j.drugalcdep.2014.09.010. |  |
| Cannabis lighting | 678 | Mondino, A., Cavelli, M., Gonzalez, J., Santana, N., Castro-Zaballa, S., Mechoso, B., et al. (2019). Acute effect of vaporized Cannabis on sleep and electrocortical activity. *Pharmacol. Biochem. Behav.* 179, 113–123. doi:10.1016/j.pbb.2019.02.012. |  |
| Cannabis lighting | 679 | Montgomery, L., Robinson, C., Seaman, E. L., and Haeny, A. M. (2017). A Scoping Review and Meta-Analysis of Psychosocial and Pharmacological Treatments for Cannabis and Tobacco Use Among African Americans. *Psychol. Addict. Behav.* 31, 922–943. doi:10.1037/adb0000326. |  |
| Cannabis lighting | 680 | Moore, C., Vincent, M., Rana, S., Coulter, C., Agrawal, A., and Soares, J. (2006). Stability of Delta(9)-tetrahydrocannabinol (THC) in oral fluid using the Quantisal (TM) collection device. *FORENSIC Sci. Int.* 164, 126–130. doi:10.1016/j.forsciint.2005.12.011. |  |
| Cannabis lighting | 681 | Moosmann, B., Roth, N., and Auwaerter, V. (2015). Finding cannabinoids in hair does not prove cannabis consumption. *Sci. Rep.* 5. doi:10.1038/srep14906. |  |
| Cannabis lighting | 682 | Morgan, C. J. A., Freeman, T. P., Hindocha, C., Schafer, G., Gardner, C., and Curran, H. V. (2018). Individual and combined effects of acute delta-9-tetrahydrocannabinol and cannabidiol on psychotomimetic symptoms and memory function. *Transl. Psychiatry* 8. doi:10.1038/s41398-018-0191-x. |  |
| Cannabis lighting | 683 | Moscati, A., and Mezuk, B. (2014). Losing faith and finding religion: Religiosity over the life course and substance use and abuse. *Drug Alcohol Depend.* 136, 127–134. doi:10.1016/j.drugalcdep.2013.12.018. |  |
| Cannabis lighting | 684 | Moxon, D., and Waters, J. (2019). Sourcing illegal drugs as a hidden older user: the ideal of ?social supply? *DRUGS-EDUCATION Prev. POLICY* 26, 412–421. doi:10.1080/09687637.2018.1466866. |  |
| Cannabis lighting | 685 | Muller, S., and Gmel, G. (2002). Changes in the age of onset in cannabis use: results of the second Swiss Health Survey 1997. *SOZIAL-UND PRAVENTIVMEDIZIN* 47, 14–23. doi:10.1007/BF01318401. |  |
| Cannabis lighting | 686 | Murillo-Rodriguez, E., Arankowsky-Sandoval, G., Barbosa Rocha, N., Peniche-Amante, R., Barciela Veras, A., Machado, S., et al. (2018). Systemic Injections of Cannabidiol Enhance Acetylcholine Levels from Basal Forebrain in Rats. *Neurochem. Res.* 43, 1511–1518. doi:10.1007/s11064-018-2565-0. |  |
| Cannabis lighting | 687 | Murillo-Rodriguez, E., Millan-Aldaco, D., Palomero-Rivero, M., Mechoulam, R., and Drucker-Colin, R. (2006). Cannabidiol, a constituent of Cannabis sativa, modulates sleep in rats. *FEBS Lett.* 580, 4337–4345. doi:10.1016/j.febslet.2006.04.102. |  |
| Cannabis lighting | 688 | Murillo-Rodriguez, E., Millan-Aldaco, D., Palomero-Rivero, M., Mechoulam, R., and Drucker-Colin, R. (2008). The Nonpsychoactive Cannabis Constituent Cannabidiol Is a Wake-Inducing Agent. *Behav. Neurosci.* 122, 1378–1382. doi:10.1037/a0013278. |  |
| Cannabis lighting | 689 | Murillo-Rodriguez, E., Millan-Aldaco, D., Palomero-Rivero, M., Morales-Lara, D., Mechoulam, R., and Drucker-Colin, R. (2019). Cannabidiol Partially Blocks the Excessive Sleepiness in Hypocretin-deficient Rats: Preliminary Data. *CNS \& Neurol. Disord. TARGETS* 18, 705–712. doi:10.2174/1871527318666191021143300. |  |
| Cannabis lighting | 690 | Murillo-Rodriguez, E., Palomero-Rivero, M., Millan-Aldaco, D., Mechoulam, R., and Drucker-Colin, R. (2011). Effects on sleep and dopamine levels of microdialysis perfusion of cannabidiol into the lateral hypothalamus of rats. *LIFE Sci.* 88, 504–511. doi:10.1016/j.lfs.2011.01.013. |  |
| Cannabis lighting | 691 | Musshoff, F., and Madea, B. (2006). Review of biologic matrices (urine, blood, hair) as indicators of recent or ongoing cannabis use. *Ther. Drug Monit.* 28, 155–163. doi:10.1097/01.ftd.0000197091.07807.22. |  |
| Cannabis lighting | 692 | Mutlu, E., and Asicioglu, F. (2020). Abuse of prescribed psychoactive drugs. *ANADOLU Psikiyatr. DERGISI-ANATOLIAN J. PSYCHIATRY* 21, 195–202. doi:10.5455/apd.53469. |  |
| Cannabis lighting | 693 | Mutti-Packer, S., Collyer, B., and Hodgins, D. C. (2018). Perceptions of plain packaging and health warning labels for cannabis among young adults: findings from an experimental study. *BMC Public Health* 18. doi:10.1186/s12889-018-6247-2. |  |
| Cannabis lighting | 694 | Naji, L., Rosic, T., Dennis, B., Bhatt, M., Sanger, N., Hudson, J., et al. (2018). The association between cannabis use and suicidal behavior in patients with psychiatric disorders: an analysis of sex differences. *Biol. Sex Differ.* 9. doi:10.1186/s13293-018-0182-x. |  |
| Cannabis lighting | 695 | Namdar, D., Charuvi, D., Ajjampura, V., Mazuz, M., Ion, A., Kamara, I., et al. (2019). LED lighting affects the composition and biological activity of Cannabis sativa secondary metabolites. *Ind. Crops Prod.* 132, 177–185. doi:10.1016/j.indcrop.2019.02.016. |  |
| Cannabis lighting | 696 | Nazif-Munoz, J. I., Oulhote, Y., and Ouimet, M. C. (2020). The association between legalization of cannabis use and traffic deaths in Uruguay. *ADDICTION* 115, 1697–1706. doi:10.1111/add.14994. |  |
| Cannabis lighting | 697 | Ne’eman-Haviv, V., Bonny-Noach, H., Berkovitz, R., and Arieli, M. Attitudes, knowledge, and consumption of anabolic-androgenic steroids by recreational gym goers in Israel. *Sport Soc.* doi:10.1080/17430437.2020.1752672. |  |
| Cannabis lighting | 698 | Newmeyer, M. N., Swortwood, M. J., Taylor, M. E., Abulseoud, O. A., Woodward, T. H., and Huestis, M. A. (2017). Evaluation of divided attention psychophysical task performance and effects on pupil sizes following smoked, vaporized and oral cannabis administration. *J. Appl. Toxicol.* 37, 922–932. doi:10.1002/jat.3440. |  |
| Cannabis lighting | 699 | Nguyen, N., Barrington-Trimis, J. L., Urman, R., Cho, J., McConnell, R., Leventhal, A. M., et al. (2019). Past 30-day co-use of tobacco and marijuana products among adolescents and young adults in California. *Addict. Behav.* 98. doi:10.1016/j.addbeh.2019.106053. |  |
| Cannabis lighting | 700 | Ni, K., Wang, B., Link, A. R., and Sherman, S. E. (2020). Does Smoking Intensity Predict Cessation Rates? A Study of Light-Intermittent, Light-Daily, and Heavy Smokers Enrolled in Two Telephone-Based Counseling Interventions. *NICOTINE \& Tob. Res.* 22, 423–430. doi:10.1093/ntr/nty257. |  |
| Cannabis lighting | 701 | O’Brien, L. D., Wills, K. L., Segsworth, B., Dashney, B., Rock, E. M., Limebeer, C. L., et al. (2013). Effect of chronic exposure to rimonabant and phytocannabinoids on anxiety-like behavior and saccharin palatability. *Pharmacol. Biochem. Behav.* 103, 597–602. doi:10.1016/j.pbb.2012.10.008. |  |
| Cannabis lighting | 702 | Obafemi I, A., Kleinschmidt, K., Goto, C., and Fout, D. (2015). Cluster of Acute Toxicity from Ingestion of Synthetic Cannabinoid-Laced Brownies. *J. Med. Toxicol.* 11, 426–429. doi:10.1007/s13181-015-0482-z. |  |
| Cannabis lighting | 703 | OHLSSON, A., LINDGREN, J. E., WAHLEN, A., AGURELL, S., HOLLISTER, L. E., and GILLESPIE, H. K. (1982). SINGLE DOSE KINETICS OF DEUTERIUM LABELED DELTA-1-TETRAHYDROCANNABINOL IN HEAVY AND LIGHT CANNABIS USERS. *Biomed. Mass Spectrom.* 9, 6–10. doi:10.1002/bms.1200090103. |  |
| Cannabis lighting | 704 | Orriols, L., Delorme, B., Gadegbeku, B., Tricotel, A., Contrand, B., Laumon, B., et al. (2010). Prescription Medicines and the Risk of Road Traffic Crashes: A French Registry-Based Study. *PLOS Med.* 7. doi:10.1371/journal.pmed.1000366. |  |
| Cannabis lighting | 705 | Orsolini, L., Papanti, G. D., De Berardis, D., Guirguis, A., Corkery, J. M., and Schifano, F. (2017). The ``Endless Trip{’’} among the nPS Users: Psychopathology and Psychopharmacology in the Hallucinogen-Persisting Perception Disorder. A Systematic Review. *Front. PSYCHIATRY* 8. doi:10.3389/fpsyt.2017.00240. |  |
| Cannabis lighting | 706 | Oseyko, M., Sova, N., Lutsenko, M., and Kalyna, V. (2019). Chemical aspects of the composition of industrial hemp seed products. *Ukr. FOOD J.* 8, 544–559. doi:10.24263/2304-974X-2019-8-3-11. |  |
| Cannabis lighting | 707 | Pacheco-Colon, I., Coxe, S., Musser, E. D., Duperrouzel, J. C., Ross, J. M., and Gonzalez, R. (2018). Is Cannabis Use Associated with Various Indices of Motivation among Adolescents? *Subst. Use Misuse* 53, 1158–1169. doi:10.1080/10826084.2017.1400566. |  |
| Cannabis lighting | 708 | Pacifici, R., Pichini, S., Pellegrini, M., Rotolo, M. C., Giorgetti, R., Tagliabracci, A., et al. (2020). THC and CBD concentrations in blood, oral fluid and urine following a single and repeated administration of ``light cannabis{’’}. *Clin. Chem. Lab. Med.* 58, 682–689. doi:10.1515/cclm-2019-0119. |  |
| Cannabis lighting | 709 | Pacifici, R., Pichini, S., Pellegrini, M., Tittarelli, R., Pantano, F., Mannocchi, G., et al. (2019). Determination of cannabinoids in oral fluid and urine of ``light cannabis{’’} consumers: a pilot study. *Clin. Chem. Lab. Med.* 57, 238–243. doi:10.1515/cclm-2018-0566. |  |
| Cannabis lighting | 710 | Pacifico, D., Miselli, F., Micheler, M., Carboni, A., Ranalli, P., and Mandolino, G. (2006). Genetics and marker-assisted selection of the chemotype in Cannabis sativa L. *Mol. Breed.* 17, 257–268. doi:10.1007/s11032-005-5681-x. |  |
| Cannabis lighting | 711 | Padovano, H. T., and Miranda Jr., R. (2018). Subjective Cannabis Effects as Part of a Developing Disorder in Adolescents and Emerging Adults. *J. Abnorm. Psychol.* 127, 282–293. doi:10.1037/abn0000342. |  |
| Cannabis lighting | 712 | Pagotto, U., Marsicano, G., Cota, D., Lutz, B., and Pasquali, R. (2006). The emerging role of the endocannabinoid system in endocrine regulation and energy balance. *Endocr. Rev.* 27, 73–100. doi:10.1210/er.2005-0009. |  |
| Cannabis lighting | 713 | Paling, T., and Castello, J. V. (2017). Business cycle impacts on substance use of adolescents: A multi-country analysis. *Econ. Hum. Biol.* 27, 1–11. doi:10.1016/j.ehb.2017.04.005. |  |
| Cannabis lighting | 714 | Palmieri, S., Pellegrini, M., Ricci, A., Compagnone, D., and Lo Sterzo, C. (2020). Chemical Composition and Antioxidant Activity of Thyme, Hemp and Coriander Extracts: A Comparison Study of Maceration, Soxhlet, UAE and RSLDE Techniques. *FOODS* 9. doi:10.3390/foods9091221. |  |
| Cannabis lighting | 715 | Panlilio, L. V, Solinas, M., Matthews, S. A., and Goldberg, S. R. (2007). Previous exposure to THC alters the reinforcing efficacy and anxiety-related effects of cocaine in rats. *NEUROPSYCHOPHARMACOLOGY* 32, 646–657. doi:10.1038/sj.npp.1301109. |  |
| Cannabis lighting | 716 | Panlilio, L. V, Zanettini, C., Barnes, C., Solinas, M., and Goldberg, S. R. (2013). Prior Exposure to THC Increases the Addictive Effects of Nicotine in Rats. *NEUROPSYCHOPHARMACOLOGY* 38, 1198–1208. doi:10.1038/npp.2013.16. |  |
| Cannabis lighting | 717 | Paparelli, A., Di Forti, M., Morrison, P. D., and Murray, R. M. (2011). Drug-induced psychosis: how to avoid star gazing in schizophrenia research by looking at more obvious sources of light. *Front. Behav. Neurosci.* 5. doi:10.3389/fnbeh.2011.00001. |  |
| Cannabis lighting | 718 | Pardal, M. (2018a). The Belgian Cannabis Social Club landscape. *DRUGS AND ALCOHOL TODAY* 18, 80–89. doi:10.1108/DAT-09-2017-0051. |  |
| Cannabis lighting | 719 | Pardal, M. (2018b). “The difference is in the tomato at the end”: Understanding the motivations and practices of cannabis growers operating within Belgian Cannabis Social Clubs. doi:10.1016/j.drugpo.2018.02.016. |  |
| Cannabis lighting | 720 | Park, Y., Mackie, A. L., MacIsaac, S. A., and Gagnon, G. A. (2018). Photo-oxidation of 11-nor-9-carboxy-triangle(9)-tetrahydrocannabinol using medium-pressure UV and UV/H2O2 - a kinetic study. *Environ. Sci. Res. Technol.* 4, 1262–1271. doi:10.1039/c8ew00159f. |  |
| Cannabis lighting | 721 | Parrott, A. C., Hatton, N. P., Rowe, K. L., Watts, L. A., Donev, R., Kissling, C., et al. (2012). Adult attention deficit hyperactivity disorder and other psychiatric symptoms in recreational polydrug users. *Hum. Psychopharmacol. Exp.* 27, 209–216. doi:10.1002/hup.1271. |  |
| Cannabis lighting | 722 | Parrott, A. C., Milani, R. M., Parmar, R., and Turner, J. J. D. (2001). Recreational ecstasy/MDMA and other drug users from the UK and Italy: Psychiatric symptoms and psychobiological problems. *Psychopharmacology (Berl).* 159, 77–82. doi:10.1007/s002130100897. |  |
| Cannabis lighting | 723 | Patel, K. D., Davison, J. S., Pittman, Q. J., and Sharkey, K. A. (2010). Cannabinoid CB2 Receptors in Health and Disease. *Curr. Med. Chem.* 17, 1394–1410. doi:10.2174/092986710790980041. |  |
| Cannabis lighting | 724 | Paul, S. E., Hatoum, A. S., Fine, J. D., Johnson, E. C., Hansen, I., Karcher, N. R., et al. (2021). Associations Between Prenatal Cannabis Exposure and Childhood Outcomes Results From the ABCD Study. *JAMA PSYCHIATRY* 78, 64–76. doi:10.1001/jamapsychiatry.2020.2902. |  |
| Cannabis lighting | 725 | Pearson-Dennett, V., Flavel, S. C., Wilcox, R. A., Thewlis, D., Vogel, A. P., White, J. M., et al. (2014). Hand Function is Altered in Individuals with a History of Illicit Stimulant Use. *PLoS One* 9. doi:10.1371/journal.pone.0115771. |  |
| Cannabis lighting | 726 | Pedersen, W., and Bakken, A. (2016). Urban landscapes of adolescent substance use. *ACTA Sociol.* 59, 131–150. doi:10.1177/0001699315625448. |  |
| Cannabis lighting | 727 | Pellati, F., Borgonetti, V., Brighenti, V., Biagi, M., Benvenuti, S., and Corsi, L. (2018a). Cannabis sativa L. and Nonpsychoactive Cannabinoids: Their Chemistry and Role against Oxidative Stress, Inflammation, and Cancer. *Biomed Res. Int.* doi:10.1155/2018/1691428. |  |
| Cannabis lighting | 728 | Pellati, F., Brighenti, V., Sperlea, J., Marchetti, L., Bertelli, D., and Benvenuti, S. (2018b). New Methods for the Comprehensive Analysis of Bioactive Compounds in Cannabis sativa L. (hemp). *MOLECULES* 23. doi:10.3390/molecules23102639. |  |
| Cannabis lighting | 729 | Pepper, N., Zuniga, M. L., and Reed, M. B. (2020). Prevalence and correlates of ``popper{’’} (amyl nitrite inhalant) use among HIV-positive Latinos living in the US-Mexico border region. *J. Ethn. Subst. Abuse* 19, 435–452. doi:10.1080/15332640.2018.1540955. |  |
| Cannabis lighting | 730 | Pereira, L., Jesus Nunez-Iglesias, M., Maria Dominguez-Martis, E., Lopez-Ares, D., Gonzalez-Peteiro, M., and Novio, S. (2020). Nursing Students’ Knowledge and Attitudes Regarding Medical Marijuana: A Descriptive Cross-Sectional Study. *Int. J. Environ. Res. Public Health* 17. doi:10.3390/ijerph17072492. |  |
| Cannabis lighting | 731 | Petit, J., Salentijn, E. M. J., Paulo, M.-J., Denneboom, C., and Trindade, L. M. (2020). Genetic Architecture of Flowering Time and Sex Determination in Hemp (Cannabis sativa L.): A Genome-Wide Association Study. *Front. Plant Sci.* 11. doi:10.3389/fpls.2020.569958. |  |
| Cannabis lighting | 732 | Pichini, S., Garcia-Algar, O., Alvarez, A., Gottardi, M., Marchei, E., Svaizer, F., et al. (2014). Assessment of Unsuspected Exposure to Drugs of Abuse in Children from a Mediterranean City by Hair Testing. *Int. J. Environ. Res. Public Health* 11, 2288–2298. doi:10.3390/ijerph110202288. |  |
| Cannabis lighting | 733 | Pichini, S., Mannocchi, G., Berretta, P., Zaami, S., Pirani, F., Pacifici, R., et al. (2020). Delta 9-Tetrahydrocannabinol and Cannabidiol Time Courses in the Sera of ``Light Cannabis{’’} Smokers: Discriminating Light Cannabis Use from Illegal and Medical Cannabis Use. *Ther. Drug Monit.* 42, 151–156. doi:10.1097/FTD.0000000000000683. |  |
| Cannabis lighting | 734 | Piette, C., Cui, Y., Gervasi, N., and Venance, L. (2020). Lights on Endocannabinoid-Mediated Synaptic Potentiation. *Front. Mol. Neurosci.* 13. doi:10.3389/fnmol.2020.00132. |  |
| Cannabis lighting | 735 | Pinto, J. V., Medeiros, L. S., da Rosa, G. S., de Oliveira, C. E., de Souza Crippa, J. A., Passosa, I. C., et al. (2019). The prevalence and clinical correlates of cannabis use and cannabis use disorder among patients with bipolar disorder: A systematic review with meta-analysis and meta-regression. *Neurosci. Biobehav. Rev.* 101, 78–84. doi:10.1016/j.neubiorev.2019.04.004. |  |
| Cannabis lighting | 736 | Piotrowska-Cyplik, A., and Czarnecki, Z. (2003). Phytoextraction of heavy metals by hemp during anaerobic sewage sludge management in the non-industrial sites. *POLISH J. Environ. Stud.* 12, 779–784. |  |
| Cannabis lighting | 737 | Pletcher, M. J., Vittinghoff, E., Kalhan, R., Richman, J., Safford, M., Sidney, S., et al. (2012). Association Between Marijuana Exposure and Pulmonary Function Over 20 Years. *JAMA-JOURNAL Am. Med. Assoc.* 307, 173–181. doi:10.1001/jama.2011.1961. |  |
| Cannabis lighting | 738 | Pope, H. G., Jacobs, A., Mialet, J. P., YurgelunTodd, D., and Gruber, S. (1997). Evidence for a sex-specific residual effect of cannabis on visuospatial memory. *Psychother. Psychosom.* 66, 179–184. |  |
| Cannabis lighting | 739 | Pope, H. G., and YurgelunTodd, D. (1996). The residual cognitive effects of heavy marijuana use in college students. *JAMA-JOURNAL Am. Med. Assoc.* 275, 521–527. doi:10.1001/jama.275.7.521. |  |
| Cannabis lighting | 740 | Porath-Waller, A. J., and Beirness, D. J. (2010). Simplifying the Process for Identifying Drug Combinations by Drug Recognition Experts. *TRAFFIC Inj. Prev.* 11, 453–459. doi:10.1080/15389588.2010.489199. |  |
| Cannabis lighting | 741 | Porath-Waller, A. J., Beirness, D. J., and Beasley, E. E. (2009). Toward a More Parsimonious Approach to Drug Recognition Expert Evaluations. *TRAFFIC Inj. Prev.* 10, 513–518. doi:10.1080/15389580903191617. |  |
| Cannabis lighting | 742 | Porath, A. J., and Beirness, D. J. Predicting categories of drugs used by suspected drug-impaired drivers using the Drug Evaluation and Classification Program tests. *TRAFFIC Inj. Prev.* doi:10.1080/15389588.2018.1562178. |  |
| Cannabis lighting | 743 | Potter, D. J., and Duncombe, P. (2012). The Effect of Electrical Lighting Power and Irradiance on Indoor-Grown Cannabis Potency and Yield. *J. Forensic Sci.* 57, 618–622. doi:10.1111/j.1556-4029.2011.02024.x. |  |
| Cannabis lighting | 744 | Potvin, S., Stip, E., and Roy, J. Y. (2003). Schizophrenia and addiction: An evaluation of the self-medication hypothesis. *Enceph. Psychiatr. Clin. Biol. Ther.* 29, 193–203. |  |
| Cannabis lighting | 745 | Punja, Z. K., and Holmes, J. E. (2020). Hermaphroditism in Marijuana (Cannabis sativaL.) Inflorescences - Impact on Floral Morphology, Seed Formation, Progeny Sex Ratios, and Genetic Variation. *Front. Plant Sci.* 11. doi:10.3389/fpls.2020.00718. |  |
| Cannabis lighting | 746 | Raikos, V., Konstantinidi, V., and Duthie, G. (2015). Processing and storage effects on the oxidative stability of hemp (Cannabis sativa L.) oil-in-water emulsions. *Int. J. FOOD Sci. Technol.* 50, 2316–2322. doi:10.1111/ijfs.12896. |  |
| Cannabis lighting | 747 | Rajavashisth, T. B., Shaheen, M., Norris, K. C., Pan, D., Sinha, S. K., Ortega, J., et al. (2012). Decreased prevalence of diabetes in marijuana users: cross-sectional data from the National Health and Nutrition Examination Survey (NHANES) III. *BMJ Open* 2. doi:10.1136/bmjopen-2011-000494. |  |
| Cannabis lighting | 748 | Ramzy, V., and Priefer, R. (2021). THC detection in the breath. *Talanta* 222. doi:10.1016/j.talanta.2020.121528. |  |
| Cannabis lighting | 749 | Rana, S. S., and Gupta, M. K. (2020). Isolation of nanocellulose from hemp (Cannabis sativa) fibers by chemo-mechanical method and its characterization. *Polym. Compos.* 41, 5257–5268. doi:10.1002/pc.25791. |  |
| Cannabis lighting | 750 | Reece, A. S., and Hulse, G. K. (2019). Impacts of cannabinoid epigenetics on human development: reflections on Murphy et. al. `cannabinoid exposure and altered DNA methylation in rat and human sperm’ epigenetics 2018; 13: 1208-1221. *EPIGENETICS* 14, 1041–1056. doi:10.1080/15592294.2019.1633868. |  |
| Cannabis lighting | 751 | Reininghaus, U., Bohnke, J. R., Hosang, G., Farmer, A., Burns, T., McGuffin, P., et al. (2016). Evaluation of the validity and utility of a transdiagnostic psychosis dimension encompassing schizophrenia and bipolar disorder. *Br. J. PSYCHIATRY* 209, 107–113. doi:10.1192/bjp.bp.115.167882. |  |
| Cannabis lighting | 752 | Rezaie, A., Pezeshki, A., Zadfattah, B., Nazeri, M., Ahmadizadeh, C., and Mohammadi, B. (2012). Comparative Study of Sedation, Pre-Anesthetic and Anti-Anxiety Effects of Hemp Seed Extract and Diazepam in Rats. *J. Anim. Vet. Adv.* 11, 2148–2151. doi:10.3923/javaa.2012.2148.2151. |  |
| Cannabis lighting | 753 | Rich, L. N., Ferguson, E., Baker, A. D., and Chappell, E. (2020). A review of the potential impacts of artificial lights on fish and wildlife and how this may apply to cannabis cultivation. *Calif. FISH GAME* 106, 75–91. |  |
| Cannabis lighting | 754 | Richards, B. L., Whittle, S. L., and Buchbinder, R. (2012). Neuromodulators for pain management in rheumatoid arthritis. *COCHRANE DATABASE Syst. Rev.* doi:10.1002/14651858.CD008921.pub2. |  |
| Cannabis lighting | 755 | Roberts, K. (2018). Medicinal cannabis gets green light. *Chem. Ind.* 82, 10. |  |
| Cannabis lighting | 756 | Rock, E. M., Limebeer, C. L., Petrie, G. N., Williams, L. A., Mechoulam, R., and Parker, L. A. (2017). Effect of prior foot shock stress and Delta(9)-tetrahydrocannabinol, cannabidiolic acid, and cannabidiol on anxiety-like responding in the light-dark emergence test in rats. *Psychopharmacology (Berl).* 234, 2207–2217. doi:10.1007/s00213-017-4626-5. |  |
| Cannabis lighting | 757 | Rodriguez-Almaraz, J.-E., Chang, S., Clarke, J., Oberheim-Bush, N. A., Taylor, J., Buerki, R., et al. (2020). A systematic review and meta-analysis examining the effects of cannabis and its derivatives in adults with malignant CNS tumors. *NEURO-ONCOLOGY Pract.* 7, 376–383. doi:10.1093/nop/npaa013. |  |
| Cannabis lighting | 758 | Roesch, M. (2008). New aspects of agriculture and diet of the early medieval period in central Europe: waterlogged plant material from sites in south-western Germany. *Veg. Hist. Archaeobot.* 17, S225–S238. doi:10.1007/s00334-008-0184-4. |  |
| Cannabis lighting | 759 | Roffman, R. (2016). Legalization of cannabis in Washington State: how is it going? *ADDICTION* 111, 1139–1140. doi:10.1111/add.13247. |  |
| Cannabis lighting | 760 | Romero, P., Peris, A., Vergara, K., and Matus, J. T. (2020). Comprehending and improving cannabis specialized metabolism in the systems biology era. *PLANT Sci.* 298. doi:10.1016/j.plantsci.2020.110571. |  |
| Cannabis lighting | 761 | Ronen, A., Chassidim, H. S., Gershon, P., Parmet, Y., Rabinovich, A., Bar-Hamburger, R., et al. (2010). The effect of alcohol, THC and their combination on perceived effects, willingness to drive and performance of driving and non-driving tasks. *Accid. Anal. Prev.* 42, 1855–1865. doi:10.1016/j.aap.2010.05.006. |  |
| Cannabis lighting | 762 | Rosenberg, E. C., Patra, P. H., and Whalley, B. J. (2017). Therapeutic effects of cannabinoids in animal models of seizures, epilepsy, epileptogenesis, and epilepsy-related neuroprotection. *EPILEPSY Behav.* 70, 319–327. doi:10.1016/j.yebeh.2016.11.006. |  |
| Cannabis lighting | 763 | Roser, P., Della, B., Norra, C., Uhl, I., Bruene, M., and Juckel, G. (2010). Auditory mismatch negativity deficits in long-term heavy cannabis users. *Eur. Arch. Psychiatry Clin. Neurosci.* 260, 491–498. doi:10.1007/s00406-010-0097-y. |  |
| Cannabis lighting | 764 | Rothschild, M., Bergstrom, G., and Wangberg, S. A. (2005). Cannabis sativa: volatile compounds from pollen and entire male and female plants of two variants, Northern Lights and Hawaian Indica. *Bot. J. Linn. Soc.* 147, 387–397. doi:10.1111/j.1095-8339.2005.00417.x. |  |
| Cannabis lighting | 765 | Rounsaville, B. J. (2007). DSM-V research agenda: Substance abuse/psychosis comorbidity. *Schizophr. Bull.* 33, 947–952. doi:10.1093/schbul/sbm054. |  |
| Cannabis lighting | 766 | Rovai, L., Maremmani, A. G. I., Pacini, M., Pani, P. P., Rugani, F., Lamanna, F., et al. (2013). Negative dimension in psychiatry. Amotivational syndrome as a paradigm of negative symptoms in substance abuse. *Riv. Psichiatr.* 48, 1–9. |  |
| Cannabis lighting | 767 | Rugani, F., Paganin, W., Maremmani I, A. G., Perugi, G., and Maremmani, I. (2019). Towards a specific psychopathology of Substance Use Disorder: Comparison between Heroin Use Disorder and chronic psychotic patients. *HEROIN Addict. Relat. Clin. Probl.* 21, 53–59. |  |
| Cannabis lighting | 768 | Rylander, M., Valdez, C., and Nussbaum, A. M. (2014). Does the legalization of medical marijuana increase completed suicide? *Am. J. Drug Alcohol Abuse* 40, 269–273. doi:10.3109/00952990.2014.910520. |  |
| Cannabis lighting | 769 | Sagud, M., Vuksan-Cusa, B., Jaksic, N., Mihaljevic-Peles, A., Zivkovic, M., Vlatkovic, S., et al. (2018). Nicotine dependence in Croatian male inpatients with schizophrenia. *BMC Psychiatry* 18. doi:10.1186/s12888-018-1606-1. |  |
| Cannabis lighting | 770 | Sagy, I., Peleg-Sagy, T., Barski, L., Zeller, L., and Jotkowitz, A. (2018). Ethical issues in medical cannabis use. *Eur. J. Intern. Med.* 49, 20–22. doi:10.1016/j.ejim.2018.01.016. |  |
| Cannabis lighting | 771 | Saliba Moimaz, S. A., Zina, L. G., Saliba, O., and Saliba Garbin, C. A. (2009). Smoking and Periodontal Disease: Clinical Evidence for an Association. *Oral Health Prev. Dent.* 7, 369–376. |  |
| Cannabis lighting | 772 | Samarut, E., Nixon, J., Kundap, U. P., Drapeau, P., and Ellis, L. D. (2019). Single and Synergistic Effects of Cannabidiol and Delta-9-Tetrahydrocannabinol on Zebrafish Models of Neuro-Hyperactivity. *Front. Pharmacol.* 10. doi:10.3389/fphar.2019.00226. |  |
| Cannabis lighting | 773 | Sanford, A. E., Castillo, E., and Gannon, R. L. (2008). Cannabinoids and hamster circadian activity rhythms. *BRAIN Res.* 1222, 141–148. doi:10.1016/j.brainres.2008.05.048. |  |
| Cannabis lighting | 774 | Sarne, Y., and Mechoulam, R. (2005). Cannabinoids: Between Neuroprotection and Neurotoxicity. *CNS \& Neurol. Disord. TARGETS* 4, 677–684. doi:10.2174/156800705774933005. |  |
| Cannabis lighting | 775 | Sasco, A. J., Merrill, R. M., Dari, I., Benhaim-Luzon, V., Carriot, F., Cann, C. I., et al. (2002). A case-control study of lung cancer in Casablanca, Morocco. *CANCER CAUSES Control* 13, 609–616. doi:10.1023/A:1019504210176. |  |
| Cannabis lighting | 776 | Schauer, G. L., Berg, C. J., Kegler, M. C., Donovan, D. M., and Windle, M. (2016a). Differences in Tobacco Product Use Among Past Month Adult Marijuana Users and Nonusers: Findings From the 2003-2012 National Survey on Drug Use and Health. *NICOTINE Tob. Res.* 18, 281–288. doi:10.1093/ntr/ntv093. |  |
| Cannabis lighting | 777 | Schauer, G. L., King, B. A., Bunnell, R. E., Promoff, G., and McAfee, T. A. (2016b). Toking, Vaping, and Eating for Health or Fun Marijuana Use Patterns in Adults, US, 2014. *Am. J. Prev. Med.* 50, 1–8. doi:10.1016/j.amepre.2015.05.027. |  |
| Cannabis lighting | 778 | Schleicher, E. M., Ott, F. W., Mueller, M., Silcher, B., Sichler, M. E., Loew, M. J., et al. (2019). Prolonged Cannabidiol Treatment Lacks on Detrimental Effects on Memory, Motor Performance and Anxiety in C57BL/6J Mice. *Front. Behav. Neurosci.* 13. doi:10.3389/fnbeh.2019.00094. |  |
| Cannabis lighting | 779 | Schnelle, M., Grotenhermen, F., Reif, M., and Gorter, R. W. (1999). Results of a standardized survey on the medical use of cannabis products in the German-speaking area. *Forsch. Komplementarmed.* 6, 28–36. doi:10.1159/000057154. |  |
| Cannabis lighting | 780 | Schramm-Sapyta, N. L., Cha, Y. M., Chaudhry, S., Wilson, W. A., Swartzwelder, H. S., and Kuhn, C. M. (2007). Differential anxiogenic, aversive, and locomotor effects of THC in adolescent and adult rats. *Psychopharmacology (Berl).* 191, 867–877. doi:10.1007/s00213-006-0676-9. |  |
| Cannabis lighting | 781 | Schuckit, M. A., Smith, T. L., Shafir, A., Clausen, P., Danko, G., Goncalves, P. D., et al. (2017). Predictors of Patterns of Alcohol-Related Blackouts Over Time in Youth From the Collaborative Study of the Genetics of Alcoholism: The Roles of Genetics and Cannabis. *J. Stud. Alcohol Drugs* 78, 39–48. |  |
| Cannabis lighting | 782 | Schurhoff, F., Fond, G., Berna, F., Bulzacka, E., Godin, O., Boyer, L., et al. (2019). The 10-year findings from the FondaMental Academic Center of Expertise for Schizophrenia (FACE-SZ): Review and recommendations for clinical practice. *Enceph. Psychiatr. Clin. Biol. Ther.* 45, 9–14. doi:10.1016/j.encep.2018.07.007. |  |
| Cannabis lighting | 783 | Schwantes-An, T.-H., Zhang, J., Chen, L.-S., Hartz, S. M., Culverhouse, R. C., Chen, X., et al. (2016). Association of the OPRM1 Variant rs1799971 (A118G) with Non-Specific Liability to Substance Dependence in a Collaborative de novo Meta-Analysis of European-Ancestry Cohorts. *Behav. Genet.* 46, 151–169. doi:10.1007/s10519-015-9737-3. |  |
| Cannabis lighting | 784 | Schwarz, S., Leweling, H., Sagstetter, B.-U., and Meinck, H.-M. (2008). Complementary and alternative therapies in multiple sclerosis. *PSYCHOPHARMAKOTHERAPIE* 15, 190–199. |  |
| Cannabis lighting | 785 | Schwitzer, T., Schwan, R., Angioi-Duprez, K., Giersch, A., Lalanne, L., Albuisson, E., et al. (2018). Delayed bipolar and ganglion cells neuroretinal processing in regular cannabis users: The retina as a relevant site to investigate brain synaptic transmission dysfunctions. *J. Psychiatr. Res.* 103, 75–82. doi:10.1016/j.jpsychires.2018.04.021. |  |
| Cannabis lighting | 786 | Scott, C., and Punja, Z. K. Evaluation of disease management approaches for powdery mildew on Cannabis sativa L. (marijuana) plants. *Can. J. PLANT Pathol.* doi:10.1080/07060661.2020.1836026. |  |
| Cannabis lighting | 787 | Scott, J. C., Rosen, A. F. G., Moore, T. M., Roalf, D. R., Satterthwaite, T. D., Calkins, M. E., et al. (2019). Cannabis use in youth is associated with limited alterations in brain structure. *NEUROPSYCHOPHARMACOLOGY* 44, 1362–1369. doi:10.1038/s41386-019-0347-2. |  |
| Cannabis lighting | 788 | Scuro, J. (2019). DRUGS IN THE URUGUAY-BRAZIL BORDER: PERCEPTIONS OF LEGAL AND SECURITY AGENTS DURING THE IMPLEMENTATION OF THE LAW FOR THE CANNABIS MARKET REGULATION IN URUGUAY. *Cult. Y Drog.* 27, 39–61. doi:10.17151/culdr.2019.24.27.3. |  |
| Cannabis lighting | 789 | Segal-Gavish, H., Gazit, N., Barhum, Y., Ben-Zur, T., Taler, M., Hornfeld, S. H., et al. (2017). BDNF overexpression prevents cognitive deficit elicited by adolescent cannabis exposure and host susceptibility interaction. *Hum. Mol. Genet.* 26, 2462–2471. doi:10.1093/hmg/ddx139. |  |
| Cannabis lighting | 790 | Seillier, A., Martinez, A. A., and Giuffrida, A. (2020). Differential effects of Delta 9-tetrahydrocannabinol dosing on correlates of schizophrenia in the sub-chronic PCP rat model. *PLoS One* 15. doi:10.1371/journal.pone.0230238. |  |
| Cannabis lighting | 791 | Sevigny, E. L., Pacula, R. L., and Heaton, P. (2014). The effects of medical marijuana laws on potency. *Int. J. DRUG POLICY* 25, 308–319. doi:10.1016/j.drugpo.2014.01.003. |  |
| Cannabis lighting | 792 | Sharma, U. R., and Sharma, N. (2021). Green Synthesis, Anti-cancer and Corrosion Inhibition Activity of Cr2O3 Nanoparticles. *BIOINTERFACE Res. Appl. Chem.* 11, 8402–8412. doi:10.33263/BRIAC111.84028412. |  |
| Cannabis lighting | 793 | Shinwari, Z. K., Tanveer, M., Yusuf, O., Perveen, A., and Khan, M. (2015). PROTEIN ESTIMATION AND PALYNLOGICAL STUDIES OF CANNABIS SATIVA L. POLLEN IN RELATION TO RESPIRATORY ALLERGIES. *PAKISTAN J. Bot.* 47, 1517–1520. |  |
| Cannabis lighting | 794 | Shuvo, I. I., Rahman, M., Vahora, T., Morrison, J., DuCharme, S., and Choo-Smith, L.-P. (2020). Producing light-weight bast fibers from canola biomass for technical textiles. *Text. Res. J.* 90, 1311–1325. doi:10.1177/0040517519886636. |  |
| Cannabis lighting | 795 | Simmons, R. D., Ponsonby, A. L., van der Mei, I. A. F., and Sheridan, P. (2004). What affects your MS? Responses to an anonymous, Internet-based epidemiological survey. *Mult. Scler.* 10, 202–211. doi:10.1191/1352458504ms1006oa. |  |
| Cannabis lighting | 796 | Simons-Linares, C. R., Elkhouly, M. A., and Salazar, M. J. (2019). Drug-Induced Acute Pancreatitis in Adults An Update. *Pancreas* 48, 1263–1273. doi:10.1097/MPA.0000000000001428. |  |
| Cannabis lighting | 797 | Singh, P., Pandit, S., Garnaes, J., Tunjic, S., Mokkapati, V. R. S. S., Sultan, A., et al. (2018a). Green synthesis of gold and silver nanoparticles from Cannabis sativa (industrial hemp) and their capacity for biofilm inhibition. *Int. J. Nanomedicine* 13, 3571–3591. doi:10.2147/IJN.S157958. |  |
| Cannabis lighting | 798 | Singh, T., Jyoti, K., Patnaik, A., Singh, A., and Chauhan, S. C. (2018b). Spectroscopic, microscopic characterization of Cannabis sativa leaf extract mediated silver nanoparticles and their synergistic effect with antibiotics against human pathogen. *ALEXANDRIA Eng. J.* 57, 3043–3051. doi:10.1016/j.aej.2018.04.002. |  |
| Cannabis lighting | 799 | Sinnasse, P., and Tereno, S. (2017). Alcohol, cannabis and tobacco consumptions and attachment quality. *Ann. Med. Psychol. (Paris).* 175, 370–375. doi:10.1016/j.amp.2016.11.006. |  |
| Cannabis lighting | 800 | Slawson, G., Milloy, M.-J., Balneaves, L., Simo, A., Guillemi, S., Hogg, R., et al. (2015). High-Intensity Cannabis Use and Adherence to Antiretroviral Therapy Among People Who Use Illicit Drugs in a Canadian Setting. *AIDS Behav.* 19, 120–127. doi:10.1007/s10461-014-0847-3. |  |
| Cannabis lighting | 801 | Smith, D. M., O’Connor, R. J., Collins, R. L., Hyland, A. J., and Kozlowski, L. T. (2019). Correlates of smoker identity among intermittent and light daily young adult smokers: Findings from Wave 1 of the Population Assessment of Tobacco and Health (PATH) Study. *Addict. Behav.* 98. doi:10.1016/j.addbeh.2019.106034. |  |
| Cannabis lighting | 802 | Snyder, K. E., Pittard, C. M., Fowler, A., and Watson, C. T. (2020). ``Epic-Genetics{’’}: An Exploration of Preservice Helping Professionals’ (Mis)Understanding of Epigenetic Influences on Human Development. *Teach. \& Learn. Inq. ISSOTL J.* 8, 122–137. doi:10.20343/teachlearninqu.8.1.9. |  |
| Cannabis lighting | 803 | Solomon, R., and Chamberlain, E. (2014). Canada’s New Drug-Impaired Driving Law: The Need to Consider Other Approaches. *TRAFFIC Inj. Prev.* 15, 685–693. doi:10.1080/15389588.2013.871004. |  |
| Cannabis lighting | 804 | Sommano, S. R., Chittasupho, C., Ruksiriwanich, W., and Jantrawut, P. (2020). The Cannabis Terpenes. *MOLECULES* 25. doi:10.3390/molecules25245792. |  |
| Cannabis lighting | 805 | Spencer, J. R., Chohan, T. W., Karl, T., and Arnold, J. C. (2013). Female Neuregulin 1 Heterozygous Mice Require Repeated Exposure to Delta(9)-Tetrahydrocannabinol to Alter Sensorimotor Gating Function. *Pharmacopsychiatry* 46, 286–291. doi:10.1055/s-0033-1355390. |  |
| Cannabis lighting | 806 | Sperduti, A., Giuliani, M. R., Guida, G., Petrone, P. P., Rossi, P. F., Vaccaro, S., et al. (2018). Tooth grooves, occlusal striations, dental calculus, and evidence for fiber processing in an Italian eneolithic/bronze age cemetery. *Am. J. Phys. Anthropol.* 167, 234–243. doi:10.1002/ajpa.23619. |  |
| Cannabis lighting | 807 | Srinivasababu, N. (2014). Assessing the Mechanical Performance Cannabis Sativa Composites - Reinforced with Long Time Dried fibre. in *12TH GLOBAL CONGRESS ON MANUFACTURING AND MANAGEMENT (GCMM - 2014)* Procedia Engineering., ed. Xavior, MA and Yarlagadda, PKDV, 986–993. doi:10.1016/j.proeng.2014.12.375. |  |
| Cannabis lighting | 808 | St Pierre, M., Russo, E. B., and Walsh, Z. (2020). No Evidence of Altered Reactivity to Experimentally Induced Pain Among Regular Cannabis Users. *Clin. J. Pain* 36, 589–593. doi:10.1097/AJP.0000000000000844. |  |
| Cannabis lighting | 809 | Stone, A. L. (2020). Adolescent Cannabis Use and Perceived Social Norm Trends Pre- and Post-Implementation of Washington State’s Liberalized Recreational Cannabis Policy: Healthy Youth Survey, 2008-2018. *Prev. Sci.* 21, 772–783. doi:10.1007/s11121-020-01136-0. |  |
| Cannabis lighting | 810 | Stone, A. L., Storr, C. L., and Anthony, J. C. (2006). Evidence for a hallucinogen dependence syndrome developing soon after onset of hallucinogen use during adolescence. *Int. J. Methods Psychiatr. Res.* 15, 116–130. doi:10.1002/mpr.188. |  |
| Cannabis lighting | 811 | Stormshak, E. A., Caruthers, A. S., Gau, J. M., and Winter, C. (2019). The Impact of Recreational Marijuana Legalization on Rates of Use and Behavior: A 10-Year Comparison of Two Cohorts From High School to Young Adulthood. *Psychol. Addict. Behav.* 33, 595–602. doi:10.1037/adb0000508. |  |
| Cannabis lighting | 812 | Struik, M. L., Yazulla, S., and Kamermans, M. (2006). Cannabinoid agonist WIN 55212-2 speeds up the cone response to light offset in goldfish retina. *Vis. Neurosci.* 23, 285–293. doi:10.1017/S0952523806232127. |  |
| Cannabis lighting | 813 | Subbaraman, M. S., Barnett, S. B., and Karriker-Jaffe, K. J. (2019). Risks Associated with Mid level Cannabis Use Among People Treated for Alcohol Use Disorder. *Alcohol. Exp. Res.* 43, 690–694. doi:10.1111/acer.13973. |  |
| Cannabis lighting | 814 | Suomela, J. A., Vajanto, K., and Raisanen, R. (2018). Seeking Nettle Textiles - Utilizing a Combination of Microscopic Methods for Fibre Identification. *Stud. Conserv.* 63, 412–422. doi:10.1080/00393630.2017.1410956. |  |
| Cannabis lighting | 815 | Sznitman, S. R. (2007). An examination of the normalisation of cannabis use among 9th grade school students in Sweden and Switzerland. *Addict. Res. Theory* 15, 601–616. doi:10.1080/16066350701433233. |  |
| Cannabis lighting | 816 | Sznitman, S. R., and Zolotov, Y. (2015). Cannabis for Therapeutic Purposes and public health and safety: A systematic and critical review. *Int. J. DRUG POLICY* 26, 20–29. doi:10.1016/j.drugpo.2014.09.005. |  |
| Cannabis lighting | 817 | Szulakowska, A., and Milnerowicz, H. (2007). Cannabis sativa in the light of scientific research. *Adv. Clin. Exp. Med.* 16, 807–815. |  |
| Cannabis lighting | 818 | Szyliowicz, D., and Hilsenrath, P. (2019). Medical Marijuana Knowledge and Attitudes: A Survey of the California Pharmacists Association. *J. Prim. CARE COMMUNITY Heal.* 10. doi:10.1177/2150132719831871. |  |
| Cannabis lighting | 819 | Taghinasab, M., and Jabaji, S. (2020). Cannabis Microbiome and the Role of Endophytes in Modulating the Production of Secondary Metabolites: An Overview. *MICROORGANISMS* 8. doi:10.3390/microorganisms8030355. |  |
| Cannabis lighting | 820 | Tait, R. J., Mackinnon, A., and Christensen, H. (2011). Cannabis use and cognitive function: 8-year trajectory in a young adult cohort. *ADDICTION* 106, 2195–2203. doi:10.1111/j.1360-0443.2011.03574.x. |  |
| Cannabis lighting | 821 | Takakuwa, K. M. (2020). A history of the Society of Cannabis Clinicians and its contributions and impact on the US medical cannabis movement. *Int. J. DRUG POLICY* 79. doi:10.1016/j.drugpo.2020.102749. |  |
| Cannabis lighting | 822 | Taneri, B., Ambrosino, E., van Os, J., and Brand, A. (2012). A new public health genomics model for common complex diseases, with an application to common behavioral disorders. *Per. Med.* 9, 29–38. doi:10.2217/PME.11.81. |  |
| Cannabis lighting | 823 | Tang, K., Fracasso, A., Struik, P. C., Yin, X., and Amaducci, S. (2018). Water- and Nitrogen-Use Efficiencies of Hemp (Cannabis sativa L.) Based on Whole-Canopy Measurements and Modeling. *Front. Plant Sci.* 9. doi:10.3389/fpls.2018.00951. |  |
| Cannabis lighting | 824 | Tang, K., Struik, P. C., Amaducci, S., Stomph, T.-J., and Yin, X. (2017). Hemp (Cannabis sativa L.) leaf photosynthesis in relation to nitrogen content and temperature: implications for hemp as a bio-economically sustainable crop. *Glob. Chang. Biol. BIOENERGY* 9, 1573–1587. doi:10.1111/gcbb.12451. |  |
| Cannabis lighting | 825 | Taylor, M., Lees, R., Henderson, G., Lingford-Hughes, A., Macleod, J., Sullivan, J., et al. (2017). Comparison of cannabinoids in hair with self-reported cannabis consumption in heavy, light and non-cannabis users. *Drug Alcohol Rev.* 36, 220–226. doi:10.1111/dar.12412. |  |
| Cannabis lighting | 826 | Techen, N., Chandra, S., Lata, H., ElSohly, M. A., and Khan, I. A. (2010). Genetic Identification of Female Cannabis sativa Plants at Early Developmental Stage. *Planta Med.* 76, 1938–1939. doi:10.1055/s-0030-1249978. |  |
| Cannabis lighting | 827 | Thames, A. D., Mahmood, Z., Burggren, A. C., Karimian, A., and Kuhn, T. P. (2016). Combined effects of HIV and marijuana use on neurocognitive functioning and immune status. *AIDS CARE-PSYCHOLOGICAL SOCIO-MEDICAL Asp. AIDS/HIV* 28, 628–632. doi:10.1080/09540121.2015.1124983. |  |
| Cannabis lighting | 828 | Thapa, D., Samadi, N., Patel, N., and Tabatabaei, N. (2020a). Enhancement of Sensitivity and Detection Limit of Lateral Flow Immunoassays using Lock-In Thermography. in *BIOPHOTONICS IN POINT-OF-CARE* Proceedings of SPIE., ed. Canva, MT and Giannetti, A and Altug, H and Moreau, J doi:10.1117/12.2555274. |  |
| Cannabis lighting | 829 | Thapa, D., Samadi, N., Patel, N., and Tabatabaei, N. (2020b). Thermographic detection and quantification of THC in oral fluid at unprecedented low concentrations. *Biomed. Opt. Express* 11, 2178–2190. doi:10.1364/BOE.388990. |  |
| Cannabis lighting | 830 | Thirthalli, J., and Benegal, V. (2006). Psychosis among substance users. *Curr. Opin. Psychiatry* 19, 239–245. doi:10.1097/01.yco.0000218593.08313.fd. |  |
| Cannabis lighting | 831 | Thiruchselvam, T., Malik, S., and Le Foll, B. (2017). A review of positron emission tomography studies exploring the dopaminergic system in substance use with a focus on tobacco as a co-variate. *Am. J. Drug Alcohol Abuse* 43, 197–214. doi:10.1080/00952990.2016.1257633. |  |
| Cannabis lighting | 832 | Thomasius, R., Zapletalova, P., Petersen, K., Buchert, R., Andresen, B., Wartberg, L., et al. (2006). Mood, cognition and serotonin transporter availability in current and former ecstasy (MDMA) users: the longitudinal perspective. *J. Psychopharmacol.* 20, 211–225. doi:10.1177/026881106059486. |  |
| Cannabis lighting | 833 | Thygesen, L. G., and Ander, P. (2005). Quantification of dislocations in spruce pulp and hemp fibres using polarized light microscopy and image analysis. *Nord. PULP Pap. Res. J.* 20, 64–71. doi:10.3183/npprj-2005-20-01-p064-071. |  |
| Cannabis lighting | 834 | Thygesen, L. G., Bilde-Sorensen, J. B., and Hoffmeyer, P. (2006). Visualisation of dislocations in hemp fibres: A comparison between scanning electron microscopy (SEM) and polarized light microscopy (PLM). *Ind. Crops Prod.* 24, 181–185. doi:10.1016/j.indcrop.2006.03.009. |  |
| Cannabis lighting | 835 | Thygesen, L. G., Eder, M., and Burgert, I. (2007). Dislocations in single hemp fibres-investigations into the relationship of structural distortions and tensile properties at the cell wall level. *J. Mater. Sci.* 42, 558–564. doi:10.1007/s10853-006-1113-5. |  |
| Cannabis lighting | 836 | Thygesen, L. G., and Gierlinger, N. (2013). The molecular structure within dislocations in Cannabis sativa fibres studied by polarised Raman microspectroscopy. *J. Struct. Biol.* 182, 219–225. doi:10.1016/j.jsb.2013.03.010. |  |
| Cannabis lighting | 837 | Thygesen, L. G., and Hoffmeyer, P. (2005). Image analysis for the quantification of dislocations in hemp fibres. *Ind. Crops Prod.* 21, 173–184. doi:10.1016/j.indcrop.2004.03.001. |  |
| Cannabis lighting | 838 | Tighe, P. A. (2016). UNDERBANKED: COOPERATIVE BANKING AS A POTENTIAL SOLUTION TO THE MARIJUANA-BANKING PROBLEM. *Mich. Law Rev.* 114, 803–832. |  |
| Cannabis lighting | 839 | Toftdahl, N. G., Nordentoft, M., and Hjorthoj, C. (2016). The Effect of Changes in Cannabis Exposure on Psychotic Symptoms in Patients With Comorbid Cannabis Use Disorder. *J. Dual Diagn.* 12, 129–136. doi:10.1080/15504263.2016.1176426. |  |
| Cannabis lighting | 840 | Treister-Goltzman, Y., Freud, T., Press, Y., and Peleg, R. (2019). Trends in Publications on Medical Cannabis from the Year 2000. *Popul. Health Manag.* 22, 362–368. doi:10.1089/pop.2018.0113. |  |
| Cannabis lighting | 841 | Trezza, V., and Vanderschuren, L. J. M. J. (2008). Bidirectional cannabinoid modulation of social behavior in adolescent rats. *Psychopharmacology (Berl).* 197, 217–227. doi:10.1007/s00213-007-1025-3. |  |
| Cannabis lighting | 842 | Trofin, I. G., Dabija, G., Vaireanu, D.-I., and Filipescu, L. (2012a). The Influence of Long-term Storage Conditions on the Stability of Cannabinoids derived from Cannabis Resin. *Rev. Chim.* 63, 422–427. |  |
| Cannabis lighting | 843 | Trofin, I. G., Dabija, G., Vaireanu, D. I., and Filipescu, L. (2012b). Long-term Storage and Cannabis Oil Stability. *Rev. Chim.* 63, 293–297. |  |
| Cannabis lighting | 844 | Trofin, I. G., Vlad, C. C., Dabija, G., and Filipescu, L. (2011). Influence of Storage Conditions on the Chemical Potency of Herbal Cannabis. *Rev. Chim.* 62, 639–645. |  |
| Cannabis lighting | 845 | Tsujikawa, K., Saiki, F., Yamamuro, T., Iwata, Y. T., Abe, R., Ohashi, H., et al. (2016). Development of a novel immunoassay for herbal cannabis using a new fluorescent antibody probe, ``Ultra Quenchbody{’’}. *FORENSIC Sci. Int.* 266, 541–548. doi:10.1016/j.forsciint.2016.07.022. |  |
| Cannabis lighting | 846 | Turna, J., Patterson, B., and Van Ameringen, M. (2017). Is cannabis treatment for anxiety, mood, and related disorders ready for prime time? *Depress. Anxiety* 34, 1006–1017. doi:10.1002/da.22664. |  |
| Cannabis lighting | 847 | Valim Brigante, T. A., Abe, F. R., Zuardi, A. W., Cecilio Hallak, J. E., Crippa, J. A. S., and de Oliveira, D. P. (2018). Cannabidiol did not induce teratogenicity or neurotoxicity in exposed zebrafish embryos. *Chem. Biol. Interact.* 291, 81–86. doi:10.1016/j.cbi.2018.06.008. |  |
| Cannabis lighting | 848 | Valjent, E., Mitchell, J. M., Besson, M. J., Caboche, J., and Maldonado, R. (2002). Behavioural and biochemical evidence for interactions between Delta 9-tetrahydrocannabinol and nicotine. *Br. J. Pharmacol.* 135, 564–578. doi:10.1038/sj.bjp.0704479. |  |
| Cannabis lighting | 849 | van Heugten-Van der Kloet, D., Giesbrecht, T., van Wel, J., Bosker, W. M., Kuypers, K. P. C., Theunissen, E. L., et al. (2015). MDMA, cannabis, and cocaine produce acute dissociative symptoms. *PSYCHIATRY Res.* 228, 907–912. doi:10.1016/j.psychres.2015.04.028. |  |
| Cannabis lighting | 850 | Vanderbilt, V. C., and Daughtry, C. S. T. (2012). MUELLER MATRIX OF A DICOT LEAF. in *POLARIZATION: MEASUREMENT, ANALYSIS, AND REMOTE SENSING X* Proceedings of SPIE., ed. Chenault, DB and Goldstein, DH doi:10.1117/12.919480. |  |
| Cannabis lighting | 851 | VANDERWERF, H. M. G., BROUWER, K., WIJLHUIZEN, M., and WITHAGEN, J. C. M. (1995a). THE EFFECT OF TEMPERATURE ON LEAF APPEARANCE AND CANOPY ESTABLISHMENT IN FIBER HEMP (CANNABIS-SATIVA L). *Ann. Appl. Biol.* 126, 551–561. |  |
| Cannabis lighting | 852 | VanderWerf, H. M. G., Mathijssen, E., and Haverkort, A. J. (1996). The potential of hemp (Cannabis sativa L) for sustainable fibre production: A crop physiological appraisal. *Ann. Appl. Biol.* 129, 109–123. |  |
| Cannabis lighting | 853 | VANDERWERF, H. M. G., and VANDENBERG, W. (1995). NITROGEN-FERTILIZATION AND SEX EXPRESSION AFFECT SIZE VARIABILITY OF FIBER HEMP (CANNABIS-SATIVA L). *Oecologia* 103, 462–470. doi:10.1007/BF00328684. |  |
| Cannabis lighting | 854 | VANDERWERF, H. M. G., VANGEEL, W. C. A., VANGILS, L. J. C., and HAVERKORT, A. J. (1995b). NITROGEN-FERTILIZATION AND ROW WIDTH AFFECT SELF-THINNING AND PRODUCTIVITY OF FIBER HEMP (CANNABIS-SATIVA L). *F. Crop. Res.* 42, 27–37. doi:10.1016/0378-4290(95)00017-K. |  |
| Cannabis lighting | 855 | Vanhove, W., Van Damme, P., and Meert, N. (2011). Factors determining yield and quality of illicit indoor cannabis (Cannabis spp.) production. doi:10.1016/j.forsciint.2011.06.006. |  |
| Cannabis lighting | 856 | Varghese, D., Wray, N. R., Scott, J. G., Williams, G. M., Najman, J. M., and McGrath, J. J. (2013). The heritability of delusional-like experiences. *ACTA Psychiatr. Scand.* 127, 48–52. doi:10.1111/j.1600-0447.2012.01905.x. |  |
| Cannabis lighting | 857 | Vassos, E., Sham, P., Kempton, M., Trotta, A., Stilo, S. A., Gayer-Anderson, C., et al. (2020). The Maudsley environmental risk score for psychosis. *Psychol. Med.* 50, 2213–2220. doi:10.1017/S0033291719002319. |  |
| Cannabis lighting | 858 | Verges, A., Ellingson, J. M., Schroder, S. A., Slutske, W. S., and Sher, K. J. (2018). Intensity of Daily Drinking and Its Relation to Alcohol Use Disorders. *Alcohol. Exp. Res.* 42, 1674–1683. doi:10.1111/acer.13812. |  |
| Cannabis lighting | 859 | Vidot, D. C., Bispo, J. B., Hlaing, W. M., Prado, G., and Messiah, S. E. (2017). Moderate and vigorous physical activity patterns among marijuana users: Results from the 2007-2014 National Health and Nutrition Examination Surveys. *Drug Alcohol Depend.* 178, 43–48. doi:10.1016/j.drugalcdep.2017.05.004. |  |
| Cannabis lighting | 860 | Vitiello, M., and Deck, R. (2018). Legalizing Marijuana: A View from Among the Weeds. *Hastings Law J.* 69, 961–984. |  |
| Cannabis lighting | 861 | Vulfsons, S., Minerbi, A., and Sahar, T. (2020). Cannabis and Pain Treatment-A Review of the Clinical Utility and a Practical Approach in Light of Uncertainty. *RAMBAM MAIMONIDES Med. J.* 11. doi:10.5041/RMMJ.10385. |  |
| Cannabis lighting | 862 | Walsh, J. M. (2008). New technology and new initiatives in US workplace testing. *FORENSIC Sci. Int.* 174, 120–124. doi:10.1016/j.forsciint.2007.03.011. |  |
| Cannabis lighting | 863 | Wang, C.-T., Wiedinmyer, C., Ashworth, K., Harley, P. C., Ortega, J., and Vizuete, W. (2019). Leaf enclosure measurements for determining volatile organic compound emission capacity from Cannabis spp. *Atmos. Environ.* 199, 80–87. doi:10.1016/j.atmosenv.2018.10.049. |  |
| Cannabis lighting | 864 | Wang, R., He, L.-S., Xia, B., Tong, J.-F., Li, N., and Peng, F. (2009). A MICROPROPAGATION SYSTEM FOR CLONING OF HEMP (CANNABIS SATIVA L.) BY SHOOT TIP CULTURE. *PAKISTAN J. Bot.* 41, 603–608. |  |
| Cannabis lighting | 865 | Wang, X., Dow-Edwards, D., Anderson, V., Minkoff, H., and Hurd, Y. L. (2006). Discrete opioid gene expression impairment in the human fetal brain associated with maternal marijuana use. *Pharmacogenomics J.* 6, 255–264. doi:10.1038/sj.tpj.6500375. |  |
| Cannabis lighting | 866 | Wang, Y.-H., Avula, B., ElSohly, M. A., Radwan, M. M., Wang, M., Wanas, A. S., et al. (2018). Quantitative Determination of (9) -THC, CBG, CBD, Their Acid Precursors and Five Other Neutral Cannabinoids by UHPLC-UV-MS. *Planta Med.* 84, 260–266. doi:10.1055/s-0043-124873. |  |
| Cannabis lighting | 867 | Wankhade, V. K., and Chikhalkar, B. G. (2018). Body packing and intra-vaginal body pushing of cocaine: A case report. *Leg. Med.* 31, 10–13. doi:10.1016/j.legalmed.2017.12.004. |  |
| Cannabis lighting | 868 | Wanner, N. M., Colwell, M. L., and Faulk, C. (2019). The epigenetic legacy of illicit drugs: developmental exposures and late-life phenotypes. *Environ. EPIGENETICS* 5. doi:10.1093/eep/dvz022. |  |
| Cannabis lighting | 869 | Wardell, J. D., Shuper, P. A., and Hendershot, C. S. (2018). A longitudinal investigation of the association between cannabis use and alcohol use among people living with HIV. *Drug Alcohol Depend.* 193, 7–13. doi:10.1016/j.drugalcdep.2018.08.026. |  |
| Cannabis lighting | 870 | Wardle, M. C., Marcus, B. A., and de Wit, H. (2015). A Preliminary Investigation of Individual Differences in Subjective Responses to D-Amphetamine, Alcohol, and Delta-9-Tetrahydrocannabinol Using a WithinSubjects Randomized Trial. *PLoS One* 10. doi:10.1371/journal.pone.0140501. |  |
| Cannabis lighting | 871 | Wemm, S. E., and Sinha, R. (2019). Drug-induced stress responses and addiction risk and relapse. *Neurobiol. Stress* 10. doi:10.1016/j.ynstr.2019.100148. |  |
| Cannabis lighting | 872 | Wen, J., Meng, F., Ying, T., and Belhassen, Y. (2020). A study of segmentation of cannabis-oriented tourists from China based on motivation. *Curr. ISSUES Tour.* 23, 36–51. doi:10.1080/13683500.2019.1584608. |  |
| Cannabis lighting | 873 | Wetherell, M. A., and Montgomery, C. (2014). Basal functioning of the hypothalamic-pituitary-adrenal (HPA) axis and psychological distress in recreational ecstasy polydrug users. *Psychopharmacology (Berl).* 231, 1365–1375. doi:10.1007/s00213-013-3325-0. |  |
| Cannabis lighting | 874 | Whyte, L. S., Ryberg, E., Sims, N. A., Ridge, S. A., Mackie, K., Greasley, P. J., et al. (2009). The putative cannabinoid receptor GPR55 affects osteoclast function in vitro and bone mass in vivo. *Proc. Natl. Acad. Sci. U. S. A.* 106, 16511–16516. doi:10.1073/pnas.0902743106. |  |
| Cannabis lighting | 875 | Wiecko, F. M., and Thompson, W. E. (2014). Growin’ Grass: Paradise by the Sodium Light. *DEVIANT Behav.* 35, 332–345. doi:10.1080/01639625.2013.848122. |  |
| Cannabis lighting | 876 | Wiedermann, W., Niggli, J., and Frick, U. (2014). The Lemming-effect: harm perception of psychotropic substances among music festival visitors. *Heal. RISK Soc.* 16, 323–338. doi:10.1080/13698575.2014.930817. |  |
| Cannabis lighting | 877 | Wilcox, H. C., and Anthony, J. C. (2004). The development of suicide ideation and attempts: an epiderniologic study of first graders followed into young adulthood. *Drug Alcohol Depend.* 76, S53–S67. doi:10.1016/j.drugalcdep.2004.08.007. |  |
| Cannabis lighting | 878 | Wilkerson, J. L., Schulze, D. R., and Mcmahon, L. R. (2019). Tolerance and dependence to Delta(9)-tetrahydrocannabinol in rhesus monkeys: Activity assessments. *PLoS One* 14. doi:10.1371/journal.pone.0209947. |  |
| Cannabis lighting | 879 | Wilkinson, S. T., Yarnell, S., Radhakrishnan, R., Ball, S. A., and D’Souza, D. C. (2016). “Marijuana Legalization: Impact on Physicians and Public Health,” in *ANNUAL REVIEW OF MEDICINE, VOL 67* Annual Review of Medicine., ed. Caskey, CT, 453–466. doi:10.1146/annurev-med-050214-013454. |  |
| Cannabis lighting | 880 | Williams, J., and Skeels, C. L. (2006). The impact of cannabis use on health. *ECONOMIST-NETHERLANDS* 154, 517–546. doi:10.1007/s10645-006-9028-8. |  |
| Cannabis lighting | 881 | Wilsey, B., Marcotte, T., Tsodikov, A., Millman, J., Bentley, H., Gouaux, B., et al. (2008). A randomized, placebo-controlled, crossover trial of cannabis cigarettes in neuropathic pain. *J. PAIN* 9, 506–521. doi:10.1016/j.jpain.2007.12.010. |  |
| Cannabis lighting | 882 | Wilson, W., Mathew, R., Turkington, T., Hawk, T., Coleman, R. E., and Provenzale, J. (2000). Brain morphological changes and early marijuana use: A magnetic resonance and positron emission tomography study. *J. Addict. Dis.* 19, 1–22. doi:10.1300/J069v19n01_01. |  |
| Cannabis lighting | 883 | Wiltshire, P. E. J., Hawksworth, D. L., and Edwards, K. J. (2015). Light microscopy can reveal the consumption of a mixture of psychotropic plant and fungal material in suspicious death. *J. Forensic Leg. Med.* 34, 73–80. doi:10.1016/j.jflm.2015.05.010. |  |
| Cannabis lighting | 884 | Wizenberg, S. B., Weis, A. E., and Campbell, L. G. (2020). Comparing methods for controlled capture and quantification of pollen inCannabis sativa. *Appl. Plant Sci.* 8. doi:10.1002/aps3.11389. |  |
| Cannabis lighting | 885 | Wurst, F. M., Wiesbeck, G. A., Metzger, J. W., Weinmann, W., Graf, M., and State, W. H. O. I. S. B. (2004). On sensitivity, specificity, and the influence of various parameters on ethyl glucuronide levels in urine - Results from the WHO/ISBRA study. *Alcohol. Exp. Res.* 28, 1220–1228. doi:10.1097/01.ALC.0000134230.21414.11. |  |
| Cannabis lighting | 886 | Xia, C., Hong, L., Yang, Y., Yanping, X., Xing, H., and Gang, D. (2019). Protein Changes in Response to Lead Stress of Lead-Tolerant and Lead-Sensitive Industrial Hemp Using SWATH Technology. *Genes (Basel).* 10. doi:10.3390/genes10050396. |  |
| Cannabis lighting | 887 | Yazulla, S. (2008). Endocannabinoids in the retina: From marijuana to neuroprotection. *Prog. Retin. Eye Res.* 27, 501–526. doi:10.1016/j.preteyeres.2008.07.002. |  |
| Cannabis lighting | 888 | Young, P., Vadala, S., Finn, B. C., Pankl, S., Montes Ongania, A., and Bruetman, J. E. (2019). Marijuana-associated peripheral arteriopathy. *Med. AIRES* 79, 144–146. |  |
| Cannabis lighting | 889 | Yuecel, M., Zalesky, A., Takagi, M. J., Bora, E., Fornito, A., Ditchfield, M., et al. (2010). White-matter abnormalities in adolescents with long-term inhalant and cannabis use: a diffusion magnetic resonance imaging study. *J. PSYCHIATRY Neurosci.* 35, 409–412. doi:10.1503/jpn.090177. |  |
| Cannabis lighting | 890 | Zamengo, L., Bettin, C., Badocco, D., Di Marco, V., Miolo, G., and Frison, G. (2019). The role of time and storage conditions on the composition of hashish and marijuana samples: A four-year study. *FORENSIC Sci. Int.* 298, 131–137. doi:10.1016/j.forsciint.2019.02.058. |  |
| Cannabis lighting | 891 | Zarhin, D. (2020). The trajectory of ``medical cannabis{’’} in Israel: Driving medicalization in different directions. *Int. J. DRUG POLICY* 82. doi:10.1016/j.drugpo.2020.102809. |  |
| Cannabis lighting | 892 | Zarhin, D., Negev, M., Vulfsons, S., and Sznitman, S. R. (2018). Rhetorical and regulatory boundary-work: The case of medical cannabis policy-making in Israel. *Soc. Sci. Med.* 217, 1–9. doi:10.1016/j.socscimed.2018.09.047. |  |
| Cannabis lighting | 893 | Zarhin, D., Negev, M., Vulfsons, S., and Sznitman, S. R. (2020). ``Medical Cannabis{’’} as a Contested Medicine: Fighting Over Epistemology and Morality. *Sci. Technol. \& Hum. VALUES* 45, 488–514. doi:10.1177/0162243919862866. |  |
| Cannabis lighting | 894 | Zerva, K., and Nijkamp, P. (2016). Tour guides as information filters in urban heterotopias: Evidence from the Amsterdam Red Light District. *Tour. Manag. Perspect.* 18, 42–50. doi:10.1016/j.tmp.2015.12.020. |  |
| Cannabis lighting | 895 | Zivovinovic, S., Alder, R., Allenspach, M. D., and Steuer, C. (2018). Determination of cannabinoids in Cannabis sativa L. samples for recreational, medical, and forensic purposes by reversed-phase liquid chromatography-ultraviolet detection. *J. Anal. Sci. Technol.* 9. doi:10.1186/s40543-018-0159-8. |  |
| Cannabis lighting | 896 | Zobor, D., Strasser, T., Zobor, G., Schober, F., Messias, A., Strauss, O., et al. (2015). Ophthalmological assessment of cannabis-induced persisting perception disorder: Is there a direct retinal effect? *Doc. Ophthalmol.* 130, 121–130. doi:10.1007/s10633-015-9481-2. |  |
| Cannabis lighting | 897 | Zohsel, K., Baldus, C., Schmidt, M. H., Esser, G., Banaschewski, T., Thomasius, R., et al. (2016). Predicting later problematic cannabis use from psychopathological symptoms during childhood and adolescence: Results of a 25-year longitudinal study. *Drug Alcohol Depend.* 163, 251–255. doi:10.1016/j.drugalcdep.2016.04.012. |  |
| Cannabis day length | 898 | AbdelFattah, K. R., Edwards, C. R., Cripps, M. W., Minshall, C. T., Phelan, H. A., Minei, J. P., et al. (2017). Cannabis Use Has Negligible Effects Following Severe Traumatic Injury. *J. TRAUMA Nurs.* 24, 141–145. doi:10.1097/JTN.0000000000000277. |  |
| Cannabis day length | 899 | Aggarwal, S. K., Carter, G. T., Zumbrunnen, C., Morrill, R., Sullivan, M., and Mayer, J. D. (2013). From 32 Ounces to Zero: A Medical Geographic Study of Dispensing a Cultivated Batch of ``Plum{’’} Cannabis Flowers to Medical Marijuana Patients in Washington State. *J. Psychoactive Drugs* 45, 141–155. doi:10.1080/02791072.2013.785835. |  |
| Cannabis day length | 900 | Ahmad, N., Robert, C. A., Jampa, A., Ashraf, S., and Patel, R. S. (2019). Antepartum Drug Dependence and Pregnancy- or Birth-related Complications: A Cross-sectional Study of 19 Million Inpatients. *CUREUS* 11. doi:10.7759/cureus.6117. |  |
| Cannabis day length | 901 | Al Madini, A. M., Sassine, Y. N., El-Ganainy, S. M., Hourani, W., and El Sebaaly, Z. (2019). COMPARATIVE STUDY ON PHENOLOGY, YIELD AND QUALITY OF IRANIAN SAFFRON CULTIVATED IN LEBANON AND IRAN. *FRESENIUS Environ. Bull.* 28, 9655–9660. |  |
| Cannabis day length | 902 | Alexandre, A. T., Vale, A., and Gomes, T. (2019). DIFFUSE ALVEOLAR HEMORRHAGE: HOW RELEVANT IS ETIOLOGY? *SARCOIDOSIS Vasc. Diffus. LUNG Dis.* 36, 47–52. |  |
| Cannabis day length | 903 | Allsop, D. J., Bartlett, D. J., Johnston, J., Helliwell, D., Winstock, A., McGregor, I. S., et al. (2015). The Effects of Lithium Carbonate Supplemented with Nitrazepam on Sleep Disturbance during Cannabis Abstinence. *J. Clin. SLEEP Med.* 11, 1153–1162. doi:10.5664/jcsm.5090. |  |
| Cannabis day length | 904 | Amaducci, S., Colauzzi, M., Bellocchi, G., and Venturi, G. (2008). Modelling post-emergent hemp phenology (Cannabis sativa L.): Theory and evaluation. *Eur. J. Agron.* 28, 90–102. doi:10.1016/j.eja.2007.05.006. |  |
| Cannabis day length | 905 | Anderson, S. R., Wimalawansa, S. M., Markov, N. P., and Fox, J. P. (2020). Cannabis Abuse or Dependence and Postoperative Outcomes After Appendectomy and Cholecystectomy. *J. Surg. Res.* 255, 233–239. doi:10.1016/j.jss.2020.05.009. |  |
| Cannabis day length | 906 | Benevenuto, S. G., Domenico, M. D., Martins, M. A. G., Costa, N. S., de Souza, A. R. L., Costa, J. L., et al. (2017). Recreational use of marijuana during pregnancy and negative gestational and fetal outcomes: An experimental study in mice. *Toxicology* 376, 94–101. doi:10.1016/j.tox.2016.05.020. |  |
| Cannabis day length | 907 | Breit, K. R., Rodriguez, C. G., Lei, A., and Thomas, J. D. (2020). Combined vapor exposure to THC and alcohol in pregnant rats: Maternal outcomes and pharmacokinetic effects. *Neurotoxicol. Teratol.* 82. doi:10.1016/j.ntt.2020.106930. |  |
| Cannabis day length | 908 | Campbell, L. G., Naraine, S. G. U., and Dusfresne, J. (2019). Phenotypic plasticity influences the success of clonal propagation in industrial pharmaceutical Cannabis sativa. *PLoS One* 14. doi:10.1371/journal.pone.0213434. |  |
| Cannabis day length | 909 | Charilaou, P., Agnihotri, K., Garcia, P., Badheka, A., Frenia, D., and Yegneswaran, B. (2017). Trends of Cannabis Use Disorder in the Inpatient: 2002 to 2011. *Am. J. Med.* 130, 678-U258. doi:10.1016/j.amjmed.2016.12.035. |  |
| Cannabis day length | 910 | Cigasova, J., Stevulova, N., Terpakova, E., Junak, J., and Sicakova, A. (2013). INVESTIGATION OF COMPOSITES PROPERTIES PREPARED BY USING HEMP HURDS AND ALTERNATIVE BINDERS. in *PROCEEDINGS OF THE 13TH INTERNATIONAL CONFERENCE ON ENVIRONMENTAL SCIENCE AND TECHNOLOGY* Proceedings of the International Conference on Environmental Science and Technology., ed. Lekkas, TD. |  |
| Cannabis day length | 911 | Colizzi, M., Burnett, N., Costa, R., De Agostini, M., Griffin, J., and Bhattacharyya, S. (2018). Longitudinal assessment of the effect of cannabis use on hospital readmission rates in early psychosis: A 6-year follow-up in an inpatient cohort. *PSYCHIATRY Res.* 268, 381–387. doi:10.1016/j.psychres.2018.08.005. |  |
| Cannabis day length | 912 | Cosentino, S. L., Testa, G., Scordia, D., and Copani, V. (2012). Sowing time and prediction of flowering of different hemp (Cannabis sativa L.) genotypes in southern Europe. *Ind. Crops Prod.* 37, 20–33. |  |
| Cannabis day length | 913 | Coughlan, D., Milner, M., Clarke, T., Lambert, I., McDermott, C., McNally, M., et al. (1999). Neonatal abstinence syndrome. *Ir. Med. J.* 92, 232+. |  |
| Cannabis day length | 914 | da Costa e Silva, L. I., da Silva Gomes, F. M., Valente, M. H., Escobar, A. de U., Maria Brentani, A. V., and Grisi, S. J. F. E. (2015). The Intergenerational Effects on Birth Weight and Its Relations to Maternal Conditions, Sao Paulo, Brazil. *Biomed Res. Int.* doi:10.1155/2015/615034. |  |
| Cannabis day length | 915 | Dakour-Aridi, H., Arora, M., Nejim, B., Locham, S., and Malas, M. B. (2019). Association between Drug Use and In-hospital Outcomes after Infrainguinal Bypass for Peripheral Arterial Occlusive Disease. *Ann. Vasc. Surg.* 58, 122+. doi:10.1016/j.avsg.2018.12.070. |  |
| Cannabis day length | 916 | DEMEIJER, E. P. M., and KEIZER, L. C. P. (1994). VARIATION OF CANNABIS FOR PHENOLOGICAL DEVELOPMENT AND STEM ELONGATION IN RELATION TO STEM PRODUCTION. *F. Crop. Res.* 38, 37–46. doi:10.1016/0378-4290(94)90030-2. |  |
| Cannabis day length | 917 | Deng, H., Desai V, P., Mohite, S., Okusaga, O. O., Zhang, X. Y., Nielsen, D. A., et al. (2019). Hospital Stay in Synthetic Cannabinoid Users With Bipolar Disorder, Schizophrenia, or Other Psychotic Disorders Compared With Cannabis Users. *J. Stud. Alcohol Drugs* 80, 230–235. |  |
| Cannabis day length | 918 | Desai, P., Mbachi, C., Vohra, I., Salazar, M., Mathew, M., Randhawa, T., et al. (2020). Association Between Cannabis Use and Healthcare Utilization in Patients With Irritable Bowel Syndrome: A Retrospective Cohort Study. *CUREUS* 12. doi:10.7759/cureus.8008. |  |
| Cannabis day length | 919 | Desai, R., Patel, U., Goyal, H., Rimu, A. H., Zalavadia, D., Bansal, P., et al. (2019). In-hospital outcomes of inflammatory bowel disease in cannabis users: a nationwide propensity-matched analysis in the United States. *Ann. Transl. Med.* 7. doi:10.21037/atm.2019.04.63. |  |
| Cannabis day length | 920 | Dominguez-Vega, G., Pera, M., Ramon, J. M., Puig, S., Membrilla, E., Sancho, J., et al. (2013). A comparison of laparoscopic versus open repair for the surgical treatment of perforated peptic ulcers. *Cir. Esp.* 91, 372–377. doi:10.1016/j.ciresp.2012.10.016. |  |
| Cannabis day length | 921 | Dominguez, M.-G., Fisher, H. L., Major, B., Chisholm, B., Rahaman, N., Joyce, J., et al. (2013). Duration of untreated psychosis in adolescents: Ethnic differences and clinical profiles. *Schizophr. Res.* 150, 526–532. doi:10.1016/j.schres.2013.08.018. |  |
| Cannabis day length | 922 | Etyemez, S., Currie, T. T., Hamilton, J. E., Weaver, M. F., Findley, J. C., Soares, J., et al. (2020). Cannabis use: A co-existing condition in first-episode bipolar mania patients. *J. Affect. Disord.* 263, 289–291. doi:10.1016/j.jad.2019.11.097. |  |
| Cannabis day length | 923 | Feinstein, A., and Holloway, F. (2002). Evaluating the use of a psychiatric intensive care unit: Is ethnicity a risk factor for admission? *Int. J. Soc. Psychiatry* 48, 38–46. doi:10.1177/002076402128783073. |  |
| Cannabis day length | 924 | Fekete, S., Sadat-Noorbakhsh, V., Schelling, C., Molnar, I., Guillarme, D., Rudaz, S., et al. (2018). Implementation of a generic liquid chromatographic method development workflow: Application to the analysis of phytocannabinoids and Cannabis sativa extracts. *J. Pharm. Biomed. Anal.* 155, 116–124. doi:10.1016/j.jpba.2018.03.059. |  |
| Cannabis day length | 925 | Fischer, G., Johnson, R. E., Eder, H., Jagsch, R., Peternell, A., Weninger, M., et al. (2000). Treatment of opioid-dependent pregnant women with buprenorphine. *ADDICTION* 95, 239–244. doi:10.1046/j.1360-0443.2000.95223910.x. |  |
| Cannabis day length | 926 | Flvig, J. C., Vaaler, A. E., and Morken, G. (2009). Substance use at admission to an acute psychiatric department. *Nord. J. Psychiatry* 63, 113–119. doi:10.1080/08039480802294787. |  |
| Cannabis day length | 927 | Gosens, L. C. F., Otten, R., Didden, R., and Poelen, E. A. P. (2020). Evaluating a personalized treatment for substance use disorder in people with mild intellectual disability or borderline intellectual functioning: A study protocol of a multiple baseline across individuals design. *Contemp. Clin. TRIALS Commun.* 19. doi:10.1016/j.conctc.2020.100616. |  |
| Cannabis day length | 928 | Grzeskowiale, L. E., Grieger, J. A., Andraweera, P., Knight, E., Leemaqz, S., Poston, L., et al. The deleterious effects of cannabis during pregnancy on neonatal outcomes. *Med. J. Aust.* doi:10.5694/mja2.50624. |  |
| Cannabis day length | 929 | Hall, J., Bhattarai, S. P., and Midmore, D. J. (2012). Review of Flowering Control in Industrial Hemp. *J. Nat. FIBERS* 9, 23–36. doi:10.1080/15440478.2012.651848. |  |
| Cannabis day length | 930 | Hall, J., Bhattarai, S. P., and Midmore, D. J. (2013). The Effects of Different Sowing Times on Maturity Rates, Biomass, and Plant Growth of Industrial Fiber Hemp. *J. Nat. FIBERS* 10, 40–50. doi:10.1080/15440478.2012.756639. |  |
| Cannabis day length | 931 | Hall, J., Bhattarai, S. P., and Midmore, D. J. (2014). The Effects of Photoperiod on Phenological Development and Yields of Industrial Hemp. *J. Nat. FIBERS* 11, 87–106. doi:10.1080/15440478.2013.846840. |  |
| Cannabis day length | 932 | Heizer, J. W., Borgelt, L. M., Bashqoy, F., Wang, G. S., and Reiter, P. D. (2018). Marijuana Misadventures in Children: Exploration of a Dose-Response Relationship and Summary of Clinical Effects and Outcomes. *Pediatr. Emerg. Care* 34, 457–462. doi:10.1097/PEC.0000000000000770. |  |
| Cannabis day length | 933 | Hickernell, T. R., Lakra, A., Berg, A., Cooper, H. J., Geller, J. A., and Shah, R. P. (2018). Should Cannabinoids Be Added to Multimodal Pain Regimens After Total Hip and Knee Arthroplasty? *J. Arthroplasty* 33, 3637–3641. doi:10.1016/j.arth.2018.07.027. |  |
| Cannabis day length | 934 | Himes, S. K., Stroud, L. R., Scheidweiler, K. B., Niaura, R. S., and Huestis, M. A. (2013). Prenatal Tobacco Exposure, Biomarkers for Tobacco in Meconium, and Neonatal Growth Outcomes. *J. Pediatr.* 162, 970–975. doi:10.1016/j.jpeds.2012.10.045. |  |
| Cannabis day length | 935 | Ioannidis, K., Dadiotis, E., Mitsis, V., Melliou, E., and Magiatis, P. (2020). Biotechnological Approaches on Two High CBD and CBG Cannabis sativa L. (Cannabaceae) Varieties: In Vitro Regeneration and Phytochemical Consistency Evaluation of Micropropagated Plants Using Quantitative H-1-NMR. *MOLECULES* 25. doi:10.3390/molecules25245928. |  |
| Cannabis day length | 936 | Jaladi, P. R., Patel, V., Rajan, S. K., Rashid, W., Madireddy, S., Ajibawo, T., et al. (2019). Arrhythmia-related Hospitalization and Comorbid Cannabis Use Disorder: Trend Analysis in US Hospitals (2010-2014). *CUREUS* 11. doi:10.7759/cureus.5607. |  |
| Cannabis day length | 937 | Jankauskiene, Z., and Gruzdeviene, E. (2010). Evaluation of Cannabis sativa cultivars in Lithuania. *ZEMDIRBYSTE-AGRICULTURE* 97, 87–96. |  |
| Cannabis day length | 938 | Jehle Jr., C. C., Nazir, N., and Bhavsar, D. (2015). The Rapidly Increasing Trend of Cannabis Use in Burn Injury. *J. Burn CARE Res.* 36, E12–E17. doi:10.1097/BCR.0000000000000192. |  |
| Cannabis day length | 939 | Jonas, B., Tensil, M.-D., Tossmann, P., and Strueber, E. (2018). Effects of Treatment Length and Chat-Based Counseling in a Web-Based Intervention for Cannabis Users: Randomized Factorial Trial. *J. Med. INTERNET Res.* 20. doi:10.2196/jmir.9579. |  |
| Cannabis day length | 940 | Kadar, I., and Szucs, S. T. (2003). Effect of mineral fertilisation on the yield of fibre hemp (Cannabis sativa L.). *NOVENYTERMELES* 52, 217–228. |  |
| Cannabis day length | 941 | Kalousek, P., Schreiber, P., Vyhnanek, T., Trojan, V., Adamcova, D., and Vaverkova, M. D. (2020). Effect of Landfill Leachate on the Growth Parameters in Two Selected Varieties of Fiber Hemp. *Int. J. Environ. Res.* 14, 155–163. doi:10.1007/s41742-020-00249-2. |  |
| Cannabis day length | 942 | Khan, F., Asif, M., Khan, A., Tariq, M., Ansari, T., Shariq, M., et al. (2019). Evaluation of the nematicidal potential of some botanicals against root-knot nematode, Meloidogyne incognita infected carrot: In vitro and greenhouse study. *Curr. PLANT Biol.* 20. doi:10.1016/j.cpb.2019.100115. |  |
| Cannabis day length | 943 | Klein, J., Karaskov, T., and Koren, G. (2000). Clinical applications of hair testing for drugs of abuse - the Canadian experience. *FORENSIC Sci. Int.* 107, 281–288. doi:10.1016/S0379-0738(99)00171-1. |  |
| Cannabis day length | 944 | Krzyzanowski, D. J., and Purdon, S. E. (2020). Duration of Abstinence From Cannabis Is Positively Associated With Verbal Learning Performance: A Systematic Review and Meta-Analysis. *Neuropsychology* 34, 359–372. doi:10.1037/neu0000615. |  |
| Cannabis day length | 945 | Lai, H. M. X., and Sitharthan, T. (2012). Exploration of the Comorbidity of Cannabis Use Disorders and Mental Health Disorders among Inpatients Presenting to All Hospitals in New South Wales, Australia. *Am. J. Drug Alcohol Abuse* 38, 567–574. doi:10.3109/00952990.2012.694523. |  |
| Cannabis day length | 946 | Lammert, S., Harrison, K., Tosun, N., and Allen, S. (2018). Menstrual Cycle in Women Who Co-use Marijuana and Tobacco. *J. Addict. Med.* 12, 207–211. doi:10.1097/ADM.0000000000000387. |  |
| Cannabis day length | 947 | Ledgerwood, D. M., Lister, J. J., LaLiberte, B., Lundahl, L. H., and Greenwald, M. K. (2019). Injection opioid use as a predictor of treatment outcomes among methadone-maintained opioid-dependent patients. *Addict. Behav.* 90, 191–195. doi:10.1016/j.addbeh.2018.10.046. |  |
| Cannabis day length | 948 | Lekoubou, A., Fox, J., Bishu, K. G., and Ovbiagele, B. (2020). Trends in documented cannabis use disorder among hospitalized adult epilepsy patients in the United States. *EPILEPSY Res.* 163. doi:10.1016/j.eplepsyres.2020.106341. |  |
| Cannabis day length | 949 | Leskovan, J. J., Patel, P. D., Pederson, J., Moore, A., Afaneh, A., and Brown, L. R. (2020). The combined effects of alcohol and marijuana use prior to traumatic brain injury on mortality. *Ann. Med. Surg.* 60, 639–643. doi:10.1016/j.amsu.2020.11.059. |  |
| Cannabis day length | 950 | Madireddy, S., Patel, R. S., Ravat, V., Afibawo, T., Lal, A., Patel, J., et al. (2019). Burden of Comorbidities in Hospitalizations for Cannabis Use-associated Intractable Vomiting during Post-legalization Period. *CUREUS* 11. doi:10.7759/cureus.5502. |  |
| Cannabis day length | 951 | Massey, S. H., Mroczek, D. K., Reis, D., Miller, E. S., Jakubowski, J. A., Graham, E. K., et al. (2018). Additive drug-specific and sex-specific risks associated with co-use of marijuana and tobacco during pregnancy: Evidence from 3 recent developmental cohorts (2003-2015). *Neurotoxicol. Teratol.* 68, 97–106. doi:10.1016/j.ntt.2018.06.002. |  |
| Cannabis day length | 952 | McGarvey, P., Huang, J., McCoy, M., Orvis, J., Katsir, Y., Lotringer, N., et al. (2020). De novo assembly and annotation of transcriptomes from two cultivars of Cannabis sativa with diff erent cannabinoid profiles. *Gene* 762. doi:10.1016/j.gene.2020.145026. |  |
| Cannabis day length | 953 | Montgomery, C., Seddon, A. L., Fisk, J. E., Murphy, P. N., and Jansari, A. (2012). Cannabis-related deficits in real-world memory. *Hum. Psychopharmacol. Exp.* 27, 217–225. doi:10.1002/hup.1273. |  |
| Cannabis day length | 954 | Natale, B. V, Gustin, K. N., Lee, K., Holloway, A. C., Laviolette, S. R., Natale, D. R. C., et al. (2020). Delta 9-tetrahydrocannabinol exposure during rat pregnancy leads to symmetrical fetal growth restriction and labyrinth-specific vascular defects in the placenta. *Sci. Rep.* 10. doi:10.1038/s41598-019-57318-6. |  |
| Cannabis day length | 955 | Neeki, M. M., Dong, F., Liang, L., Toy, J., Carrico, B., Jabourian, N., et al. (2018). Evaluation of the effect of methamphetamine on traumatic injury complications and outcomes. *Addict. Sci. Clin. Pract.* 13. doi:10.1186/s13722-018-0112-6. |  |
| Cannabis day length | 956 | Onyeka, I. N., Beynon, C. M., Ronkainen, K., Tiihonen, J., Fohr, J., Kuikanmaki, O., et al. (2015). Hospitalization in a Cohort Seeking Treatment for Illicit Drug Use in Finland. *J. Subst. Abuse Treat.* 53, 64–70. doi:10.1016/j.jsat.2014.12.009. |  |
| Cannabis day length | 957 | Pamplona, F. A., da Silva, L. R., and Coan, A. C. (2018). Potential Clinical Benefits of CBD-Rich Cannabis Extracts Over Purified CBD in Treatment-Resistant Epilepsy: Observational Data Meta-analysis. *Front. Neurol.* 9. doi:10.3389/fneur.2018.00759. |  |
| Cannabis day length | 958 | Paruk, S., Ramlall, S., and Burns, J. K. (2009). Adolescent-onset psychosis: A 2-year retrospective study of adolescents admitted to a general psychiatric unit. *SOUTH AFRICAN J. PSYCHIATRY* 15, 86–92. |  |
| Cannabis day length | 959 | Pava, M. J., Makriyannis, A., and Lovinger, D. M. (2016). Endocannabinoid Signaling Regulates Sleep Stability. *PLoS One* 11. doi:10.1371/journal.pone.0152473. |  |
| Cannabis day length | 960 | Peters, E. N., Schauer, G. L., Rosenberry, Z. R., and Pickworth, W. B. (2016). Does marijuana ``blunt{’’} smoking contribute to nicotine exposure?: Preliminary product testing of nicotine content in wrappers of cigars commonly used for blunt smoking. *Drug Alcohol Depend.* 168, 119–122. doi:10.1016/j.drugalcdep.2016.09.007. |  |
| Cannabis day length | 961 | Potter, D. J., and Duncombe, P. (2012). The Effect of Electrical Lighting Power and Irradiance on Indoor-Grown Cannabis Potency and Yield. *J. Forensic Sci.* 57, 618–622. doi:10.1111/j.1556-4029.2011.02024.x. |  |
| Cannabis day length | 962 | Pudelko, K., Majchrzak, L., and Narozna, D. (2014). Allelopathic effect of fibre hemp (Cannabis sativa L.) on monocot and dicot plant species. *Ind. Crops Prod.* 56, 191–199. doi:10.1016/j.indcrop.2014.02.028. |  |
| Cannabis day length | 963 | Reilly, D., Didcott, P., Swift, W., and Hall, W. (1998). Long-term cannabis use: characteristics of users in an Australian rural area. *ADDICTION* 93, 837–846. doi:10.1046/j.1360-0443.1998.9368375.x. |  |
| Cannabis day length | 964 | Rendon, A., Livingston, M., Suzuki, S., Hill, W., and Walters, S. (2017). What’s the agreement between self-reported and biochemical verification of drug use? A look at permanent supportive housing residents. *Addict. Behav.* 70, 90–96. doi:10.1016/j.addbeh.2017.02.011. |  |
| Cannabis day length | 965 | Rumalla, K., Smith, K. A., Arnold, P. M., and Mittal, M. K. (2018). Subarachnoid Hemorrhage and Readmissions: National Rates, Causes, Risk Factors, and Outcomes in 16,001 Hospitalized Patients. *WORLD Neurosurg.* 110, E100–E111. doi:10.1016/j.wneu.2017.10.089. |  |
| Cannabis day length | 966 | Rylander, M., Winston, H. R., Medlin, H., Hull, M., and Nussbaum, A. (2018). The association of cannabis use on inpatient psychiatric hospital outcomes. *Am. J. Drug Alcohol Abuse* 44, 73–84. doi:10.1080/00952990.2017.1329313. |  |
| Cannabis day length | 967 | Sankari, H. S. (2000). Comparison of bast fibre yield and mechanical fibre properties of hemp (Cannabis sativa L.) cultivars. *Ind. Crops Prod.* 11, 73–84. doi:10.1016/S0926-6690(99)00038-2. |  |
| Cannabis day length | 968 | Sausserde, R., and Adamovics, A. (2013). IMPACT OF NITROGEN FERTILIZER RATES ON INDUSTRIAL HEMP GROWTH AND DEVELOPMENT. in *RESEARCH FOR RURAL DEVELOPMENT 2013, VOL 1* Research for Rural Development., ed. Treija, S and Skujeniece, S, 50–55. |  |
| Cannabis day length | 969 | Schmidt, L. M., Hesse, M., and Lykke, J. (2011). The impact of substance use disorders on the course of schizophrenia-A 15-year follow-up study Dual diagnosis over 15 years. *Schizophr. Res.* 130, 228–233. doi:10.1016/j.schres.2011.04.011. |  |
| Cannabis day length | 970 | Sera, B., Gajdova, I., Gavril, B., Hnatiuc, E., Sery, M., and Spatenka, P. (2012). Hemp (Cannabis sativa L.) seeds after plasma treatment. in *PROCEEDINGS OF THE 13TH INTERNATIONAL CONFERENCE ON OPTIMIZATION OF ELECTRICAL AND ELECTRONIC EQUIPMENT, VOLS 1-5* Proceedings of the International Conference on Optimization of Electrical and Electronic Equipment., ed. Clotea, LR and Cernat, M, 1371–1374. |  |
| Cannabis day length | 971 | Shalit, N., Barzilay, R., Shoval, G., Shlosberg, D., Mor, N., Zweigenhaft, N., et al. (2016). Characteristics of Synthetic Cannabinoid and Cannabis Users Admitted to a Psychiatric Hospital: A Comparative Study. *J. Clin. Psychiatry* 77, E989–E995. doi:10.4088/JCP.15m09938. |  |
| Cannabis day length | 972 | Shelef, K., and Diamond, G. M. (2008). Short form of the revised vanderbilt therapeutic alliance scale: Development, reliability, and validity. *Psychother. Res.* 18, 433–443. doi:10.1080/10503300701810801. |  |
| Cannabis day length | 973 | Siefried, K. J., Acheson, L. S., Lintzeris, N., and Ezard, N. (2020). Pharmacological Treatment of Methamphetamine/Amphetamine Dependence: A Systematic Review. *CNS Drugs* 34, 337–365. doi:10.1007/s40263-020-00711-x. |  |
| Cannabis day length | 974 | Singer, M., Azim, A., O’Keeffe, T., Khan, M., Jain, A., Kulvatunyou, N., et al. (2017). How does marijuana affect outcomes after trauma in ICU patients? A propensity-matched analysis. *J. Trauma Acute Care Surg.* 83, 846–849. doi:10.1097/TA.0000000000001672. |  |
| Cannabis day length | 975 | Spano, S., Macias, F., Snowden, B., and Vohra, R. (2013). Snakebite Survivors Club: Retrospective review of rattlesnake bites in Central California. *TOXICON* 69, 38–41. doi:10.1016/j.toxicon.2012.11.015. |  |
| Cannabis day length | 976 | Takanol, A., Miyamotol, Y., Kawakami, N., and Matsumoto, T. (2016). Web-Based Cognitive Behavioral Relapse Prevention Program With Tailored Feedback for People With Methamphetamine and Other Drug Use Problems: Development and Usability Study. *JMIR Ment. Heal.* 3. doi:10.2196/mental.4875. |  |
| Cannabis day length | 977 | Vadukapuram, R., Zahid, S., Lee, H. K., and Patel, R. S. (2020). Region-Wise Distribution of Schizophrenia With Cannabis Abuse and Medication Non-Compliance in the United States: A Nationwide Analysis of 51,975 Hospitalizations. *CUREUS* 12. doi:10.7759/cureus.7936. |  |
| Cannabis day length | 978 | von Mandach, U., Rabner, M. M., Wisser, J., and Huch, A. (1999). LSD and cannabis abuse in early pregnancy with good perinatal outcome: Case report and review of the literature. *Gynakol. Geburtshilfliche. Rundsch.* 39, 125–129. doi:10.1159/000022292. |  |
| Cannabis day length | 979 | Vrdoljak, A. L., Fuchs, N., Mikolic, A., Zunec, S., Karaconji, I. B., Juric, A., et al. (2018). Irinotecan and Delta(9)-Tetrahydrocannabinol Interactions in Rat Liver: A Preliminary Evaluation Using Biochemical and Genotoxicity Markers. *MOLECULES* 23. doi:10.3390/molecules23061332. |  |
| Cannabis day length | 980 | Westerhuis, W., van Delden, S. H., van Dam, J. E. G., Marinho, J. P. P., Struik, P. C., and Stomph, T. J. (2019). Plant weight determines secondary fibre development in fibre hemp (Cannabis sativa L.). *Ind. Crops Prod.* 139. doi:10.1016/j.indcrop.2019.111493. |  |
| Cannabis day length | 981 | Wickramasinghe, A., Tulloch, A. D., Hayes, R. D., Chang, C.-K., Broadbent, M., Di Forti, M., et al. (2015). Associations between the schizophrenia susceptibility gene ZNF804A and clinical outcomes in psychosis. *Transl. Psychiatry* 5. doi:10.1038/tp.2015.198. |  |
| Cannabis day length | 982 | Yang, R., Berthold, E. C., McCurdy, C. R., Benevenute, S. da S., Brym, Z. T., and Freeman, J. H. (2020). Development of Cannabinoids in Flowers of Industrial Hemp (Cannabis sativa L.): A Pilot Study. *J. Agric. Food Chem.* 68, 6058–6064. doi:10.1021/acs.jafc.0c01211. |  |
| Cannabis day length | 983 | Young, L. E., and Chin-Quee, K. P. (2017). Dosage and Conditioning Period Determine Reward or Aversion to Cannabis-induced Conditioned Place Preference in Sprague-Dawley Rats. *WEST INDIAN Med. J.* 66, 523–528. doi:10.7727/wimj.2017.185. |  |
| Cannabis day length | 984 | Zhang, Q., Chen, X., Guo, H., Trindade, L. M., Salentijn, E. M. J., Guo, R., et al. (2018). Latitudinal Adaptation and Genetic Insights Into the Origins of Cannabis sativa L. *Front. Plant Sci.* 9. doi:10.3389/fpls.2018.01876. |  |
| Cannabis yield photoperiod | 985 | Amaducci, S., Colauzzi, M., Bellocchi, G., Cosentino, S. L., Pahkala, K., Stomph, T. J., et al. (2012). Evaluation of a phenological model for strategic decisions for hemp (Cannabis Sativa L.) biomass production across European sites. *Ind. Crops Prod.* 37, 100–110. doi:10.1016/j.indcrop.2011.11.012. |  |
| Cannabis yield photoperiod | 986 | Amaducci, S., Colauzzi, M., Bellocchi, G., and Venturi, G. (2008). Modelling post-emergent hemp phenology (Cannabis sativa L.): Theory and evaluation. *Eur. J. Agron.* 28, 90–102. doi:10.1016/j.eja.2007.05.006. |  |
| Cannabis yield photoperiod | 987 | Backer, R., Schwinghamer, T., Rosenbaum, P., McCarty, V., Eichhorn Bilodeau, S., Lyu, D., et al. (2019). Closing the yield gap for cannabis: A meta-analysis of factors determining cannabis yield. *Front. Plant Sci.* 10, 495. doi:10.3389/fpls.2019.00495. |  |
| Cannabis yield photoperiod | 988 | Bauerle, W. L., McCullough, C., Iversen, M., and Hazlett, M. (2020). Leaf Age and Position Effects on Quantum Yield and Photosynthetic Capacity in Hemp Crowns. *PLANTS-BASEL* 9. doi:10.3390/plants9020271. |  |
| Cannabis yield photoperiod | 989 | Bilodeau, S. E., Wu, B.-S., Rufyikiri, A.-S., MacPherson, S., and Lefsrud, M. (2019). An Update on Plant Photobiology and Implications for Cannabis Production. *Front. Plant Sci.* 10. doi:10.3389/fpls.2019.00296. |  |
| Cannabis yield photoperiod | 990 | Cosentino, S. L., Testa, G., Scordia, D., and Copani, V. (2012). Sowing time and prediction of flowering of different hemp (Cannabis sativa L.) genotypes in southern Europe. *Ind. Crops Prod.* 37, 20–33. |  |
| Cannabis yield photoperiod | 991 | Faux, A.-M., Draye, X., Lambert, R., d’Andrimont, R., Raulier, P., and Bertin, P. (2013). The relationship of stem and seed yields to flowering phenology and sex expression in monoecious hemp (Cannabis sativa L.). *Eur. J. Agron.* 47, 11–22. doi:10.1016/j.eja.2013.01.006. |  |
| Cannabis yield photoperiod | 992 | Folina, A., Roussis, I., Kouneli, V., Kakabouki, J., Karidogianni, S., and Bilalis, D. (2019). OPPORTUNITIES FOR CULTIVATION OF MEDICAL CANNABIS (Cannabis saliva L.) IN GREECE. *Sci. Pap. A-AGRONOMY* 62, 293–300. |  |
| Cannabis yield photoperiod | 993 | Hall, J., Bhattarai, S. P., and Midmore, D. J. (2012). Review of Flowering Control in Industrial Hemp. *J. Nat. FIBERS* 9, 23–36. doi:10.1080/15440478.2012.651848. |  |
| Cannabis yield photoperiod | 994 | Hall, J., Bhattarai, S. P., and Midmore, D. J. (2013). The Effects of Different Sowing Times on Maturity Rates, Biomass, and Plant Growth of Industrial Fiber Hemp. *J. Nat. FIBERS* 10, 40–50. doi:10.1080/15440478.2012.756639. |  |
| Cannabis yield photoperiod | 995 | Hall, J., Bhattarai, S. P., and Midmore, D. J. (2014). The Effects of Photoperiod on Phenological Development and Yields of Industrial Hemp. *J. Nat. FIBERS* 11, 87–106. doi:10.1080/15440478.2013.846840. |  |
| Cannabis yield photoperiod | 996 | Lata, H., Chandra, S., Mehmedic, Z., Khan, I. A., and ElSohly, M. A. (2012). In vitro germplasm conservation of high Delta(9)-tetrahydrocannabinol yielding elite clones of Cannabis sativa L. under slow growth conditions. *ACTA Physiol. Plant.* 34, 743–750. doi:10.1007/s11738-011-0874-x. |  |
| Cannabis yield photoperiod | 997 | Lisson, S. N., and Mendham, N. J. (2000). Cultivar, sowing date and plant density studies of fibre hemp (Cannabis sativa L.) in Tasmania. *Aust. J. Exp. Agric.* 40, 975–986. doi:10.1071/EA99130. |  |
| Cannabis yield photoperiod | 998 | Lisson, S. N., Mendham, N. J., and Carberry, P. S. (2000). Development of a hemp (Cannabis sativa L.) simulation model 2. The flowering response of two hemp cultivars to photoperiod. *Aust. J. Exp. Agric.* 40, 413–417. doi:10.1071/EA99059. |  |
| Cannabis yield photoperiod | 999 | Rich, L. N., Ferguson, E., Baker, A. D., and Chappell, E. (2020). A review of the potential impacts of artificial lights on fish and wildlife and how this may apply to cannabis cultivation. *Calif. FISH GAME* 106, 75–91. |  |
| Cannabis yield photoperiod | 1000 | Salentijn, E. M. J., Petit, J., and Trindade, L. M. (2019). The Complex Interactions Between Flowering Behavior and Fiber Quality in Hemp. *Front. Plant Sci.* 10. doi:10.3389/fpls.2019.00614. |  |
| Cannabis yield photoperiod | 1001 | Saloner, A., and Bernstein, N. (2020). Response of Medical Cannabis (Cannabis sativa L.) to Nitrogen Supply Under Long Photoperiod. *Front. Plant Sci.* 11. doi:10.3389/fpls.2020.572293. |  |
| Cannabis yield photoperiod | 1002 | Tang, K., Struik, P. C., Yin, X., Thouminot, C., Bjelkova, M., Stramkale, V., et al. (2016). Comparing hemp (Cannabis sativa L.) cultivars for dual-purpose production under contrasting environments. *Ind. Crops Prod.* 87, 33–44. doi:10.1016/j.indcrop.2016.04.026. |  |
| Cannabis yield photoperiod | 1003 | Zanetti, F., Monti, A., and Berti, M. T. (2013). Challenges and opportunities for new industrial oilseed crops in EU-27: A review. *Ind. Crops Prod.* 50, 580–595. doi:10.1016/j.indcrop.2013.08.030. |  |
